# Arm-less mitochondrial tRNAs conserved for over 30 millions of years in spiders

**DOI:** 10.1186/s12864-019-6026-1

**Published:** 2019-08-23

**Authors:** Joan Pons, Pere Bover, Leticia Bidegaray-Batista, Miquel A. Arnedo

**Affiliations:** 1 Departamento de Biodiversidad y Conservación, Instituto Mediterráneo de Estudios Avanzados (CSIC-UIB), Miquel Marquès, 21, 07190 Esporles, Illes Balears Spain; 2 ARAID Foundation – IUCA Grupo-Aragosaurus, Facultad de Ciencias, Universidad de Zaragoza, Pedro Cerbuna 12 -, 50009 Zaragoza, Spain; 3 Departamento de Biodiversidad y Genética, Instituto de Investigaciones Biológicas Clemente Estable, Avenida Italia 3318, 11600 Montevideo, CP Uruguay; 4 Departament de Biologia Evolutiva, Ecologia i Ciències Ambientals & Institut de Recerca de la Biodiversitat (IRBio), Universitat de Barcelona, Av. Diagonal 643, E-8028 Barcelona, Catalonia Spain

**Keywords:** Reduced mitogenome, Secondary structure RNA, Miss-pairing structures, *Harpactocrates*, *Parachtes*

## Abstract

**Background:**

In recent years, Next Generation Sequencing (NGS) has accelerated the generation of full mitogenomes, providing abundant material for studying different aspects of molecular evolution. Some mitogenomes have been observed to harbor atypical sequences with bizarre secondary structures, which origins and significance could only be fully understood in an evolutionary framework.

**Results:**

Here we report and analyze the mitochondrial sequences and gene arrangements of six closely related spiders in the sister genera *Parachtes* and *Harpactocrates*, which belong to the nocturnal, ground dwelling family Dysderidae. Species of both genera have compacted mitogenomes with many overlapping genes and strikingly reduced tRNAs that are among the shortest described within metazoans. Thanks to the conservation of the gene order and the nucleotide identity across close relatives, we were able to predict the secondary structures even on arm-less tRNAs, which would be otherwise unattainable for a single species. They exhibit aberrant secondary structures with the lack of either DHU or TΨC arms and many miss-pairings in the acceptor arm but this degeneracy trend goes even further since at least four tRNAs are arm-less in the six spider species studied.

**Conclusions:**

The conservation of at least four arm-less tRNA genes in two sister spider genera for about 30 myr suggest that these genes are still encoding fully functional tRNAs though they may be post-transcriptionally edited to be fully functional as previously described in other species. We suggest that the presence of overlapping and truncated tRNA genes may be related and explains why spider mitogenomes are smaller than those of other invertebrates.

**Electronic supplementary material:**

The online version of this article (10.1186/s12864-019-6026-1) contains supplementary material, which is available to authorized users.

## Background

Metazoan mitogenomes are generally composed of 37 genes (13 PCGs, 22 tRNAs and two rRNAs) plus at least one control region [1]. Gene orders vary greatly at higher taxonomic levels, including classes, orders, and families (e.g. [2–4]) but they are usually conserved or involve only rearrangements of tRNA genes at the genus level (e.g. [5, 6]). Additionally, mitogenomes share some distinctive features such as A + T richness, particularly at third codon sites and control region [7], and conservation of secondary structures of tRNAs and rRNAs, despite primary sequences may vary greatly across taxonomic groups and even between close relatives [5, 6]. In the last decade, advances in molecular biology and next generation sequencing technologies have accelerated the sequencing of complete mitogenomes of thousands of eukaryotic species. As March 2018, 7770 animal mitogenomes were available on the NCBI database. However, a closer examination reveals that taxon sampling is extremely biased towards vertebrates. In particular, fishes, mammals and birds are over-represented (2579, 1066, and 618 mitogenomes, respectively). Conversely, mega-diverse taxa, such as insects and crustaceans have relative few mtDNAsin the public repository (1392 and 287, respectively). Spiders are an extreme example of a highly diverse group, which is poorly represented. Only 32 mitogenome sequences are available at the organelle genome database at NCBI out of the over 45,000 species currently accepted, representing only 15 out of the 112 spider families [8].

**Fig. 1:**
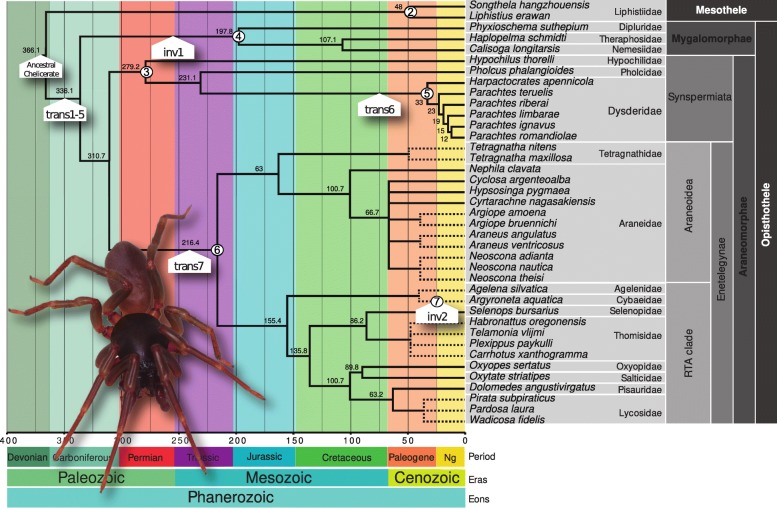
Time stamped phylogenetic tree of spiders, including only those species with mitogenomes generated in the present study or available in public depositories, with the corresponding taxonomical hierarchy. The molecular mechanisms of gene rearrangements (transposition and inversion) originating the present gene orders in spiders were estimated by TreeREx and they are indicated on nodes of Fig. 1 and explained in Fig. 3. Node numbers in circles indicate a particular gene order which is described in detail in Fig. 3. The topology and the divergence time estimates were obtained from the recent phylogenomic study by Fernández et al. 2018. Relationships and timing for the genus *Parachtes* and *Harpactocrates* as inferred in Bidegaray et al. 2011. The dashed branches correspond to lineages with no available divergence time estimates

Most mitogenomic studies usually focus on the use of the nucleotide and amino acid sequences as phylogenetic markers to infer fully resolved and well-supported evolutionary relationships among target taxa or test the statistical support of higher taxonomic ranks, e.g. [9–11]. These studies have shed light on the diversification of poorly known lineages and addressed questions about the biogeography, the timing of diversification and trait evolution, e.g. [12–14]. Some other mitogenomic studies have payed attention to the evolution of this reduced genome itself, addressing questions about the frequencies of nucleotide and amino acid sequences, codon usage, secondary structures of RNA genes and compensatory substitutions, and control regions and origin of replication across genes and species, e.g. [5, 15, 16]. In this study, we specifically interrogate on aspects of the secondary structure of mitochondrial tRNAs in spiders, by sequencing the mitogenomes of several representatives of the family Dysderidae, which belongs to the Synspermiata [17–19], one of the main evolutionary lineages within spiders (Fig. 1). Although, Synspermiata includes 17 families, only one mitogenome is currently available in public organelle databases, that of the common cellar spider *Pholcus phalangioides* (Pholcidae).

Generally, metazoan tRNAs fold in a typical cloverleaf structure with four arms (acceptor, DHU, anti-codon and TΨC) except for tRNA-S1_GCT_ that lost the DHU-arm in nearly all metazoans [20]. This cloverleaf structure is most likely conserved across metazoans by selective constraints so that they can effectively interact with other elements of the translation machinery [21]. Nonetheless, spider tRNAs are exceptional in this regard since most tRNAs lack the TΨC arm, and fewer the DHU arm, in both cases simplified down to TV-replacement loops, e.g. [21–23]. The absence of arms in the secondary structure of tRNAs was first described in nematodes decades ago [24] and was suggested to be the result of the co-evolution of ribosomal RNA and RNA-binding proteins [25]. Subsequently, several studies described this atypical tRNA structures in chelicerates (mites, spiders, scorpions, pseudoscorpions, and whip scorpions), hexapods (insects and proturans), and parasitic thorny-headed worms (Acanthocephala), see [26]. The trace of TΨC arm loss on a phylogenetic tree including the main arachnid groups suggests a parallel evolution of this event, i.e. evolved multiple times independently, with a propensity of loss of cloverleaf tRNAs, and when TΨC is lost then it is not regained [21]. On the other hand, some arm losses seem to be synapomorphies, i.e. derived shared characters, such as the loss of DHU-arm in *trnS2* of the spiders of the suborder Opisthothelae, although additional taxon sampling is needed to confirm this evolutionary pattern [22]. In addition, other works revealed the existence of many mismatches and G–U wobble pairs in the acceptor stem in several unrelated spider species, particularly in opisthothelae spiders [21, 22, 27, 28]. Finally, several studies had already reported that degeneration of tRNAs may go even further by losing both DHU and TΨC arms, the so-called arm-less tRNAs [29]. For instance, the spider mite genus *Tetranychus* carries three arm-less tRNA genes (*trnI*, *trnP* and *trnQ* [30]), the human follicle mites *Demodex folliculorum* and *Demodex brevis* five (*trnA*, *trnD*, *trnR*, *trnS2* and *trnT* [31]), the oribatid- mite *Paraleius leontonychus* (*trn*A and *trn*V [32]), and the *Steganacarus magnus* (*trnC* [33]). The loss of DHU and/or TΨC arms in conjunction of the presence of several miss-pairings in the acceptor arm have precluded the automatic annotation of several tRNAs in some species which later on have been detected manually [33].

Here we report the shortest arm-less tRNA genes described in spiders, and probably in the whole animal kingdom, to date. We discovered this noticeable feature while annotating mitogenomes of the spider genera *Parachtes* Alicata, 1964 and *Harpactocrates* Simon, 1914, endemic to the Mediterranean region, as part of a larger study aimed at resolving their phylogenetic relationships and evolutionary history [34, 35]. The comparative analyses carried out on the closely related *Parachtes* and *Harpactocrates* genera enabled an accurate annotation of the mitochondrial tRNAs that had previously defied identification by using automatic algorithms, and further provided a comparative framework to infer the secondary structure of tRNAs.

## Results

### Mitogenome assembly and annotation

After quality trimming, about 90% of the reads of a Roche FLX/454 run were assembled into five mitochondrial contigs, corresponding to the following species: *Parachtes teruelis* (Kraus, 1955) (27,459 reads), *Parachtes romandiolae* (Caporiacco, 1949) (60,976), *Parachtes limbarae* (Kraus, 1955) (28,728), *Paracthes ignavus* (Simon, 1882) (61,072), and *Harpactocrates apennicola* Simon, 1914 (30,302). The total length of the mitogenomes was around 14 kb with a coverage depth of above 200x and read length ranging from 350 to 550 bp. The coverage depth of *Parachtes riberai* Bosmans, 2017 was only 2-5x since nucleotide sequences were obtained by sequencing the inserts of 96 clones by the Sanger method and filling gaps by primer walking sequencing. Three mitogenomes were circularized, *P. romandiolae* (14,220 bp), *P. limbarae* (14,111 bp), and *H. apennicola* (14,213 bp), but we failed to circularize (i.e. incomplete) *P. riberai* (14,632 bp) and *P. ignavus* (14,667 bp) due to the presence of an extremely long polyA run in the control region. The same issue precluded to obtain most of the control region in *P. teruelis* (13,850 bp).

**Fig. 2:**
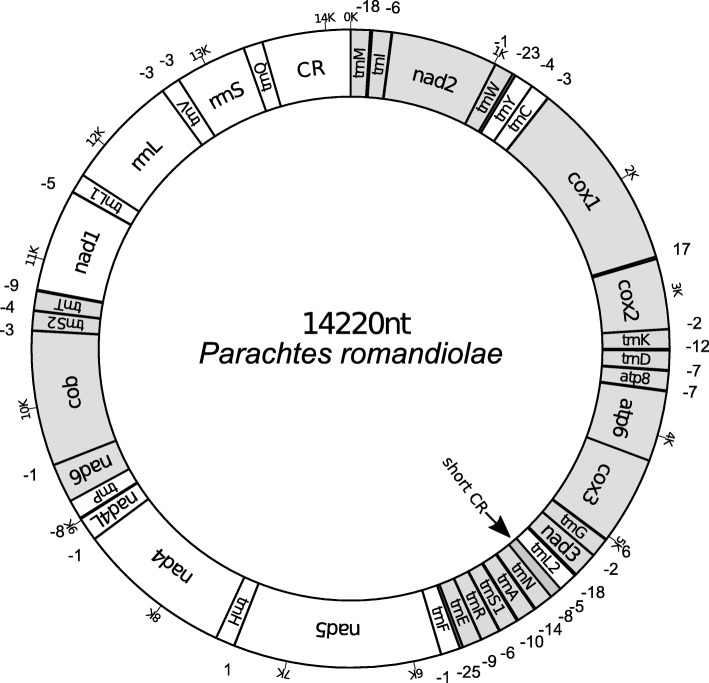
Map of the mitogenome of *Parchtes romandiolae*. Genes coded on the plus strand are indicated in grey whereas genes on minus strand are not colored. Outer negative numbers indicate overlapping nucleotide positions between genes and positive values non-coding positions

**Fig. 3:**
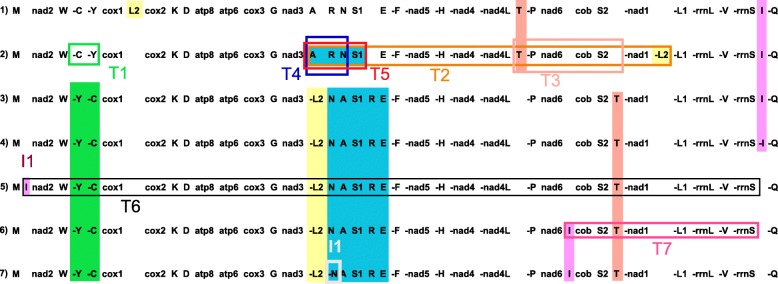
Comparison of the four main mitochondrial gene orders found in the 32 spider mitogenomes available in GenBank and MetAMiGA plus that retrieved from *Parachtes* and *Harpactocrates* (2). Genes placed on minus strand are denoted with the symbol “-”. Rearranged genes gene blocks are highlighted in different colors: transpositions (T) and inversions (I). Gene orders are as follows: 1) ancestral Pancrustacean (Boore et al. 1995), 2) Mesothelae and ancestral for Chelicerata and Arthropoda (Staton et al. 1997), 3) Araneomorphae, Synspermiata, Pholcidae and Hypochilidae, 4) Mygalomorphae, 5) Araneomorphae, Synspermiata, Dysderoidea, 6) Araneomorphae, Entelegynae, and 7) Araneomorphae, Entelegynae (Cybaeidae only). See Additional file 5: Table S1 for a detailed report of the mitogenome gene orders and accession numbers for the 32 mitogenomes

The mitogenomes of the six spiders studied here coded the 37 genes commonly found in most metazoan species, 13 PCGs, 22 tRNAs and 2 rRNAs, plus a large control region (Fig. 2). The gene order found in *Parachtes* and *Harpactocrates* is a new discovery in spiders and it seems to arise from a translocation involving *trnI* and a large gene block (*nad2* to *rrnS;* see Figs. 1, 2, 3). The gene rearrangements and mechanisms inducing the different gene orders of the spider mitogenomes available in public databases are depicted in Fig. 3. Twenty-two genes were coded on the majority strand (plus strand) and 15 on the minority one (minus strand, Fig. 2). Most genes, particularly tRNA-coding genes, shared positions with flanking sequences and consequently there were few non-coding spacers (Fig. 2). The largest compacted region was composed of the genes *trnN*, *trnA*, *trnS1*, *trnR*, *trnE*, and *trnF* with a total of 73 overlapping positions whereas the largest non-coding spacer was 17 bp long and laid between *cox1* and *cox2* genes.

### Nucleotide frequencies and skews

Mitogenomes of *Parachtes* and *Harpactocrates* were A + T rich (ca 71%) as expected for arthropods and most metazoans (Additional file 1: Figure S1). A similar bias was found regardless of how mitogenomes were partitioned, i.e. by gene, by function (e.g. PCG/RNA coding), or by codon position (Additional file 1: Figure S1). The A + T contents estimated in the six target spiders was close to the average values estimated for 12 complete spider mitogenomes available in the MetAMIGA database (mean 70.9%; range 64.0–77.3%), PCG plus strand (69.3%; 62.5–75.6%), PCG minus strand (71.4%; 64.1–78.6%), tRNA (72.6%; 67.1–79.9%), and rRNA (73.9%; 66.8–80.6%). Third codon sites of PCGs, ribosomal genes, and control regions showed the higher A + T content (> 75%). Conversely, first and second codon sites showed lower A + T contents particularly the latter (Additional file 1: Figure S1). A further exploration showed that PCGs, but not RNA genes, differed in A + T richness depending on which strand were coded. PCGs coded on the minus strand showed higher A + T frequencies in first codon positions that those coded on the plus strand, ca 70% vs 62%, respectively. Contrarily, third codon position of plus strand oriented PCGs were slightly richer in the A + T content with respect to those on the minus one, ca 78% vs 75% (Additional file 1: Figure S1). Variation across the six target species in all patterns were marginal, with *H. appenicola* the most divergent (yellow diamonds on Additional file 1: Figure S1).

In spite of their function, genes coded on the plus and minus strand showed different trends in intra-strand AT and GC frequencies (i.e. AT and GC skews). Negative ATskew values were found in those genes coded on plus strand, and positive numbers on minus strand (0–0.25) with the exception of the minus strand second codon positions which displayed negative values (up to − 0.45; Additional file 1: Figure S1). Conversely, GC skew values were slightly positive for genes on the plus strand (up to 0.8), and slightly negative (up to − 0.8) for minus-strand oriented genes. Exceptions to this trend were the tRNAs on the minus strand and the second codon position of PCGs of the plus strand (Additional file 1: Figure S1).

### Amino acid frequencies and start and stop codons

In *Parachtes* and *Harpactocrates*, less than half of the PCGs started with the canonical ATA (21) and ATG (9) codons, while the remaining showed non-canonical codons (Table 1). Conversely, most stop codons were the canonical TAA (33) and TAG (20) and in few cases the truncated TA (19) or a single T (6) (Table 1). Interestingly, most of the canonical start and stop codons corresponded to those with higher A + T content (ATA and TAA), while a lower frequency of ATG and TAG codons was observed. The translation of the 13 PCGs of the six target species revealed a higher frequency for amino acids L, S, F, and M and lower values for C, H, R, and Q with minimal differences across species (Additional file 2: Figure S2). As expected from the nucleotide pattern above, amino acid frequencies also differed depending on the coding strand (Additional file 2: Figure S2). PCGs coded on the plus strand possessed higher frequencies of V and G and lower values for T and I, while the opposite trend was observed on the minus strand. The analysis of the codon usage also indicated that A + T-rich triplets were more frequent than G + C-rich codons (data not shown). Finally, the alignment of PCGs at the protein level showed that insertions and deletions were rare and included events of one or two amino acids in *cox2*, *cob*, *nad1*, *nad5*, and *nad6*; a single event involving three positions in *cob*, and another one with four in *nad5*.

**Table 1:** Length, and start and stop codons for the 13 protein coding genes (PCGs) of the six species studied here

	*P. teruelis*			*P. riberai*			*H. apennicola*			*P. romandiolae*			*P. limbarae*			*P. ignavus*		
*nad2*	927	ATA	TAA	927	ATT	TAG	926	ATT	TA	927	ATT	TAA	926	ATT	TA	927	ATT	TAA
*cox1*	1533	TTA	TAA	1533	ATA	TAG	1533	TTA	TAG	1533	GTA	TAG	1533	GTA	TAA	1533	GTA	TAG
*cox2*	658	ATT	T	660	GTG	TAG	658	GTG	T	660	GTG	TAG	660	ATA	TAG	657	GTG	TAA
*atp8*	150	ATA	TAA	150	ATT	TAA	150	ATT	TAA	150	ATT	TAA	150	ATT	TAA	150	ATT	TAA
*atp6*	666	ATG	TAG	666	ATG	TAG	666	TTG	TAG	666	ATG	TAG	666	ATG	TAA	666	ATG	TAA
*cox3*	789	ATA	TAA	789	ACA	TAA	788	AAA	TA	789	GTA	TAG	789	TTA	TAA	789	ATA	TAG
*nad3*	336	ATA	TAA	336	ATA	TAA	336	ATT	TAA	336	ATT	TAA	336	CTG	TAA	336	ATT	TAA
*nad5*	1649	ATT	TA	1650	ATC	TAA	1644	AAT	TAA	1649	ATT	TA	1637	ATT	TA	1650	ATC	TAA
*nad4*	1277	ATA	TA	1278	ATA	TAA	1277	ATA	TA	1276	ATA	T	1277	ATA	TA	1276	ATA	T
*nad4L*	263	ATA	TA	263	ATT	TA	263	ATT	TA	263	ATC	TA	263	ATA	TA	263	ATT	TA
*nad6*	433	ATA	T	434	ACA	TA	436	TTG	T	434	TTA	TA	434	TTG	TA	434	TTA	TA
*cob*	1134	GTG	TAG	1128	ATG	TAG	1137	GTG	TAA	1128	ATG	TAG	1128	ATG	TAG	1128	ATG	TAG
*nad1*	903	ATA	TAA	903	ATT	TAA	909	ACT	TAG	903	ATA	TAA	906	ATA	TAA	903	ATA	TAA

Truncated stop codons correspond to “T” and “TA”

### Secondary structure of tRNAs

**Fig. 4:**
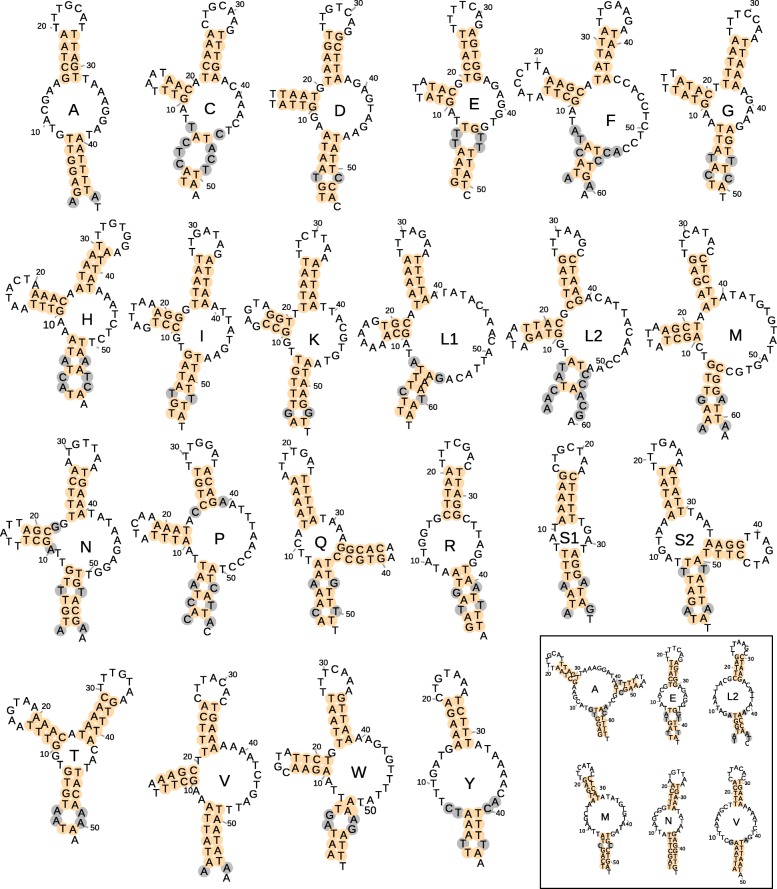
Secondary structures predicted for the 22 tRNAs of *Parachtes romandiolae*. Panel on bottom right side shows alternative secondary structures for tRNAs A, E, L2, N and V

Very few original tRNA foldings obtained by Infernal-MiTFi (Additional file 3: Figure S3) were retrieved as cloverleaf structures, and if present, sequences including DHU or TΨC arms were different in length and nucleotide identity (i.e. not conserved across the six spider species). The retrieval of divergent secondary structures among those closely related species was due to the presence of many mismatches in the acceptor arms which impeded an accurate closure of the tRNA molecule. Therefore, we used the conserved nucleotide sequences of the acceptor arms and their respective secondary structures to build tRNAs that either lacked TΨC or DHU arms or were completely arm-less (Additional file 4: Figure S4). The detailed analyses and reconstruction of the secondary structures showed that none of the 22 tRNAs of the six spiders could be folded into a classical cloverleaf secondary structure with four arms (Fig. 4). Eighteen tRNAs showed the loss of one arm, either the TΨC (C, D, E, F, G, H, I, K, L1, L2, N, M, P, T, V, and W) or the DHU (Q and S2), while the remaining four lacked both arms (A, R, S1, and Y; Fig. 4). The program MiTFi in MITOS2 only recovered two arm-less tRNAs (A in *P. romandiolae* and S1 in *P. riberai*). However, for the tRNAs A, R, S1, and Y neither DHU nor TΨC were present or conserved across species suggesting that they could be folded as arm-less tRNAs (see Fig. 4 and Additional file 4: Figure S4). For instance, tRNA A shows an extremely large TΨC stem composed of eight not fully matching pairs that in most species neither form the compulsory straight angle with the acceptor arm (i.e. no bases between both arms). All those circumstances pointed out that tRNA A could be folded as an arm-less tRNA with an acceptor arm having six or seven matching pairs. The alternative secondary structures for R, S1 and Y having a TΨA arm are also reported in Additional file 4: Figure S4. For other five tRNAs (E, L2, M, N and V) despite conserving a DHU arm, their alternative arm-less structure is also plausible (see Additional file 4: Figure S4). Interestingly, acceptor stems from those arm-less tRNAs show more matching pairs than their counterpart structures with DHU arms particularly for tRNAs N and M. Another hint that suggest that N and E could be arm-less structures is that they form part of a large tRNAs block (*nad3*-L2-CR-N-A-S1-R-E-F-*nad5*) including three “true” arm-less tRNAs (A-S1-R). In addition, the acceptance of an arm-less secondary structure in N and E would reduce the overlapping with other genes in this tRNA block (from 73 to 53 bp). Another evidence supporting an arm-less structure for tRNA N is that there is a conserved mismatch in the first pair of DHU stem in all six species. Such mismatch implies that the canonical two bases separating acceptor and DHU arms are increased to three nucleotides and the single base separating DHU and anticodon arms are incremented to 2 bp, hence reporting an incoherence with the canonical structure of tRNAs. Regardless of the number of arms, mismatches and GU pairings were rare in the anticodon arm, i.e. mostly AU and GC bounds. In DHU and TΨC arms, if present, the mismatches in stems were also rare but they did contain GU pairings. Among those lacking both DHU and TΨC arms, *trnS1* gene was the most extreme example of shortening since it was just 39 bp long in *P. riberai* although it overlaps 10 bp with adjacent *trnA* and 6 pb with *trnR*. Finally, most tRNAs of *Parachtes* and *Harpactocrates* usually displayed two or three mismatches in the acceptor stem, and in few extreme cases four out of the seven canonical pairs (see Fig. 4 and Additional file 4: Figure S4).

### Ribosomal RNA structures

**Fig. 5:**
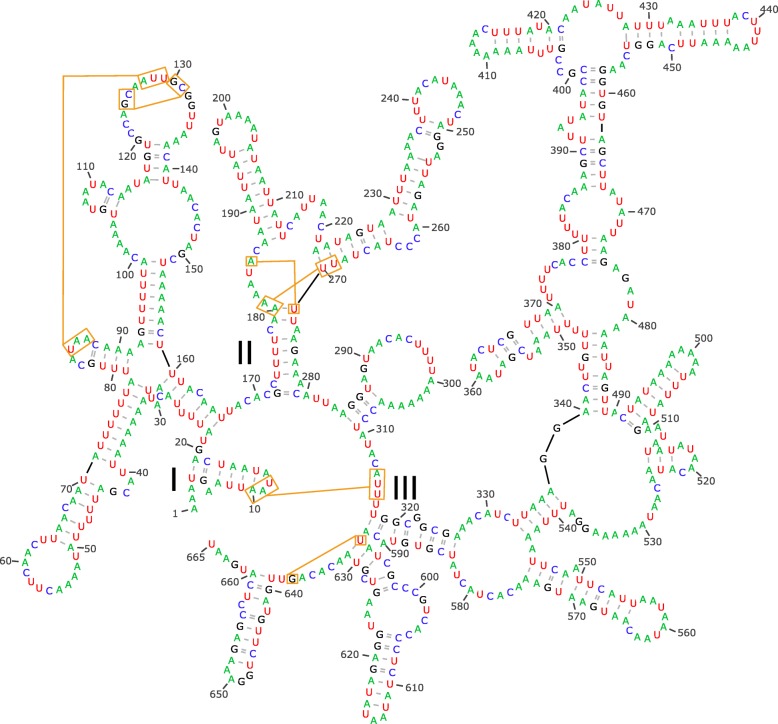
Predicted secondary structure of the small ribosomal unit (12S rRNA) of *Parachtes romandiolae.* Roman numerals indicate different domains. Yellow lines and boxes show positions deduced to be involved in tertiary folding

**Fig. 6:**
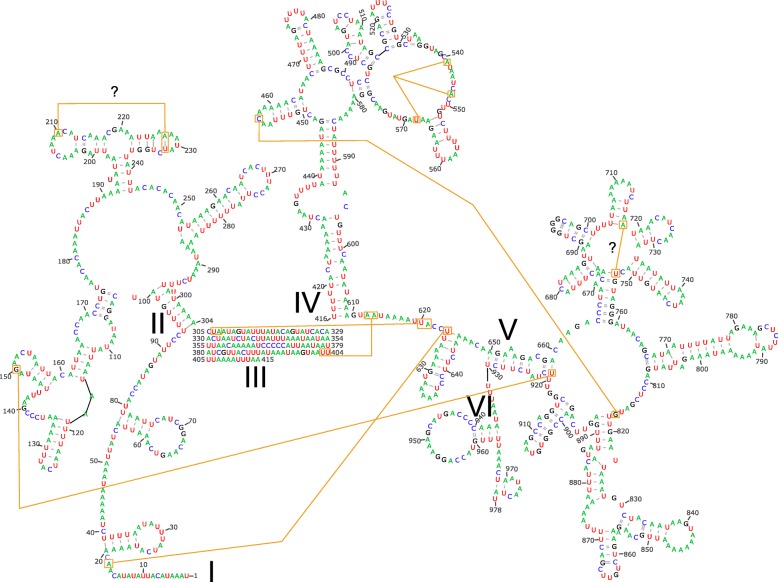
Predicted secondary structure of the large ribosomal unit (16S rRNA) of *Parachtes romandiolae.* Roman numerals indicate different domains. Yellow lines and boxes show positions deduced to be involved in tertiary folding

The domain III of the small rRNA subunit (rRNA 12S) was the most conserved region at the nucleotide identity level in *P. romandiolae* compared to the structures described elsewhere (see Discussion). Therefore, the predicted secondary structure of this domain highly resembled the folding predicted in other species and required minor manual corrections (Fig. 5). On the other hand, few segments of domains I and II showed identity at the nucleotide level and their secondary structures were divergent to those published for other species (see Discussion). Therefore, the folding in both domains had to be predicted from the few stretches with high similarity from which we could reconstruct the expected secondary structures or similar ones. The conserved stretches (positions 1–54, 76–139, 155–187, 245–274, and 304–318, see Fig. 5) alternated with large insertions and deletions of up to 30 bp long. Each short motif (20–30 bp) was folded individually in Mfold, starting from the most conserved regions. Interestingly, the most conserved nucleotide stretches included those positions that play a key role in the tertiary folding of the rRNA 12S molecule (yellow lines in Fig. 5). The secondary structure of 16S revealed itself trickier, since stretches from different domains were paired together in the structures estimated in MITOS2. Contrary to rRNA 12S, the sequence of the large ribosomal subunit (rRNA 16S, Fig. 6) showed less and shorter insertions and deletions, but higher nucleotide divergence across species. Domain V of rRNA 16S was the most conserved fragment at the nucleotide level and hence the secondary structure predicted in MITOS2 was similar to that published for other species. On the other hand, and despite of partial conservation in domain I at the nucleotide level, other domains had to be folded by short motifs since the lack of global similarity joined motifs from different domains. The domain III, which is absent in Pancrustacea, was easy to recognize since 5′ and 3′ end stretches were conserved at the nucleotide levels and it has no secondary structure.

### Control region

The main control region was located between genes *trnQ* and *trnM*. Because of long polyA stretches and repetitive fragments, it could be fully sequenced only in three of the six species. The control region length was 786 and 714 bp in *P. romandiolae* and *P. limbarae*, respectively, and 747 bp in *Harpactocrates.* Sequences in all three species were also A + T rich (73.03, 75.35, and 73.5%, respectively), but not as high as described for most Pancrustacea species, which can reach values above 90%. The sequence of the main control region of the two *Parachtes* species was also conserved at nucleotide level particularly a region of 138 bp with 74% of similarity and 9% of gaps (positions 335–470 and 353–499 in *P. romandiolae* and *P. limbarae*, respectively). A shorter stretch of 81 bp in the same region was also conserved in *H. apennicola* (positions 284–369, 48–49% sequence identity and several indels). The main control region of the 3 species showed several palindromic regions, which could correspond to the putative origin of replication given the presence of a characteristic long poly A stretch (~ 10–25 bp), although the ATAT (TATA) and GA(N) T motifs adjacent to 5′ and 3′ ends were missing. Some of these palindromic regions were recognized as putative origin of replication of plus strand in MITOS2, but neither their locations nor their nucleotide sequences were conserved across the 3 species.

**Fig. 7:**
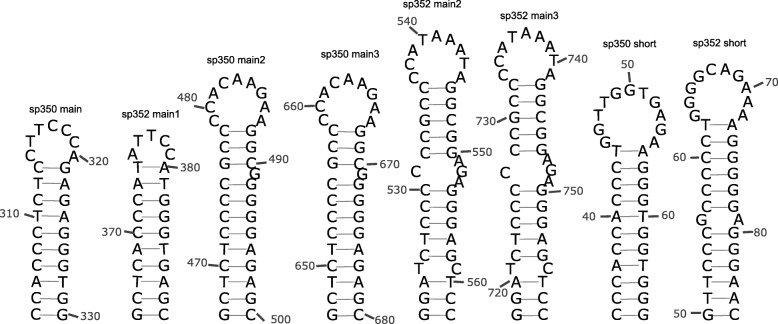
Secondary structure predicted for different putative origin of replication found in the main control region and in the short non-coding region of *Parachtes romandiolae* (352) and *Harpactocrates apennicola* (350)

The six spiders studied here showed an additional short conserved non-coding sequence between *trnL2* and *trnN* genes (85–94 pb in length). Nucleotide identity among the five species of the genus *Parachtes* ranged between 51 and 81%. The coverage and similarity of this short region relative to *H. apennicola* was about 50% for both features. This short non-coding region also harbors a palindromic region (Fig. 7) that MITOS2 recognized as putative origin of replication for the lagging strand during replication (minus strand). However, the detection of the putative origin of minus strand was not positive in all species despite the presence of conserved poly G/C stretches. Interestingly, the main control region of *P. romandiolae* harbored a duplicated unit of 79 bp with a nucleotide identity of 96 and 2% of gaps. This region included a palindrome with GC stretches that resemble the putative origin of replication found in the short non-coding region located between *trnL2* and *trnN* (Fig. 7). This long motif is also found in the main control region of *P. limbarae* and *H. apennicola.*

## Discussion

Our study reveals that *Parachtes* and *Harpactocrates* spiders have taken tRNAs reduction to an extreme, since none of the 22 tRNAs had a cloverleaf structure and at least four of them lack both DHU and TΨC arms. Actually, the 39 bp long tRNA-S1 gene (*trnS1*) of *P. romandiolae* is 3 bp shorter than the tRNA-R of the nematode *Romanomermis culicivorax* [36], setting a new record for the shortest tRNA ever described. The *Parachtes* and *Harpactocrates* tRNA-S1 reduction further involves the acceptor stem, which shows multiple mismatches. This pattern has been also described in the orb-weaver spiders *Neoscona doenitzi* and *Neoscona nautica* (Araneomorphae, Araneidae [22]), and the bird spider *Ornithoctonus huwena* (Mygalomorphae, Theraphosidae [28]), and hence those mismatches in acceptor arm may be a shared feature among opistothelid spiders [21]. Some of the tRNAs in *Parachtes* and *Hapactocrates* have as little as three pair matches in their acceptor arm as previously described in four tRNA genes in *O. huwena* (*trnF*, *trnK*, *trnL1* and *trnL2* [28]). In fact, the existence of six mismatches in the acceptor arm of the tRNA-H of the crustacean *Armadillidium vulgare* already posed serious difficulties to their first detection and annotation, and were finally detected by RT-PCR of circularized RNAs and subsequent cloning [37]. The sequencing of clones showed that mature tRNA-H had the base-pairing of the acceptor stem fully restored by nucleotide replacement at the 3′-end, and that there was an additional CCA triplet at the 3′ end and a G-1 guanosine residue at the 5′ end, thus confirming that *A. vulgare encoded a fully functional copy of tRNA-H* [37].

The absence of both DHU and TΨC arms and presence of mismatches in the acceptor arm, compromised an accurate annotation of tRNAs in our six spiders, even using extremely low Cove cutoff scores (X < − 50) in tRNAscan-SE or e-values in Infernal-MiTFi within MITOS2. The accurate folding of *Parachtes* and *Harpactocrates* arm-less tRNAs could only be achieved manually thanks to the highly conserved nucleotide sequences across the species, particularly the anticodon arm, which facilitated the detection of a common underlying secondary structure. The “standard” tRNAs lacking DHU or TΨC arms were always detected by MITOS2 and tRNAscan-SE in the six species with perfect pairing in their DHU or TΨC stems. In fact, the inconsistent presence or absence of DHU or TΨC arms in a particular tRNA was a crucial hint to identify both arms. The lack of covariant models searching for arm-less tRNAs and allowing multiple mismatches, could explain why three of the tRNA genes could not be found in the mite *Tyrophagus longior* (*trnF*, *trnS1*, and *trnQ* [38]), or why several arms were shorter and unstable in the mite *Panonychus citri* (TΨC-arms in *trnS2* and *trnV*; and DHU-arms in *trnY*, *trnR*, *trnP* [39]). Finally, the conservation at the nucleotide level across the six species studied here along with the conservation of the genomic position even among the 32 additional spider mitogenomes available in public repositories, suggested that the tRNA genes annotated in *Parachtes* and *Hapactocrates* represented the true set of 22 tRNAs, in spite of displaying aberrant structures. Similarly, a previous study on *Romanomermis* and close allied nematodes, allowed the detection of arm-less tRNAs in this group [40], as well as in the oribatid mite *Paraleius leontonychus* [32] and, with an accurate manual review, the annotation of aberrant tRNAs in previously published mitogenomes of sarcoptiform mites with missing tRNAs [33]. Finally, besides the four tRNAs that are clearly arm-less (A, R, S1, and Y) in *Paractes* and *Harpactocrates*, we think there are several evidences suggesting that tRNAs N, E and M, and even perhaps L2 and V, are also arm-less structures. However, additional experiments are essential to corroborate our hypothesis.

Based on available time estimates that trace back the split between *Parachtes* and *Hapactocrates* to the late Oligocene [34], the multiple mispairing, arm-less tRNAs described here have been preserved for at least 30 million years. This long-term conservation suggests that those tRNA genes are fully functional, and hence these genes encoding deviant secondary structures are under selective constraints. It is known that DHU and TΨC arm-lacking tRNAs may form tertiary inverted L-shape-like structures that contain aminoacylation capacity to interact with the elongation factor EF-Tu, as canonic cloverleaf tRNAs do [25]. Recent studies using RT- PCR analyses, have unveiled that arm-less tRNA genes of the nematode *R. culicivorax* are also transcribed, and include a 3′ CCA sequence as result of interacting with the Aminoacyl tRNA Synthetase, which is a key step in tRNA maturation even for arm-less tRNAs [36]. In fact, the transcripts of these arm-less tRNAs of *R. culicivorax* also fold into a single stable hairpin structure in a tridimensional boomerang-like form that diverges from the standard L-shape structure [41]. Truncated tRNAs have been observed neither in plants nor fungi, and hence it has been postulated that mitochondrial DHU-armless tRNAs are exclusive to metazoans, and that TΨC-armless tRNAs arose several times independently within Eumetazoa [42]. In mammals, cloverleaf and DHU-armless tRNAs are recognized by a single mitochondrial elongation factor (EF-Tu), which resembles the bacterial type. However, in the nematode *Caenorhabditis elegans* a gene duplication of the EF-Tu, namely EF-Tu1 and EF-Tu2, may have played a key role in the degeneration of tRNAs, since they exclusively recognize aminoacylated TΨC- and DHU-armless tRNAs, respectively; see [43]. A recent study also showed that *Drosophila melanogaster*, in spite of not having arm-less tRNAs in its mitochondria, possesses a EF-Tu1 that has the ability to recognize TΨC-armless from other related arthropod species [44]. Based on these observations, it is plausible that the evolution of a new EF-Tu variant may have contributed to the extreme truncation of tRNAs in spiders and mites, since they have both evolved fully functional armless tRNAs. If this holds true, post-transcriptional modifications and editing will have ensured correct folding and efficient translation, and will have served as target for recognition by aminoacyl-tRNA synthetases, as well as an adaptation to different environmental conditions such as temperature [45, 46]. In fact, we suggest that post-transcriptional interactions and coevolution between tRNA terminal nucleotidyltransferases (CCA enzymes) and ribosomes (elongation factor EF-Tu) may have been paramount for ensuring functionality of these shrunken mitochondrial tRNAs [25]. Further studies are required to provide additional corroboration to this suggestion. Alternatively, it has been suggested that functional mitochondrial tRNA could be imported from the cytoplasm into the mitochondria as have been described in some animals [47], and hence replacing the mitochondrial-encoded putative tRNAs lacking both DHU- and TΨC-arms [25].

The gene order found in *Parachtes* and *Harpactocrates* species (Fig. 2 and Fig. 3 line5) differs from those considered ancestral for Chelicerata and Arthropoda (Fig. 3 line 2 [22, 48]), and Pancrustacea (Fig. 3 line 1; [49]), particularly for the position of *trnL2 and trnI* (Fig. 3; Additional file 5: Table S1). Among the additional 32 spider mitogenomes available, the representatives of the suborder Mesothelae, which represent the first offshoot within spiders (Fig. 1, see [18, 19] and references therein), show the most similar gene order to the putative ancestral pattern (Fig. 3 line 2 [22, 28]). In Mesothelae (Fig. 3 line 2), *trnL2* is encoded in the minus strand between *nad1* and *trnL1* whereas the ancestral position, and diagnostic for Pancrustacea (Fig. 3 line 1), is in the plus strand between *cox1* and *cox2* genes. The remaining spiders, the suborders Mygalomorphae and Araneomorphae (Fig. 1), are characterized by the gene shuffling between the adjacent *trnY* and *trnC*, the rearrangement of *trnL2* between *nad3* and *nad5* (*−trnL2*, *trnN*, trn*A*, *trnS1*, *trnE*, *−trnF*) and the new location for the *trnT* gene (Fig. 3 lines 3–6). This pattern seems to have been originated by five different transpositions, order of which is unclear (Fig. 3). The two species in the suborder Mygalomorphae (Fig. 1) show a diagnostic gene arrangement, consisting in just a single inversion of the *trnI* gene (Fig. 3 line 4). The dysderid genera (Fig. 1), *Parachtes* and *Harpactocrates*, differed from all the other mitogenomes examined by the position of the *trnI* gene, which is unique within spiders and originated by a transposition (Fig. 1 and Fig. 3 line 5). Similarly, the Entelegyne spiders (Fig. 1 and Fig. 3 line 6) also show a synapomorphic position of the *trnI* gene, which differs from both the one in the dysderids and the one shared, and putatively ancestral, by the rest of the Araneomorphae, Mygalomorphae (although inverted) and Mesothelae (Figs. 1 and 3).

The mitogenomes of *Parachtes* and *Harpactocrates* are of similar size to the remaining spider mitogenomes deposited in GenBank (average 14.33 ± 0.24 kb) except for *Argyroneta aquatica*, which is approximately 16 kb. Spider mitogenomes are more compact than the average size in other arthropod groups, including myriapods (15.09 ± 0.54), insects (15.63 ± 0.85 kb) and crustaceans (15.84 ± 0.77 kb), and in other invertebrates such as annelids (15.45 ± 1.09 kb), and mollusks (16.80 ± 3.81 kb). A recent study on the short mitogenome of the isopod *A. vulgare* (13.9 kb), which shows overlapping genes and an incomplete and truncated set of tRNA genes, revealed the existence of novel post-transcriptional mechanisms ensuring functionality of overlapping genes such as the post-transcriptional reparation of the truncated tRNA-H explained above [37]. The genome of *A. vulgare* also exhibits large overlaps between two tRNA genes transcribed in the same direction and even tRNA genes either partially or completely placed within a PCG in direct or opposite orientation [37]. The former study suggests that under selective pressure for genome downsizing, an alternative repair system to the punctuation model of RNA processing [50] may develop to ensure fully operational mitochondrial mRNAs and tRNAs. Therefore, aberrant and overlapping DNA sequences coding for tRNA genes and their post-transciptional repair systems should co-evolve to allow mitogenome reduction yet functional tRNAs (i.e. overlapping gene sequences [37]). Moreover, the existence of large non-coding spacers *in A. vulgare*, such as the one reported here between *cox1* and *cox2*, is thought to represent residual sequence artifacts caused by rearrangement events [5, 51]. Interestingly, in the ancestral Pancrustacean pattern, the *trnL2* gene is located between *cox1* and *cox2* whereas in spiders is adjacent to *nad3* or *nad1*.

The mitogenomes of *Parachtes* and *Harpactocrates* were A + T rich (ca 71%), which is consistent with the mean value (73.1%) recently estimated for spiders [22]. Similarly, the asymmetric pattern observed in A + T richness, where PGCs, but not RNA genes, differed in A + T richness depending on which strand were coded, was already described in previous studies on spiders (e.g. [22]) and other invertebrates (e.g. [5]). Similarly, the AT- and GC-skew values and trends observed in the dysderid spiders, have been also reported in both the 32 spider mitogenomes available in the database MetAMIGA [52], as well as in other available spider mitogenomes [22]. A closer inspection confirms that skews in dysderids are similar to those found in other Araneomorphae and also in Mygalomorphae and clearly divergent to those in the suborder Mesothelae. Amino acid usage observed is also similar to those reported in two *Neoscona* orb-weaver spiders [22], and in addition codon usage bias towards A + T-rich codons is consistent with those described in other arthropods [6]. Regarding to the starting non-canonical codons observed in *Parachtes* and *Harpactocrates*, most of them (e.g. ATT, GTG, CTG, and TTA) have been already reported in spiders [22], mites [39], amphipods [53], and beetles [7]. However, the also observed AAA is unique and has not been documented before. In fact, most of those non-canonical codons just differ in a single substitution from the canonical start codons ATA and ATG, while the less frequent AAT and ACT show two nucleotide changes.

RNAs with the same function show a conspicuous similarity in the arrangement of base-paired stems (i.e. topology or secondary structure) in spite of differing in the exact size and spacing of base-paired regions [54]. This is the first study that reports the full secondary structure of both ribosomal RNAs (16S and 12S) encoded in the mitogenome in spiders. Previous studies only reported the structure of the 3′ end of 16S since it was one of the preferred markers for phylogenetic inference [55, 56]. Even though the nucleotide sequences were very divergent, the secondary structures estimated here for *rrnL* and *rrnS* sequences in *P. romandiolae* closely matched the folding predicted in other arachnids such as the whip spiders *Damon diadema* and *Phrynus sp.* (Amblypygi) [57], and six species of *Tetranychus* spiders mites (Acari) [30]. The structures of *rrnL* and *rrnS* also resembled those inferred in other invertebrates, including insects [58], crustaceans [5], miriapods [59] and nematodes [60]. We propose that in the future, new folding algorithms should first detect individual domains by comparing sequences with already known secondary structures using global or local alignments, and then fold each domain of target sequence using a known secondary structure as template. However, because the primary sequence of some domains diverged rapidly, e.g. domain I of *rrnS* gene, even between close related species, some motifs had to be folded again in Mfold [61], and in some cases even refined manually. Interestingly, suboptimal secondary structures of many motifs in the 16S and 12S rRNAs, i.e. with higher free energy, resembled more accurately the secondary structure previously described in other species, rather than the best folding with minimum free energy [62]. The secondary structures reported here are predictive and represent an attempt to provide the putative folding of rRNA-encoding genes that need to be validated in the future. Secondary structures and new folding algorithms should be based on large comparative studies of compensatory or semi-compensatory substitutions in paired regions and crystallographic studies to further validate the results that are largely beyond the scope of most mtDNA descriptions. Nonetheless, we think our secondary structures are a promising starting ground for future research, since the pairings involved in forming a tertiary structure are also conserved relative to those reported in other invertebrates (yellow lines in Figs. 5 and 6).

Most mitogenomes include a long non-coding region that works as control region and harbors the OR, as in, for instance, the amphipod genus *Metacrangonyx* [6]. Additionally, *Parachtes* and *Harpactocrates* mitogenomes possessed a second, shorter non-coding region, which has been suggested as the replication origin for the lagging strand in other species [63]. The length of the control region varies extensively across taxa, ranging from extremely short ones such as that in the crustacean amphipod *Metacrangonyx longipes* [64], which only harbors the OR, to up to 1 kb, as observed in other amphipods [6]. An interesting feature observed in the control region of the spiders examined here is a short duplicated region (less than 100 bp) with high nucleotide identity and few indels that resembles the putative origin of replication found in the short non-coding region located between *trnL2* and *trnN* (Fig. 7). The presence of this motif suggests a duplication or rearrangement event involving at least the control region in the most recent common ancestor of *Parachtes* and *Harpactocrates*, estimated at about 30 Mya [34]. The comparative analysis of the control region of several species of birds of the genus *Lanius* suggested that the control region originated by slipped-strand mispairing of a tandem repeat and its subsequent turnover [65]. The origin of replication is difficult to detect since its nucleotide sequence is not conserved even among close related species. However, it should share two main features: 1) a long stem-loop secondary structure usually with an adjacent poly-A or poly-T run, and 2) a TATA and GA(N) T motifs at the 5′ and 3′ ends of the stem-loop structure, although the motif may be extremely variable [6]. Those long stem-loop structures were found in the large control regions of all *Parachtes* and *Harpactocrates* mitogenomes examined, although it is difficult to ensure they are the correct OR since there are many similar secondary structures within the control regions and they are divergent across species. However, the high sequence identity of the short control region in *Parachtes* and *Harpactocrates* species with a short fragment within the large control region suggests that the fragment could be under evolutionary constraints and hence it could indeed act as an OR.

## Conclusions

The comparative analysis of the mitogenomes of closely related spiders in a diverse yet so far poorly studied lineage, allowed us to unravel that arm-less tRNAs may be a more predominant feature in the evolution of mitogenomes within some metazoan lineages than previously assumed. Arm-less tRNAs have been largely overlooked due to the algorithms with covarion models designed to discover tRNAs are based on cloverleaf-like structures. Based on our observation, we propose that novel automatic algorithms for the identification and folding of RNAs should include covariation models for armless tRNAs and multiple mispairing in the acceptor arm. However, the detection of arm-less tRNA genes using covarion models is extremely difficult since these short secondary structures cannot be differentiated from a random DNA fragments particularly in absence of close related species to be compared as is in our study. Finally, the use of species with well-dated divergence times has further enable us to hypothesize that the extreme reduction of encoded tRNA genes remained evolutionary stable for at least 30 million years, and confirms that the loss of DHU-arm in *trnS2* of the spiders of the suborder Opisthothelae is a synapormorphy for this taxonomic group. Nonetheless, the final tRNA molecules from those arm-less genes has to be confirmed by additional studies since post-transcriptional change may play an important role in transforming arm-less tRNA into fully functional tRNAs.

## Methods

Genomic DNA samples belonging to several species of the genus *Parachtes* and one representative of its sister genus *Harpactocrates*, both belonging to the spider family Dysderidae, were obtained from a previous study [34], namely *P. riberai* (Spain, Balearic Islands, Mallorca, Lluc; code LB105), *P. teruelis* (Spain, Iberian Peninsula, Castilla-León, Guadalajara, Sigüenza; code LB103), *P. romandiolae* (Italy, Toscana, Firenze, Vallombrosa; code K352), *P. limbarae* (Italy, Sardinia, Sassari, Monte Limbara; code K475), *P. ignavus* (France, Corsica, Region d’Ajaccio, Foce di Vizzabona; code K479), and *Harpactocrates apennicola* (Italy, Toscana, Massa Carrara, Passo del Cerreto, code K350). Geographic coordinates, DNA extraction, and purification methods of the vouchers are detailed in the former study [34]. Sequences from 32 additional spider mitogenomes were downloaded from the NCBI Genome-Organelles database, as in November 2017.

Mitogenomes were amplified as two overlapping long PCR fragments using species-specific primers (Additional file 6: Table S2; Additional file 7: Figure S5) designed on short mitochondrial sequences obtained in a previous study [34]. Long PCR protocol followed the method described elsewhere [6] with specific annealing temperature for each primer set (52–64 °C). Primers were designed to amplify a long fragment of about 9 kb comprising the mitochondrial region between *cox1* and *rrnL* (5′ to 3′), and a shorter one of about 5 kb comprising the remaining circular genome from 5′ *rrnL* to 3′ *cox1*. Polymerization of the shorter fragment was only successful if annealing temperature decreased from the optimal 68 °C to 62–60 °C probably due to high A + T richness of control region [7]. For some species, the amplification of PCR products was scarce or included secondary bands that would reduce the yield of direct sequencing. Therefore, the candidate band to be sequenced was excised from agarose gel to be re-amplified by PCR. Since UV light nicks DNA, which consequently inhibits DNA polymerization, particularly in long DNA fragments, agarose gel and DNA were stained with crystal violet. This method allows the direct observation and excision of DNA bands under regular light avoiding any DNA damage [66]. Once recovered, DNA was purified in silica columns (QIAquick PCR Purification Kit, QIAGEN, Hilden, Germany) and successfully re-amplified by increasing annealing temperature of two degrees.

The sequence of the mitogenome of the species *Parachtes riberai* was attained by following the strategy described elsewhere [7, 64]. Briefly, DNA fragments produced by long PCR amplification were digested by individual restriction enzymes (*Alu* I, *Dra* I, *Rsa* I and *Taq* I), and then DNA fragments were pooled, purified and cloned. Finally, clones were sequenced using the Sanger protocol and mitochondrial regions with no coverage were extended by primer walking (available upon request) using initial long PCR fragments as template. The mitogenomes of the remaining five species were obtained by constructing libraries from long PCR fragments and subsequently pyrosequecing them using Roche FLX/454 with a unique tag for each species-specific library.

Mitogenomes were annotated in the new MITOS2 version of Mitoswebserver [67] available at http://mitos2.bioinf.uni-leipzig.de/index.py. Many tRNA genes were not identified by Infernal predictions as implemented in the MiTFi algorithm in MITOS2 or by tRNAscan-SE v1.3.1 [68] using the complete mitochondrial sequences as template. Hence, we used sequences between protein coding regions as template allowing an overlapping of 50 bp between genes and a low e-value of 100 for Infernal fast mode to detect tRNAs without arms and with several mismatches in the acceptor arm. The candidate tRNA sequences obtained from the six species using this approach were aligned and secondary structures compared in order to obtain a common optimal and conserved structure for each tRNA across the six species. If DHU or TΨC arms were not conserved across the six species then the secondary structure of an alternative arm-less tRNAs was obtained manually by finding the best complementary sequences forming the acceptor arm. The correct annotation of ribosomal genes *rrnL* and *rrnS* was corroborated by aligning the ribosomal sequences of the six species along with the sequences of the 12 spider mitogenomes available in MetAMiGA [52]. Secondary structure of ribosomal genes *rrnL* and *rrnS* from MITOS2 were inaccurate since motives from different domains were joined together. Therefore, short motifs of each domain were manually revised using both nucleotide alignments and previously known secondary structures as references. Secondary structures of short motifs were predicted in Mfold v3.6 [61] allowing suboptimal structures that were up to 20% above the estimated minimum free energy. Ribosomal structures were based on the models proposed for the branchipods *Artemia salina* Linnaeus, 1758 and *Artemia franciscana* Kellogg, 1906, the amphipods *Metacrangonyx “bovei”* [6], and the amphipod *Pseudoniphargus sorbasiensis* Notenboom, 1987 [5], the ascalaphid owlfly *Libelloides macaronius* Scopoli, 1763 [58], and the nematode *Caenorhabditis elegans* Maupas, 1900 [60]. Since the reconstruction of the secondary structure of rRNAs 16S and 12S was laborious, we only performed the accurate reconstruction in the species *P. romandiolae*. DNA sequences were aligned in MAFFT v7.273 [69]. Secondary structures were visualized and modified in VARNA v3.9 [70]. Ancestral gene orders at the inner nodes were estimated in the software TreeREx v.1.85 [71] using the strong consistency method and enforcing the tree topology including the main spider lineages (Fig. 1). The algorithm implemented in TreeREx allows to estimate the molecular mechanisms involved in the gene rearrangements such as transpositions and inversions. Polytomies were removed by trimming terminals since species involved shared identical gene orders. The sequences of *Tetragnatha nitens* Audouin, 1826 (Araneoidea, Tetragnathidae) and *Agelena silvatica* Oliger, 1983 (Agelenidae), and *Carrhotus xanthogramma* Latreille, 1819 (Dionycha, Salticidae) were not included in the analyses since they show complex gene rearrangement patterns. Finally, nucleotide and amino acid composition and codon usage profiles (RSCU) were estimated in MEGA v5.10 [72], and AT and GC skews with the formula ATskew = (A-T)/(A + T) and GCskew = (G-C)/(G + C), as proposed by [73].

## Additional files

Additional file 1: **Figure S1.** A + T composition and AT and GC skews for the 6 mitogenomes reported for *Parachtes* and *Harpactocrates*. The same features are shown for each protein-coding gene and pooling by codon position and coding strand. (PDF 39 kb)

Additional file 2: **Figure S2.** Pie plot showing amino acid frequencies for each species studied here: *Parachtes teruelis* (1), *P. riberai* (2), *Harpactocrates apennicola* (3), *P. romandiolae* (4), *P. limbarae* (5), and *P. ignavus* (6). Arrows denote those amino acid which frequencies changed greatly depending of coding strand. (PDF 385 kb)

Additional file 3: **Figure S3.** Secondary structures predicted by Infernal-MiTFi (MITOS2) for the 22 tRNAs for the six spider species: *Parachtes teruelis* (mitos103), *P. riberai* (mitos105), *P. romandiolae* (mitos352), *P. limbarae* (mitos475), *P. ignavus* (mitos479), and *Harpactocrates apennicola* (mitos350). (PDF 397 kb)

Additional file 4: **Figure S4.** Modified secondary structures of tRNA from those foldings predicted by mitfi (mitos2) for the 22 tRNAs for the six spider species: *Parachtes teruelis* (sp103), *P. riberai* (sp105), *P. romandiolae* (sp352), *P. limbarae* (sp475), *P. ignavus* (sp479), and *Harpactocrates apennicola* (sp350). Some tRNAs show an alternative folding either as three arms tRNA or as arm-less tRNAs. (PDF 1550 kb)

Additional file 5: **Table S1.** Gene orders found in the 32 mitogenomes available in GenBank and MetAMiGA, that retrieved from *Parachtes* and *Harpactocrates* plus the ancestral patterns for Pancrustanea, Arthropoda and Chelicetata . (XLSX 13 kb)

Additional file 6: **Table S2.** Primer list used to amplify long fragments 5′ cox1–3′ rrnL (9,5 kb). *Parachtes teruelis* (sp103), *P. riberai* (sp105), *P. romandiolae* (sp352), *P. limbarae* (sp475), *P. ignavus* (sp479), and *Harpactocrates apennicola* (sp350). Note that reverse primer was identical for all species except for *P. riberai* (sp105). (DOC 13 kb)

Additional file 7: **Figure S5.** Photography of long PCR fragments analyzed by agaroge gel electrophoresis after ethidium bromide staining and UV exposition. *Parachtes teruelis* (103), *P. riberai* (105), *P. romandiolae* (352), *P. limbarae* (475), *P. ignavus* (479), and *Harpactocrates apennicola* (350). S refers to short PCR fragment and L to large one. (TIF 981 kb)

## Data Availability

The mitogenome sequences are available on GenBank under the accession numbers: *Parachtes riberai* MN052919, *Parachtes ignavus* MN052920, *Parachtes teruelis* MN052921, *Parachtes limbarae* MN052922, *Parachtes romandiolae* MN052923, and *Harpactocrates apennicola* MN052924.

## Abbreviations

*atp6* and *atp8*: Genes for ATP synthase subunits 6 and 8
bp: Base pair
*cob*: Gene for cytochrome b
*cox1*, *cox2* and cox3: Genes for cytochrome c oxidase subunits 1, 2 and 3
CR: Control region
DNA: Deoxyribonucleic acid
kb: Kilo base pair
*nad1*, *nad2*, *nad3*, *nad4*, *nad4L*, *nad5* and *nad6*: Mitochondrial genes for NADH dehydrogenase subunits 1–6 and 4 L
OR: origin of replication
PCGs: Protein-coding genes
PCR: Polymerase chain reaction
rRNA: Ribosomal RNA
rrnS and rrnL: Genes for small (12S) and large (16S) subunits of ribosomal RNA
RSCU: Relative Synonymous Codon Usage
*single letter*: tRNA for different amino-acids A (alanine), C (cysteine), D (aspartic acid), E (glutamic acid), F (phenylalanine), G (glycine), H (histidine), I (isoleucine), K (lysine), L1 (leucine anticodon NAG), L2 (leucine anticodon YAA), M (methionine), N (asparagine), P (proline), Q (glutamine), R (arginine), S1 (serine anticodon NCU), S2 (serine anticodon NGA), T (threonine), V (valine), W (tryptophan), Y (tyrosine)
*trn*: gene for transfer RNA (*trnA*, *trnC*, *trnD*, *trnE*, *trnF*, *trnG*, *trnH*, *trnI*, *trnK*, *trnL1*, *trnL2*, *trnM*, *trnN*, *trnP*, *trnQ*, *trnR*, *trnS1*, *trnS2*, *trnT*, *trnU*, *trnV*, *trnW*, *trnY*)
tRNA: Transfer RNA
