# Supplementary figures and images for: Arm-less mitochondrial tRNAs conserved for over 30 millions of years in spiders
Source: BMC Genomics. 2019 Aug 23;20:665. doi: 10.1186/s12864-019-6026-1 (PMC6706885; doi:10.1186/s12864-019-6026-1)

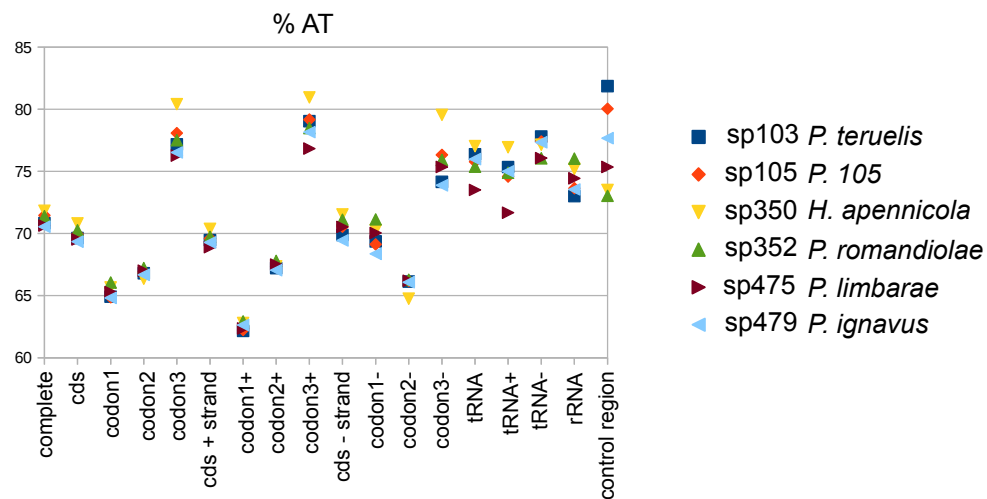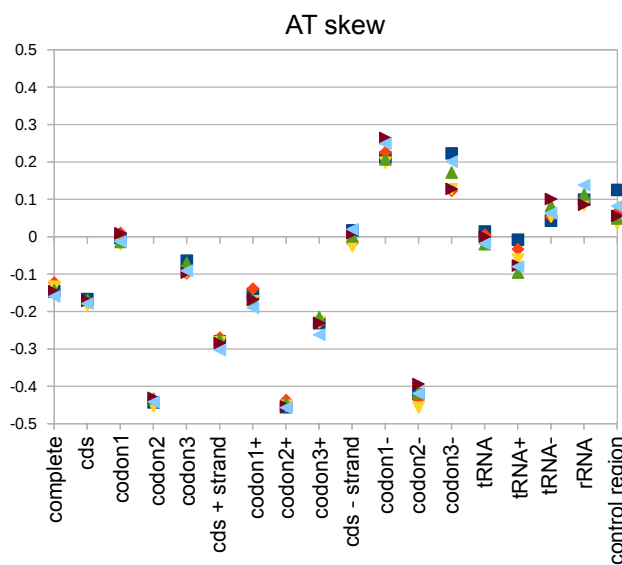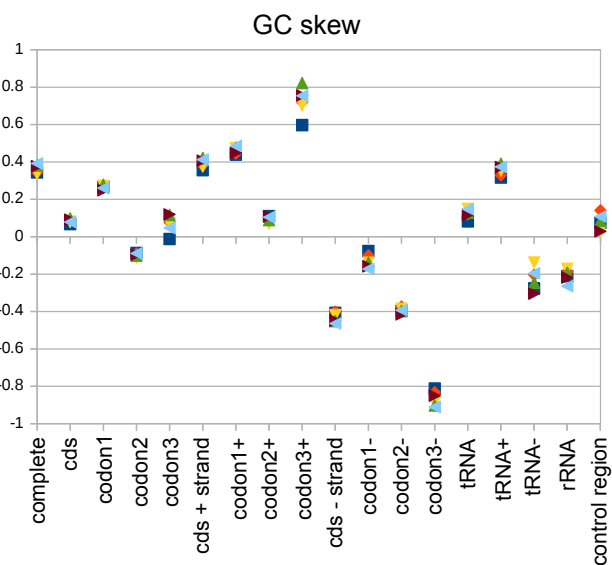

Supplement: Supplementary file 1 — Figure S1. A + T composition and AT and GC skews for the 6 mitogenomes reported for Parachtes and Harpactocrates. The same features are shown for each protein-coding gene and pooling by codon position and coding strand. (PDF 39 kb) [file 12864_2019_6026_MOESM1_ESM.pdf]

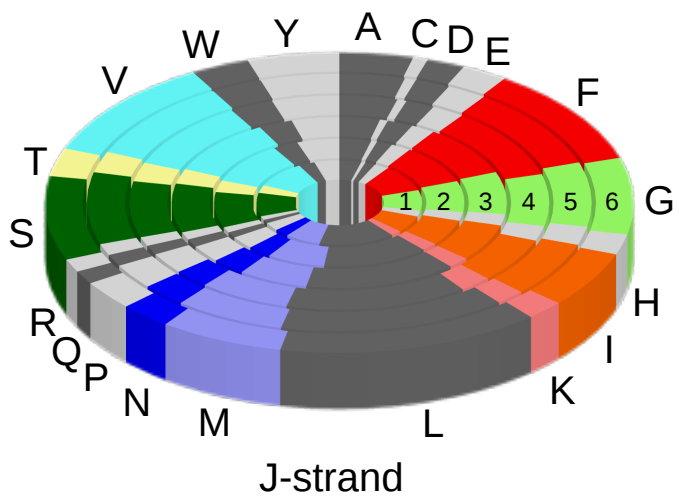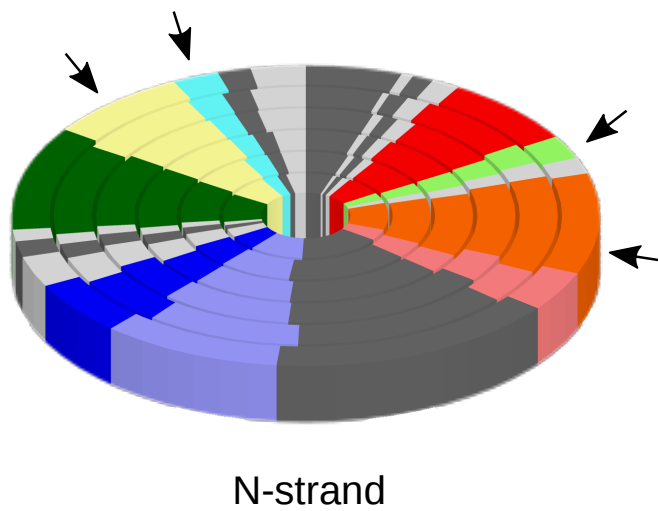

Supplement: Supplementary file 2 — Figure S2. Pie plot showing amino acid frequencies for each species studied here: Parachtes teruelis (1), P. riberai (2), Harpactocrates apennicola (3), P. romandiolae (4), P. limbarae (5), and P. ignavus (6). Arrows denote those amino acid which frequencies changed greatly depending of coding strand. (PDF 385 kb) [file 12864_2019_6026_MOESM2_ESM.pdf]

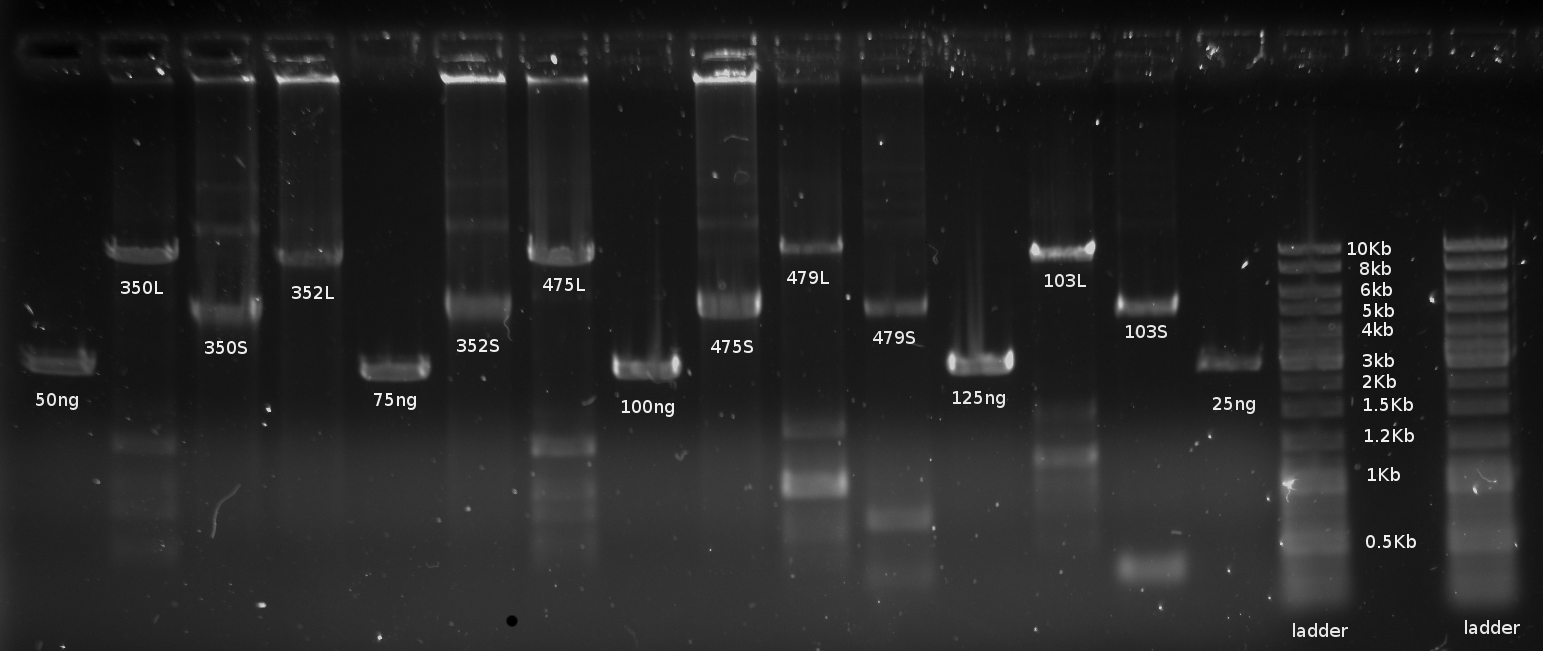

Supplement: Supplementary file 7 — Figure S5. Photography of long PCR fragments analyzed by agaroge gel electrophoresis after ethidium bromide staining and UV exposition. Parachtes teruelis (103), P. riberai (105), P. romandiolae (352), P. limbarae (475), P. ignavus (479), and Harpactocrates apennicola (350). S refers to short PCR fragment and L to large one. (TIF 981 kb) [file 12864_2019_6026_MOESM7_ESM.tif]
