# Supplementary material for: Arm-less mitochondrial tRNAs conserved for over 30 millions of years in spiders
Source: BMC Genomics. 2019 Aug 23;20:665. doi: 10.1186/s12864-019-6026-1 (PMC6706885; doi:10.1186/s12864-019-6026-1)
Supplement: Supplementary file 3 — Figure S3. Secondary structures predicted by Infernal-MiTFi (MITOS2) for the 22 tRNAs for the six spider species: Parachtes teruelis (mitos103), P. riberai (mitos105), P. romandiolae (mitos352), P. limbarae (mitos475), P. ignavus (mitos479), and Harpactocrates apennicola (mitos350). (PDF 397 kb) [file 12864_2019_6026_MOESM3_ESM.pdf]

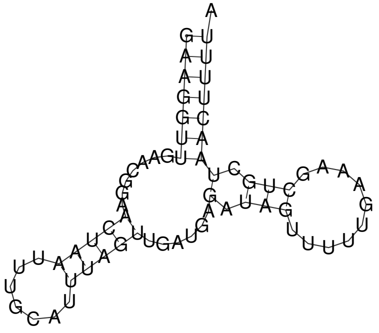

mitos103

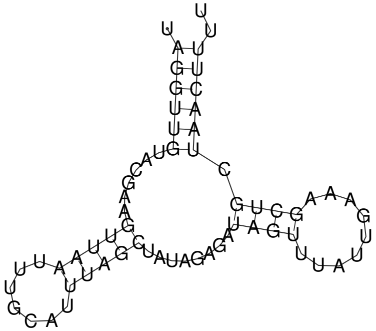

mitos105

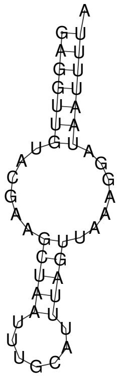

mitos352

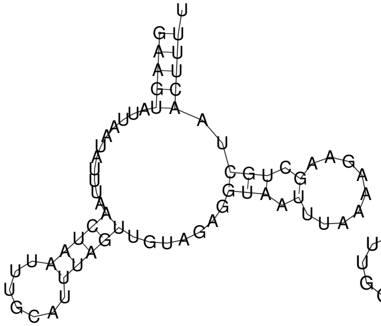

mtitos350

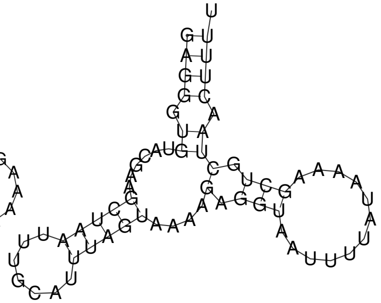

mitos475

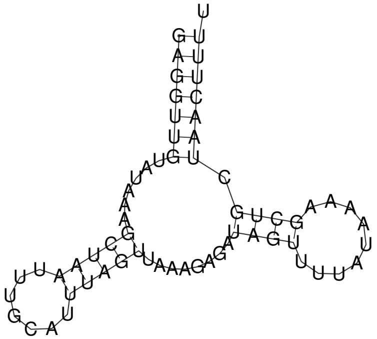

mitos479

tRNA_A

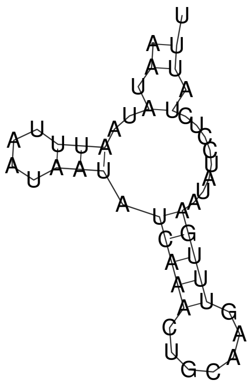

mitos103

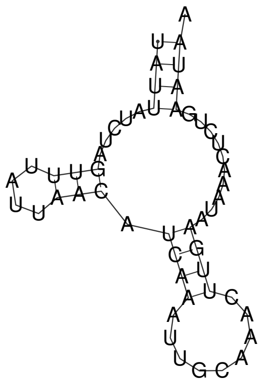

mitos105

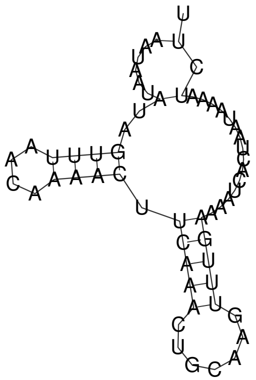

mitos350

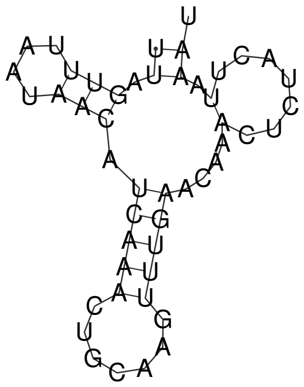

mitos352

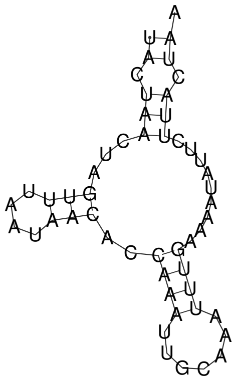

mitos475

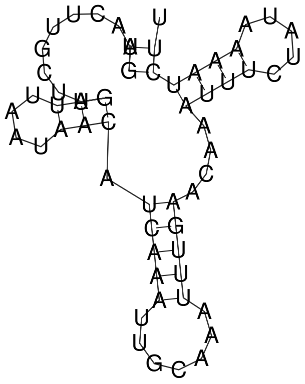

mitos479

tRNA_C

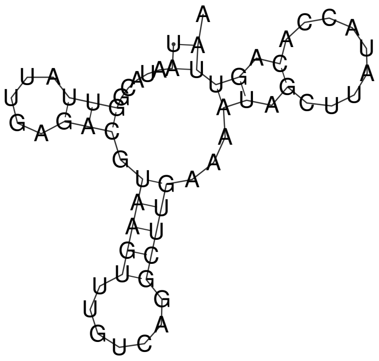

mitos103

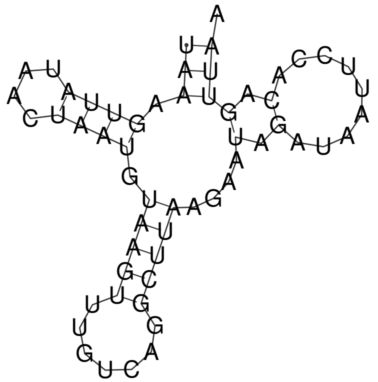

mitos105

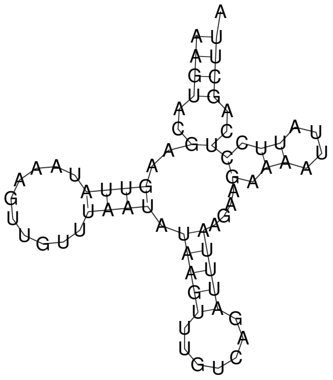

mitos350

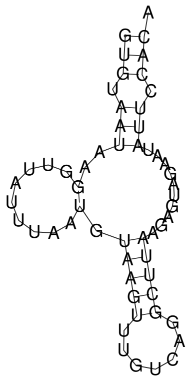

mitos352

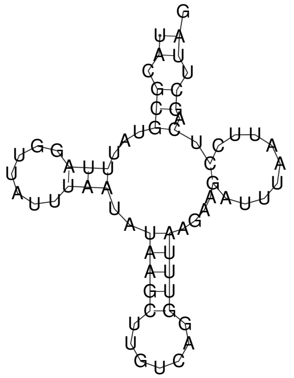

mitos475

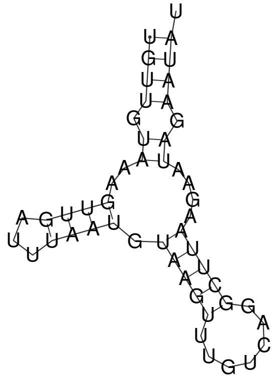

mitos479

tRNA_D

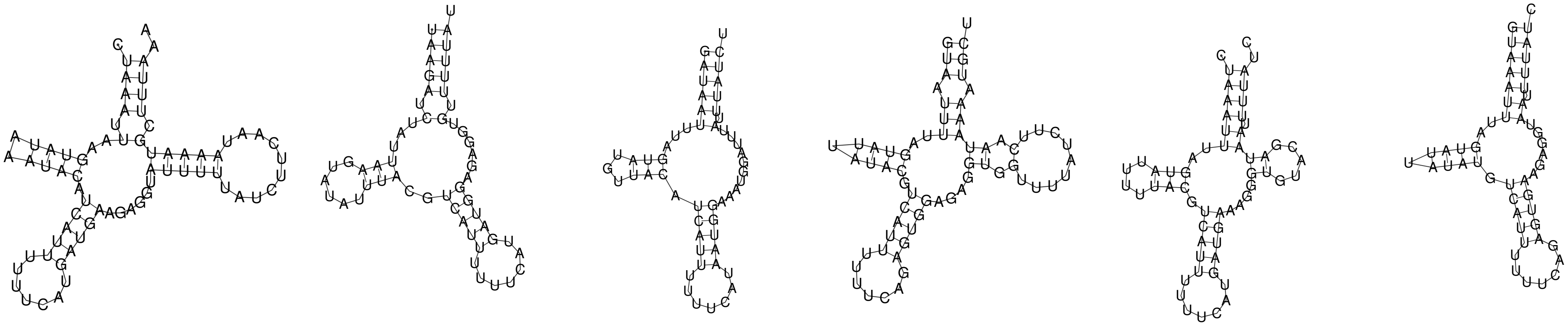

mitos103 mitos105 mitos350 mitos352 mitos475 mitos479

tRNA_E

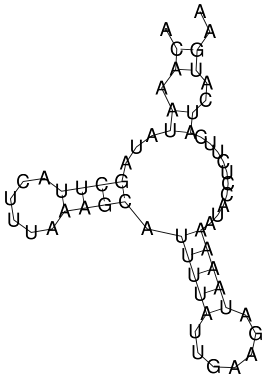

mito103

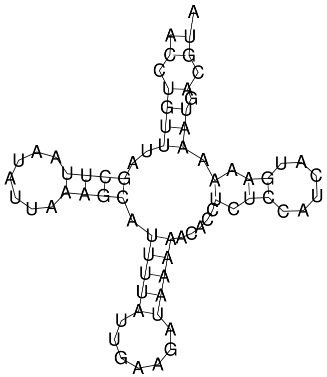

mitos105

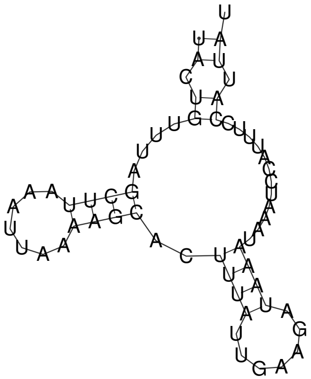

mitos350

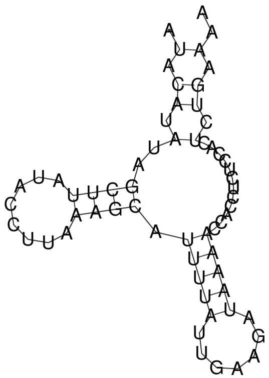

mitos352

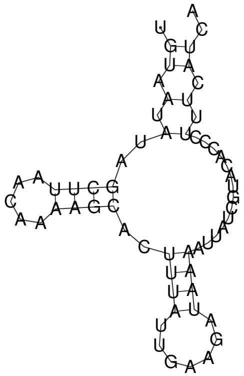

mitos475

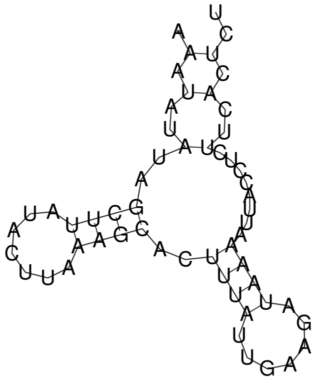

mitos479

tRNA_F

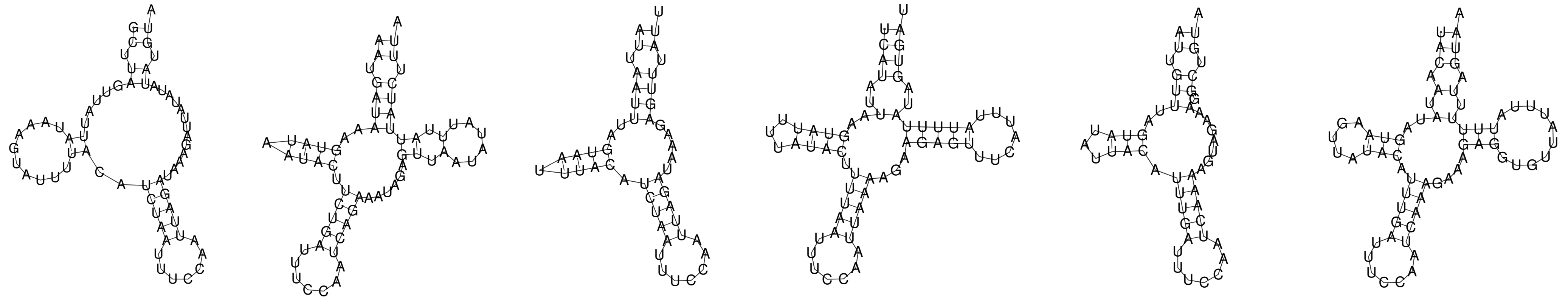

mitos103 mitos105 mitos350 mitos352 mitos475 mitos479

tRNA_G

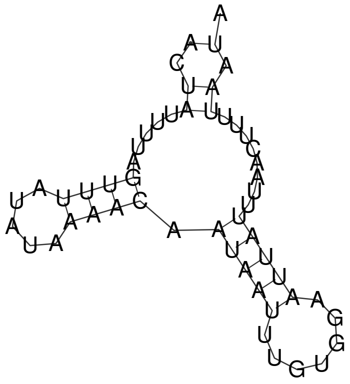

mitos103

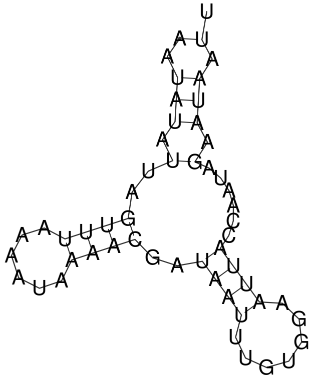

mitos105

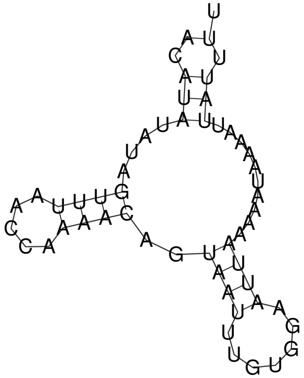

mitos350

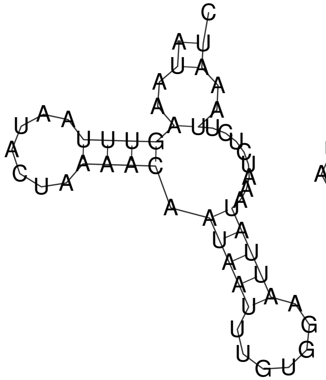

mitos352

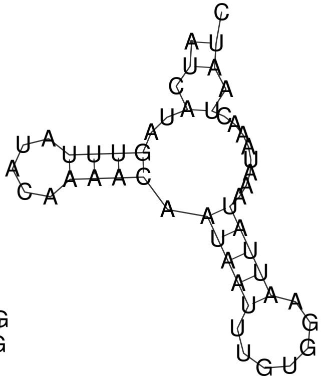

mito475

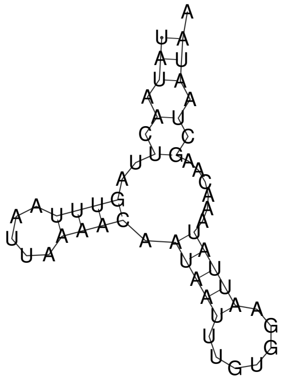

mitos479

tRNA_H

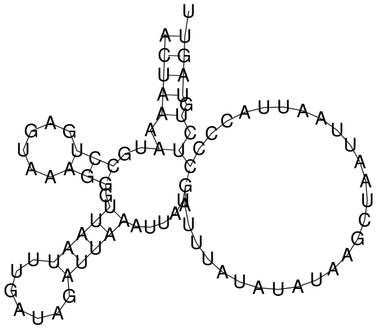

mitos103

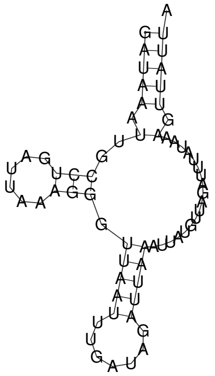

mitos105

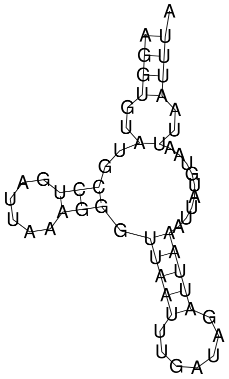

mitos350

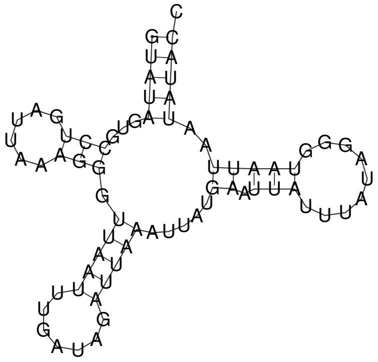

mitos352

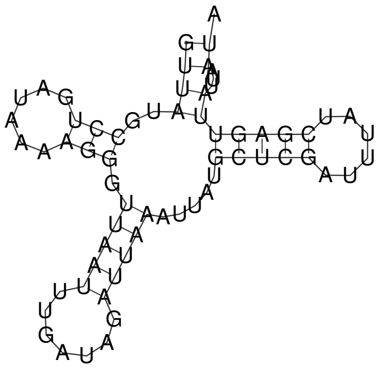

mitos475

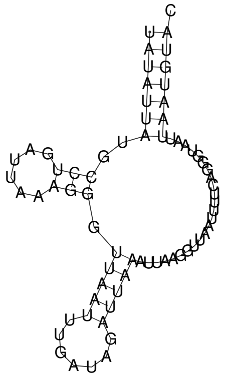

mitos479

tRNA_I

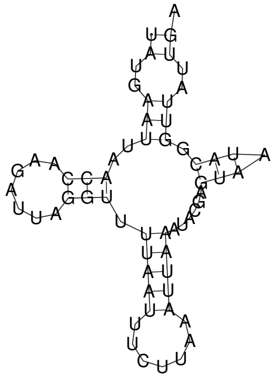

mitos103

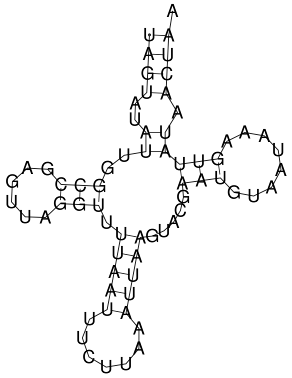

mitos105

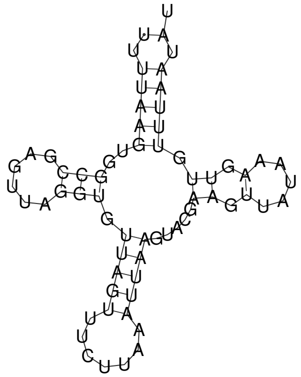

mitos320

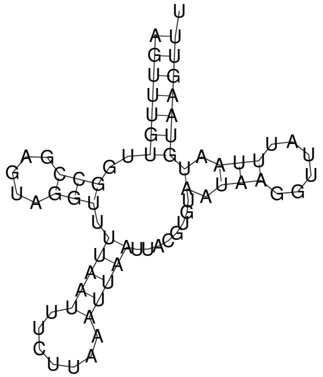

mitos352

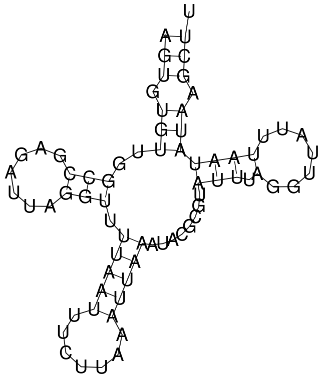

mitos475

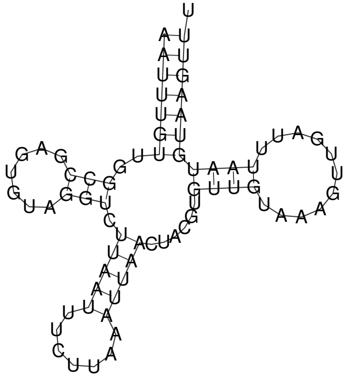

mitos479

tRNA_K

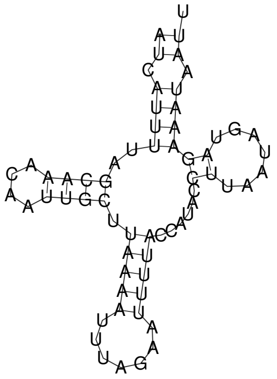

mitos103

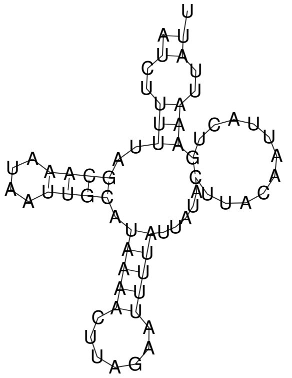

mitos105

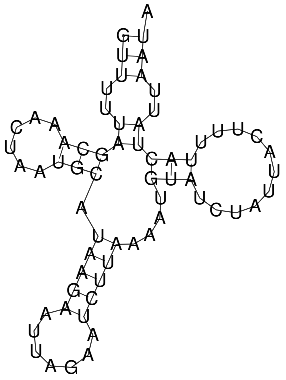

mitos350

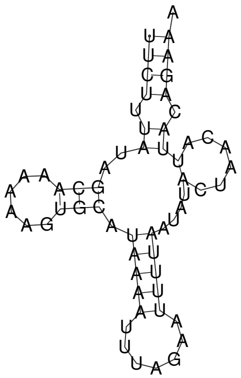

mitos352

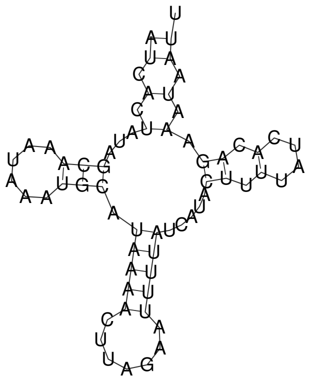

mitos475

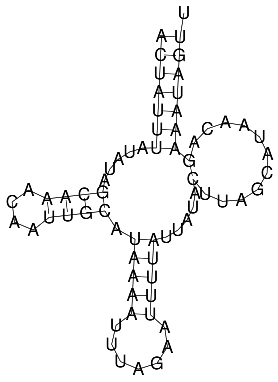

mitos479

tRNA_L1

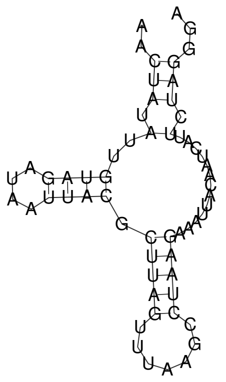

mitos103

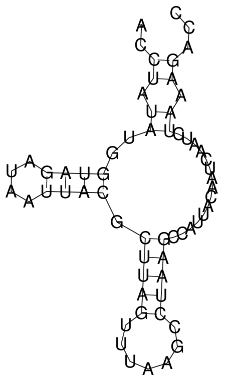

mitos105

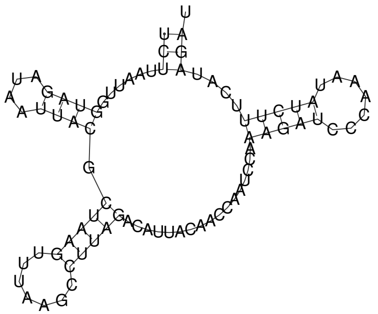

mitos350

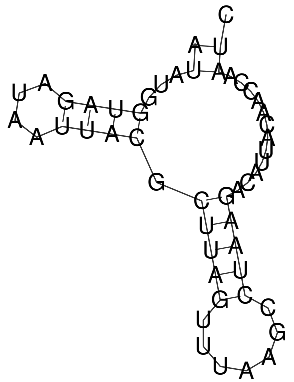

mitos352

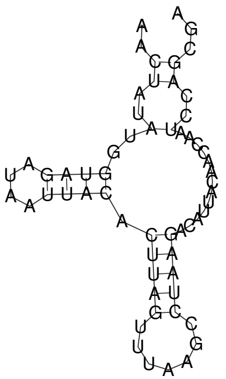

mitos475

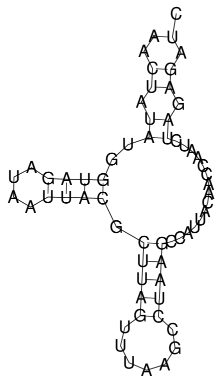

mitos479

tRNA_L2

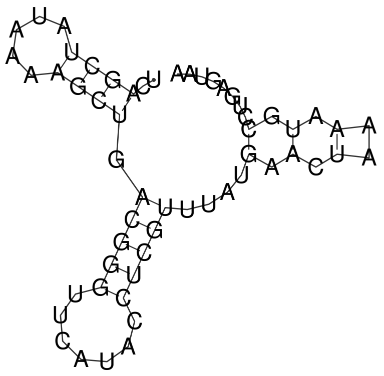

mitos103

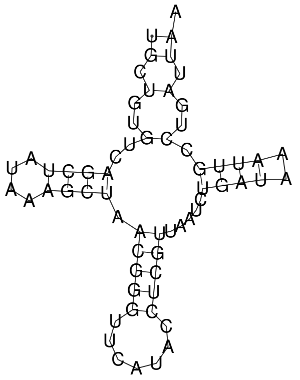

mitos105

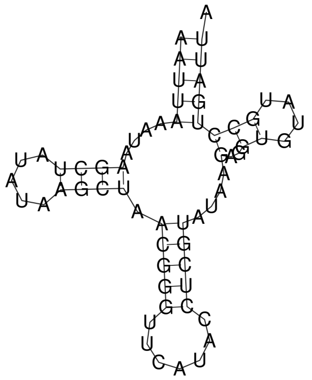

mitos350

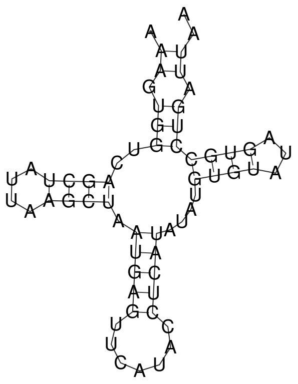

mitos352

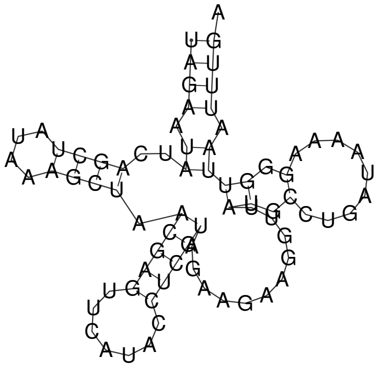

mitos475

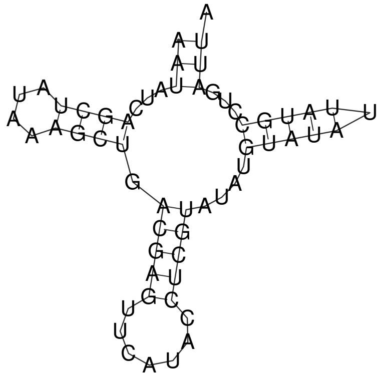

mitos479

tRNA_M

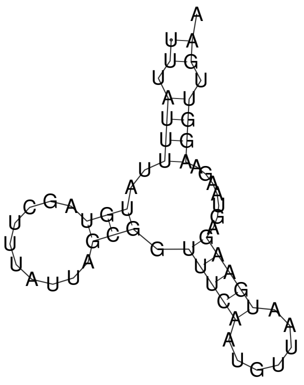

mitos103

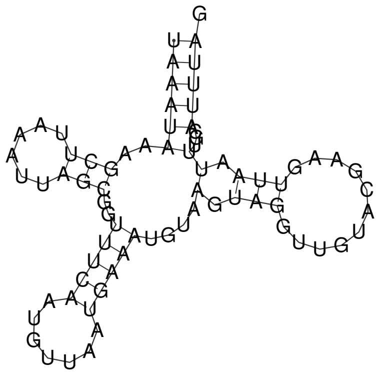

mitos105

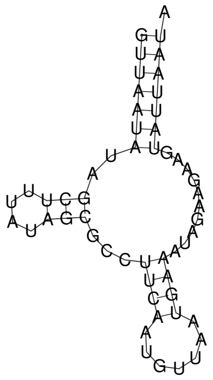

mitos350

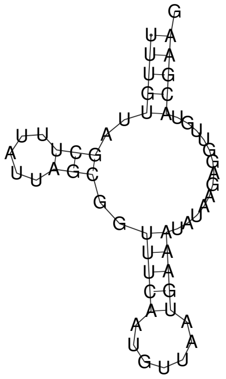

mitos352

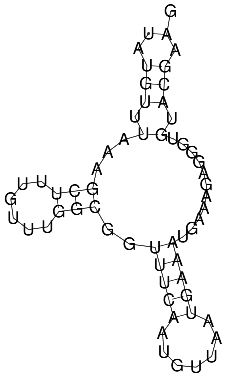

mitos475

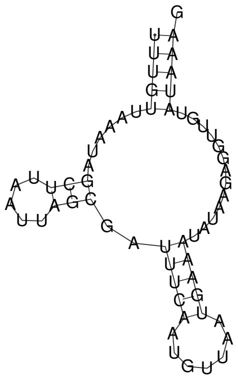

mitos479

tRNA_N

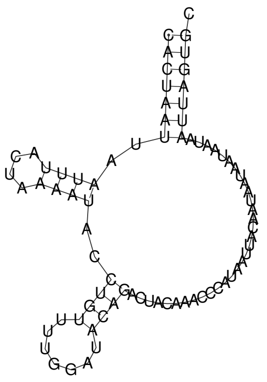

mitos103

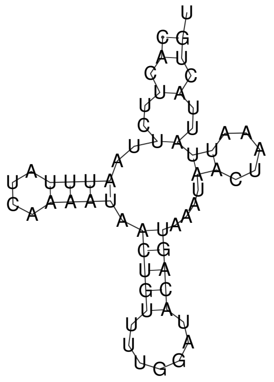

mitos105

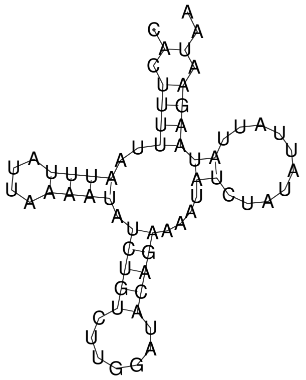

mitos350

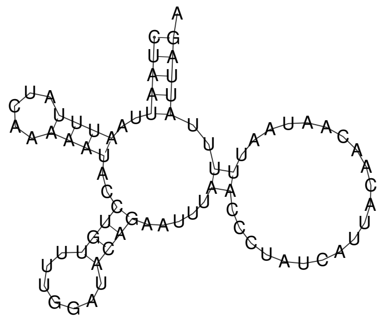

mmitos352

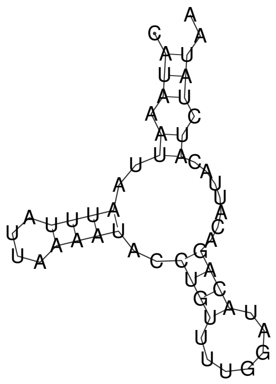

mitos475

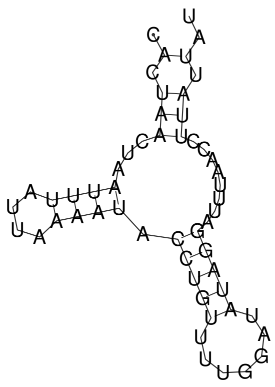

mitos479

tRNA_P

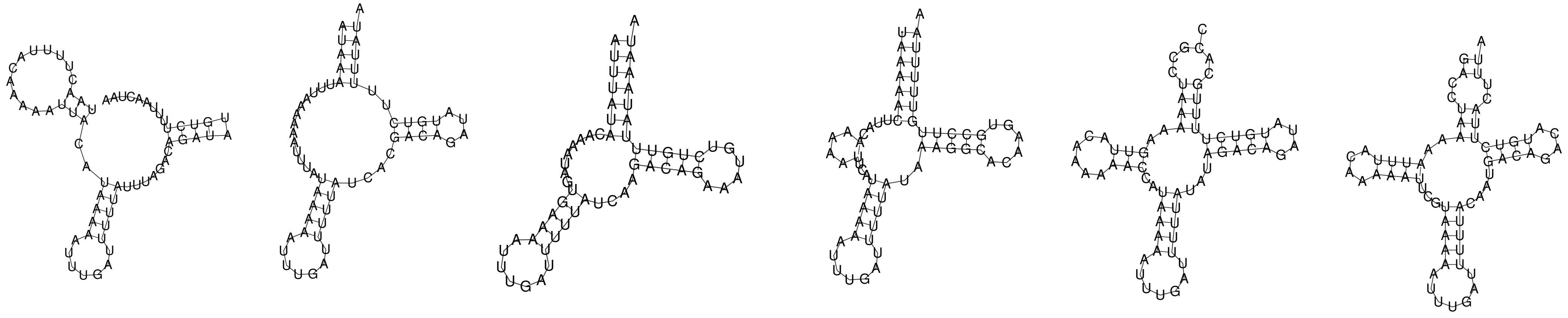

mitos103 mitos105 mitos350 mitos352 mitos475 mitos479

tRNA_Q

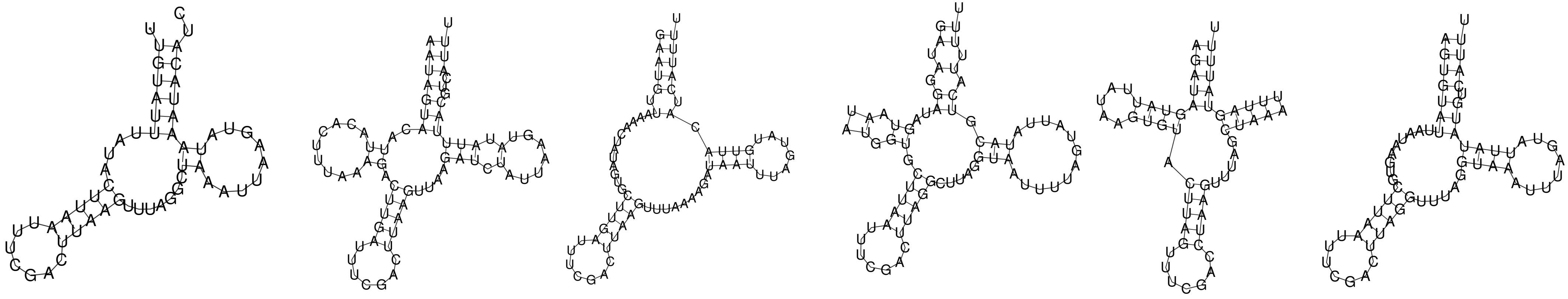

mitos103 mitos105 mitos350 mitos352 mitos475 mitos479

tRNA_R

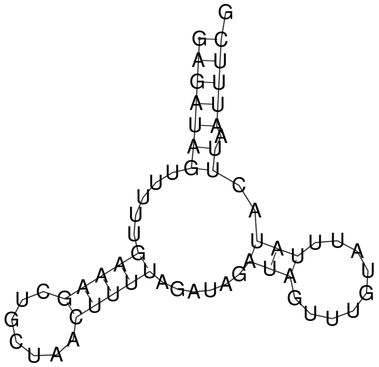

mitos103

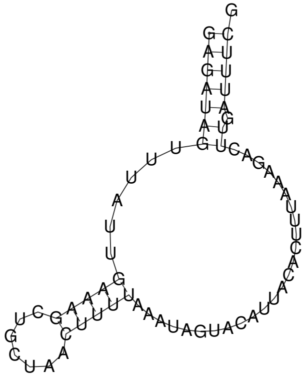

mitos105

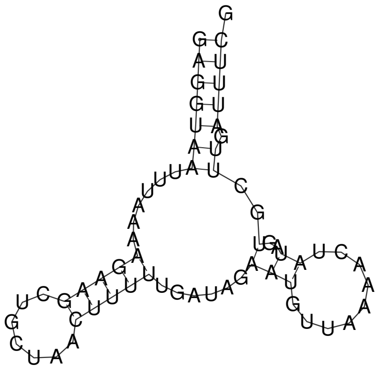

mitos350

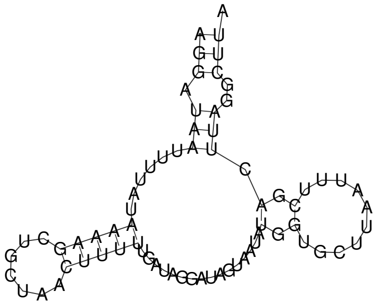

mitos352

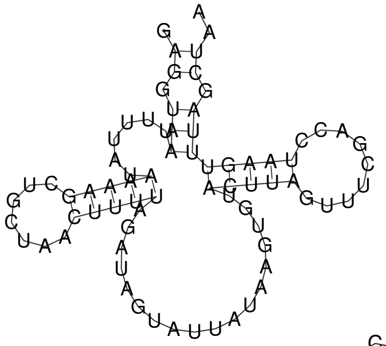

mitos475

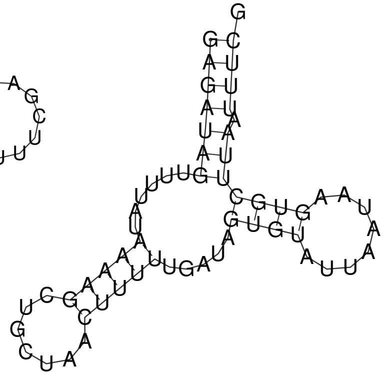

mitos479

tRNA_S1

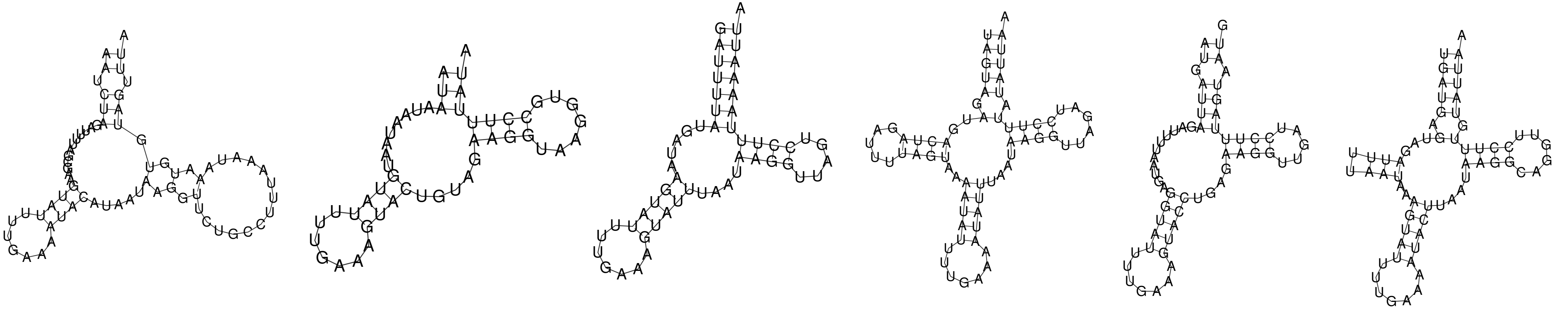

mito103 mitos105 mitos350 miitos352 mitos475 mitod479

tRNA_S2

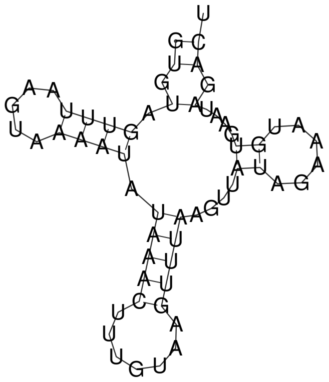

mitos103

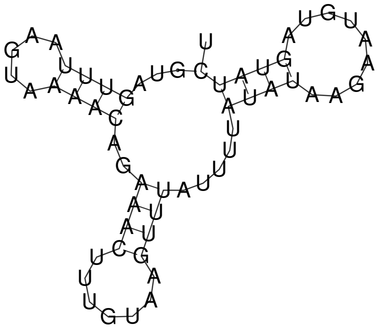

mitos105

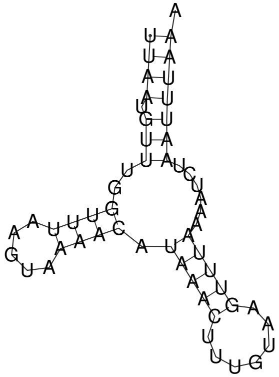

mitos350

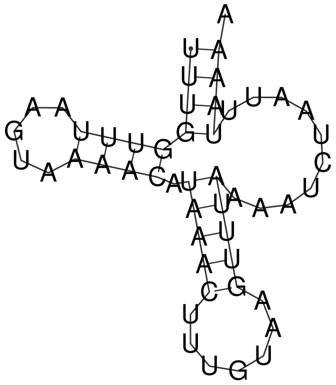

mitos352

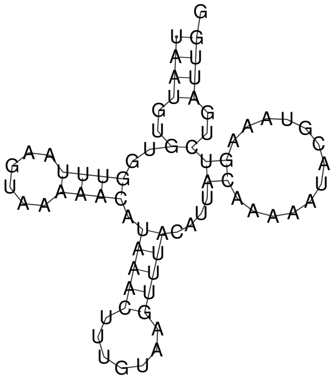

mitos475

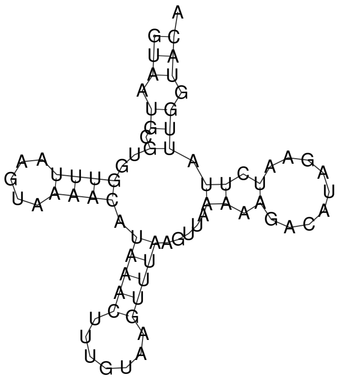

mito479

tRNA_T

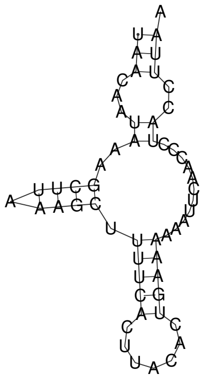

mitos103

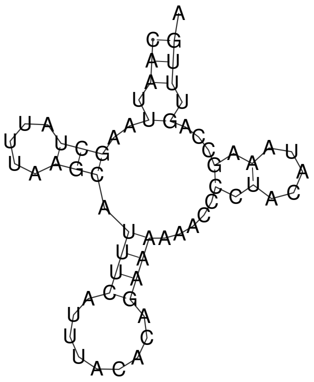

mitos105

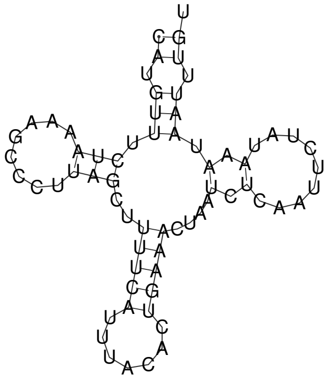

mitos350

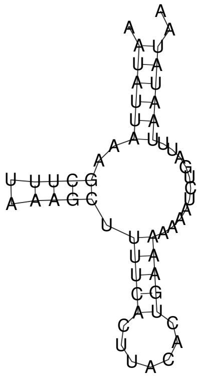

mitos352

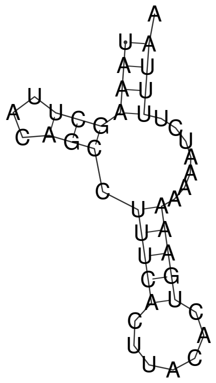

mitos475

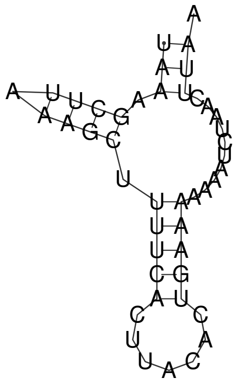

mitos479

tRNA_V

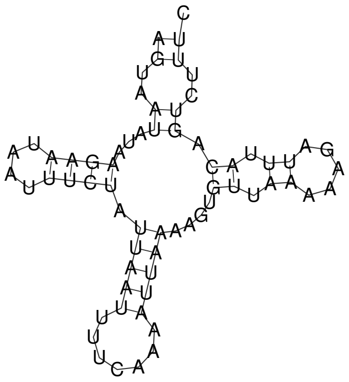

mitos103

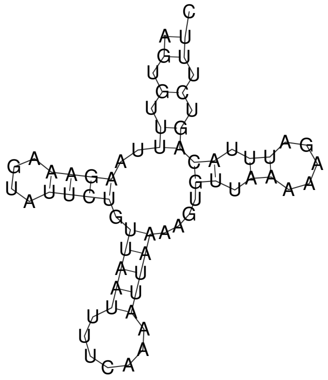

mitos105

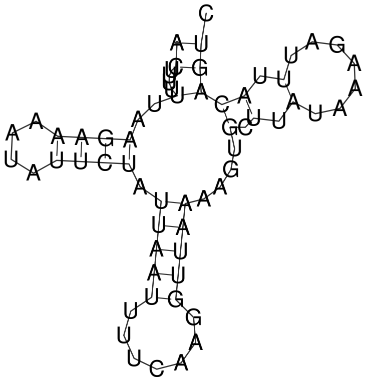

mitos350

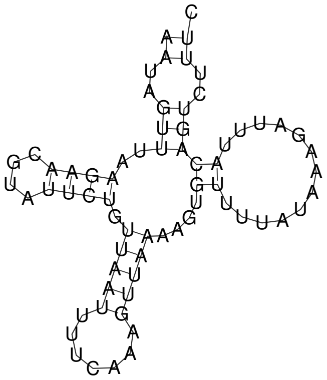

mitos352

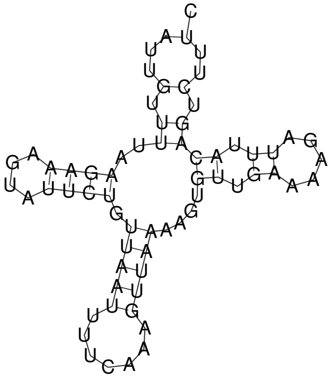

mitos475

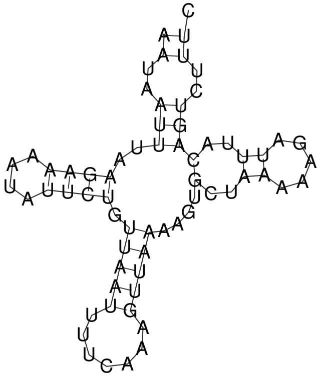

mitos479

tRNA_W

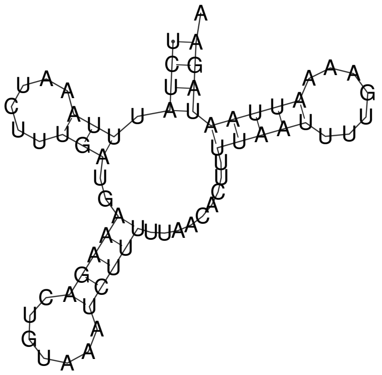

mitos103

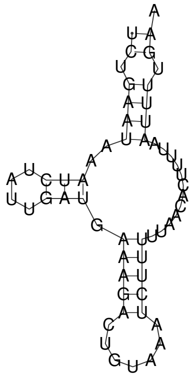

mitos105

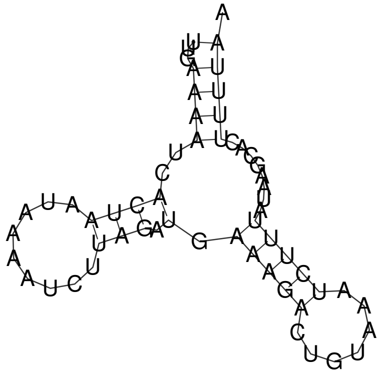

mitos350

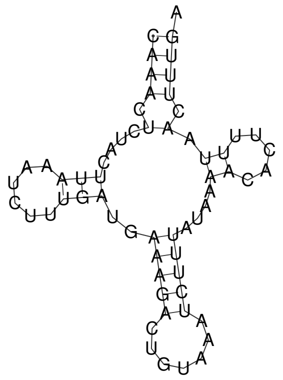

mitos352

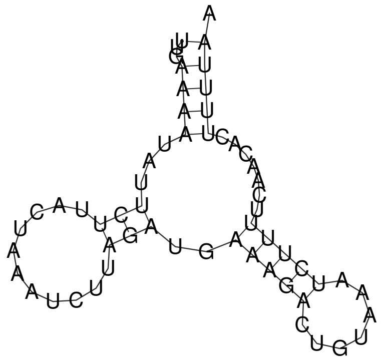

mitos475

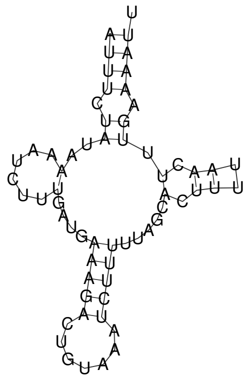

mitos479

tRNA_Y
