# Supplementary material for: Arm-less mitochondrial tRNAs conserved for over 30 millions of years in spiders
Source: BMC Genomics. 2019 Aug 23;20:665. doi: 10.1186/s12864-019-6026-1 (PMC6706885; doi:10.1186/s12864-019-6026-1)
Supplement: Supplementary file 4 — Figure S4. Modified secondary structures of tRNA from those foldings predicted by mitfi (mitos2) for the 22 tRNAs for the six spider species: Parachtes teruelis (sp103), P. riberai (sp105), P. romandiolae (sp352), P. limbarae (sp475), P. ignavus (sp479), and Harpactocrates apennicola (sp350). Some tRNAs show an alternative folding either as three arms tRNA or as arm-less tRNAs. (PDF 1550 kb) [file 12864_2019_6026_MOESM4_ESM.pdf]

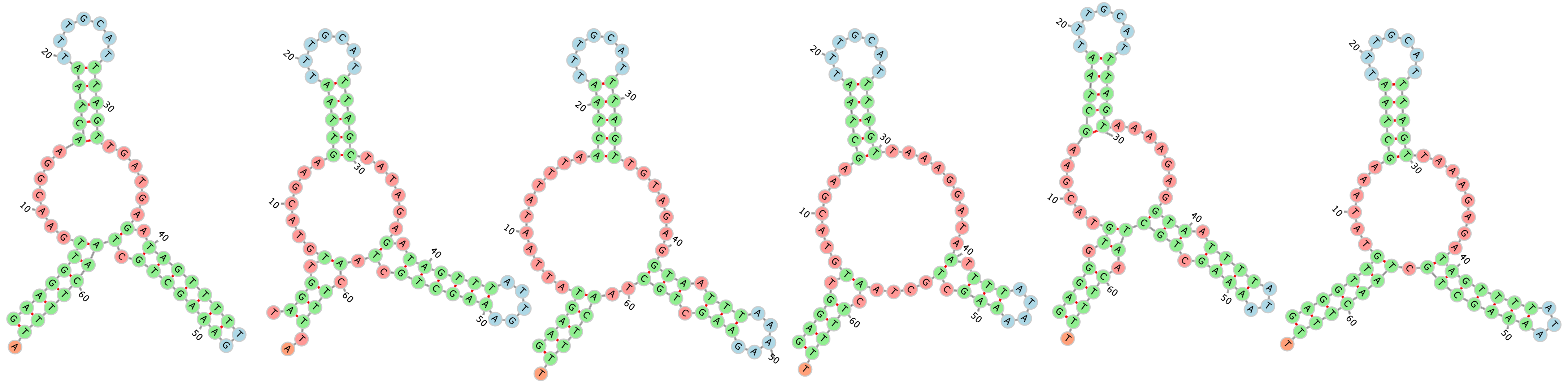

trnA

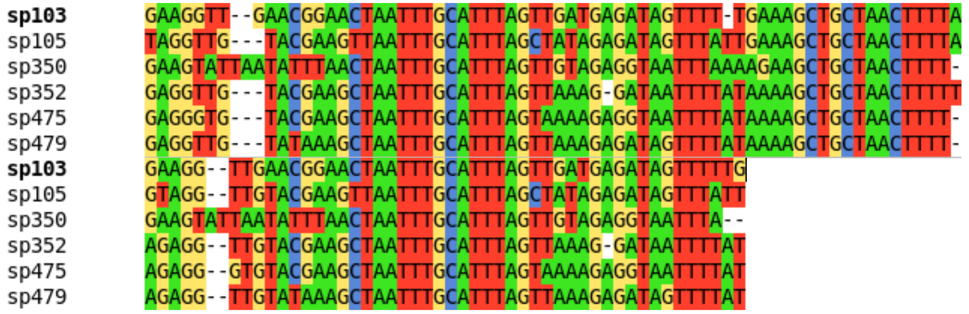
sp103 GAAGGTT--GAACGGAACTAATTTGCATTTAGTTGATGAGATAGTTTT-TGAAAGCTGCTAACTTTTA
sp105 TAGGTTG---TACGAAGTTAATTTGCATTTAGCTATAGAGATAGTTTATTGAAAGCTGCTAACTTTTA
sp350 GAAGTATTAATATTTAACTAATTTGCATTTAGTTGTAGAGGTAATTTAAAAGAAGCTGCTAACTTTT-
sp352 GAGGTTG---TACGAAGCTAATTTGCATTTAGTTAAAG-GATAATTTTATAAAAGCTGCTAACTTTTT
sp475 GAGGGTG---TACGAAGCTAATTTGCATTTAGTAAAAGAGGTAATTTTATAAAAGCTGCTAACTTTT-
sp479 GAGGTTG---TATAAAGCTAATTTGCATTTAGTTAAAGAGATAGTTTTATAAAAGCTGCTAACTTTT-
sp103 GAAGG--TTGAACGGAACTAATTTGCATTTAGTTGATGAGATAGTTTTTG
sp105 GTAGG--TTGTACGAAGTTAATTTGCATTTAGCTATAGAGATAGTTTATT
sp350 GAAGTATTAATATTTAACTAATTTGCATTTAGTTGTAGAGGTAATTTA--
sp352 AGAGG--TTGTACGAAGCTAATTTGCATTTAGTTAAAG-GATAATTTTAT
sp475 AGAGG--GTGTACGAAGCTAATTTGCATTTAGTAAAAGAGGTAATTTTAT
sp479 AGAGG--TTGTATAAAGCTAATTTGCATTTAGTTAAAGAGATAGTTTTAT

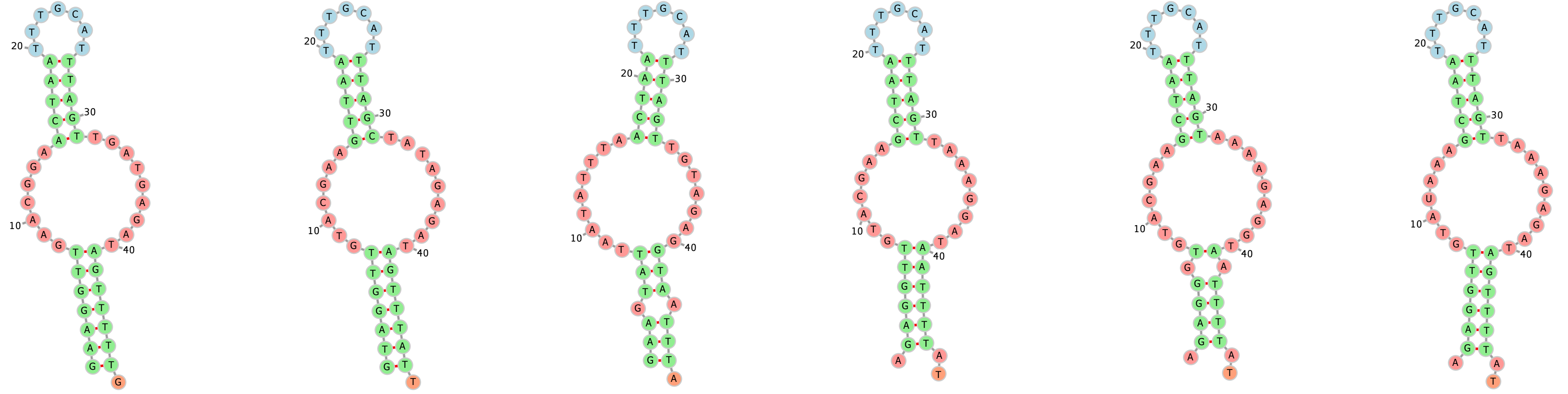

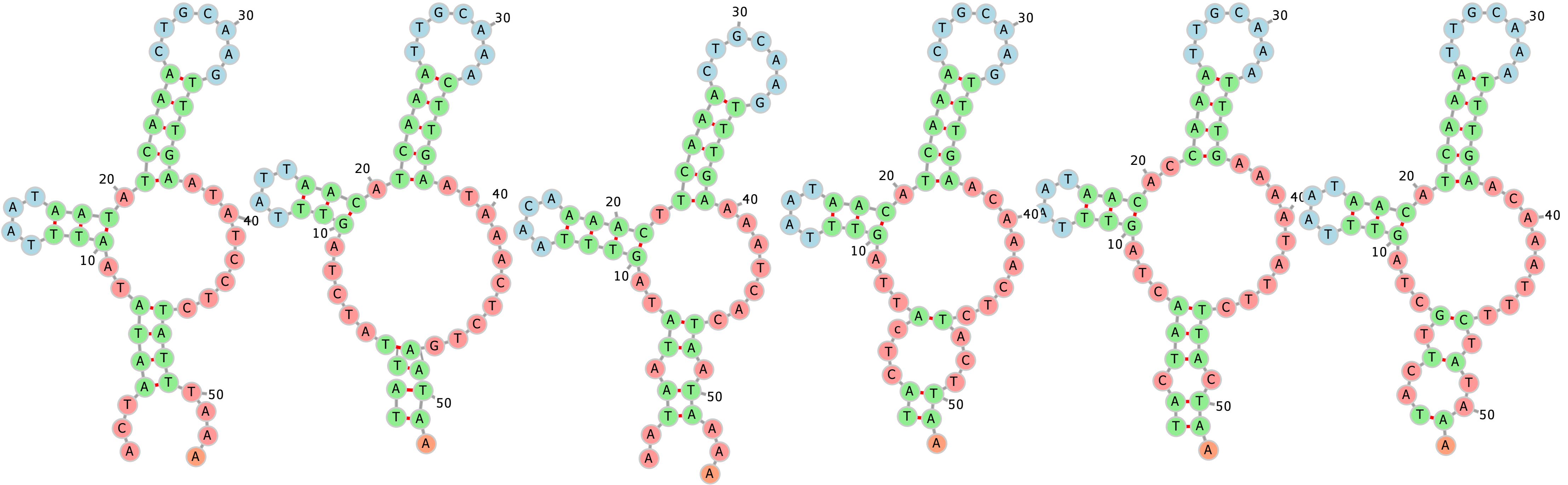

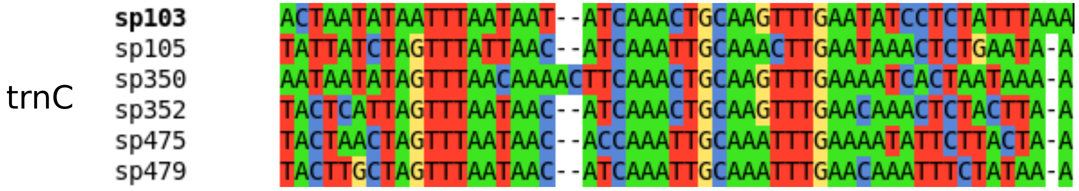
trnC
sp103 ACTAATATAATTTAATAAT--ATCAAACTGCAAGTTTGAATATCCTCTATTTAAA
sp105 TATTATCTAGTTTATTAAC--ATCAAATTGCAAACTTGAATAAACTCTGAATA-A
sp350 AATAATATAGTTTAACAAAACTTCAAACTGCAAGTTTGAAAATCACTAATAAA-A
sp352 TACTCATTAGTTTAATAAC--ATCAAACTGCAAGTTTGAACAAACTCTACTTA-A
sp475 TACTAACTAGTTTAATAAC--ACCAAATTGCAAATTTGAAAATATTCTTACTA-A
sp479 TACTTGCTAGTTTAATAAC--ATCAAATTGCAAATTTGAACAAATTTCTATAA-A

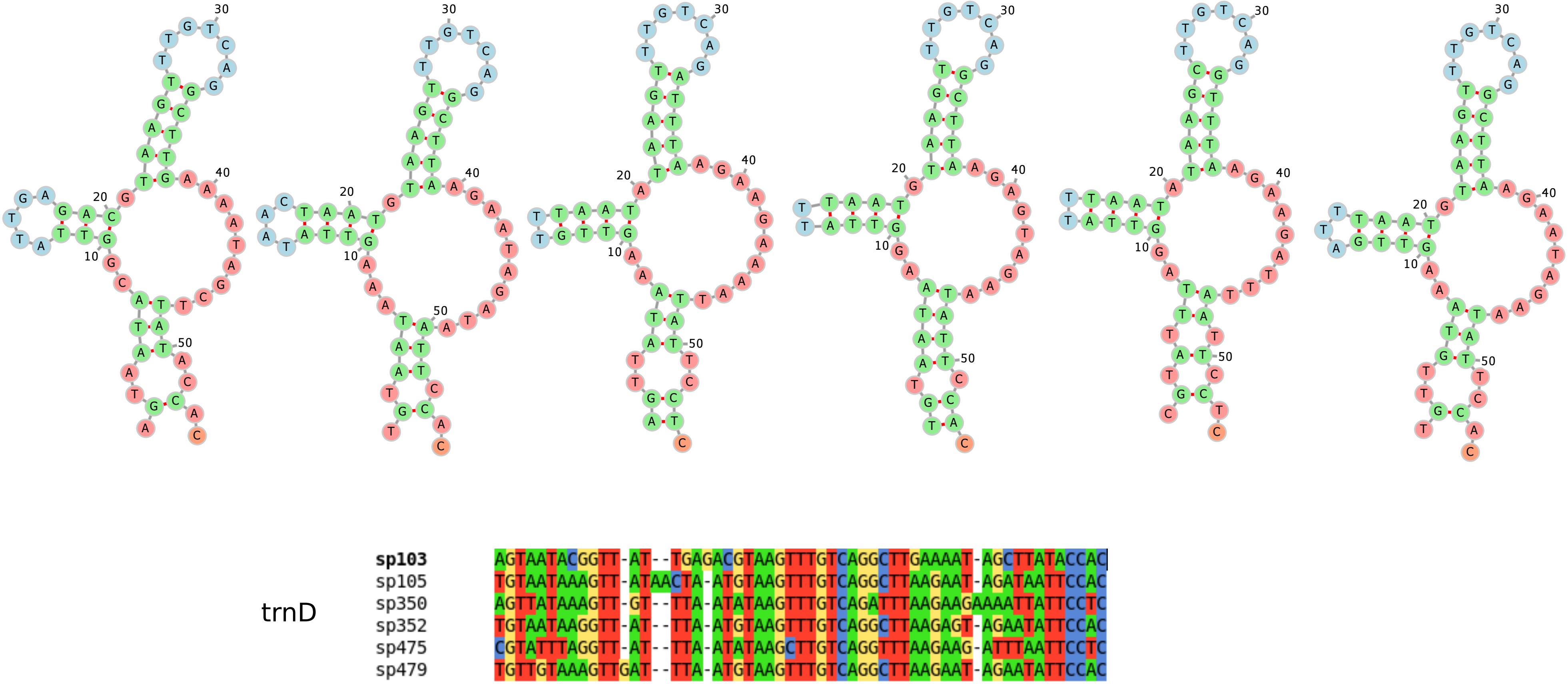
trnD
sp103 AGTAATACGGTT-AT--TGAGACGTAAGTTTGTCAGGCTTGAAAAT-AGCTTATACCAC
sp105 TGTAATAAAGTT-ATAACTA-ATGTAAGTTTGTCAGGCTTAAGAAT-AGATAATTCCAC
sp350 AGTTATAAAGTT-GT--TTA-ATATAAGTTTGTCAGATTTAAGAAGAAAATTATTCCTC
sp352 TGTAATAAGGTT-AT--TTA-ATGTAAGTTTGTCAGGCTTAAGAGT-AGAATATTCCAC
sp475 CGTATTTAGGTT-AT--TTA-ATATAAGCTTGTCAGGTTTAAGAAG-ATTTAATTCCTC
sp479 TGTTGTAAAGTTGAT--TTA-ATGTAAGTTTGTCAGGCTTAAGAAT-AGAATATTCCAC

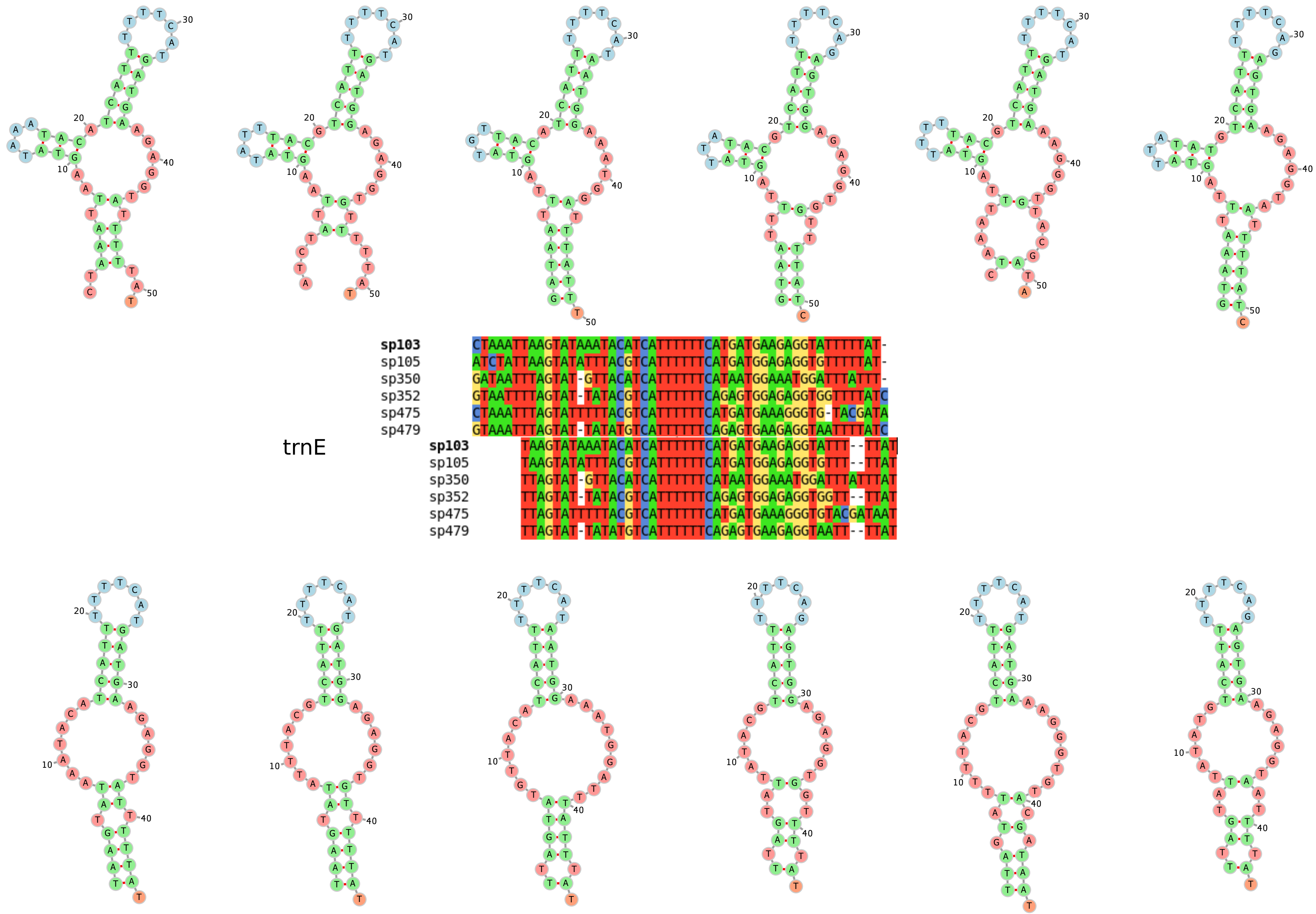
trnE
sp103 CTAAATTAAGTATAAATACATCATTTTTTCATGATGAAGAGGTATTTTTAT-
sp105 ATCTATTAAGTATATTTACGTCATTTTTTCATGATGGAGAGGTGTTTTTAT-
sp350 GATAATTTAGTAT-GTTACATCATTTTTTCATAATGGAAATGGATTTATTT-
sp352 GTAATTTTAGTAT-TATACGTCATTTTTTCAGAGTGGAGAGGTGGTTTTATC
sp475 CTAAATTTAGTATTTTTACGTCATTTTTTCATGATGAAAGGGTG-TACGATA
sp479 GTAAATTTAGTAT-TATATGTCATTTTTTCAGAGTGAAGAGGTAATTTTATC
sp103 TAAGTATAAATACATCATTTTTTCATGATGAAGAGGTATTT--TTAT
sp105 TAAGTATATTTACGTCATTTTTTCATGATGGAGAGGTGTTT--TTAT
sp350 TTAGTAT-GTTACATCATTTTTTCATAATGGAAATGGATTTATTTAT
sp352 TTAGTAT-TATACGTCATTTTTTCAGAGTGGAGAGGTGGTT--TTAT
sp475 TTAGTATTTTTACGTCATTTTTTCATGATGAAAGGGTGTACGATAAT
sp479 TTAGTAT-TATATGTCATTTTTTCAGAGTGAAGAGGTAATT--TTAT

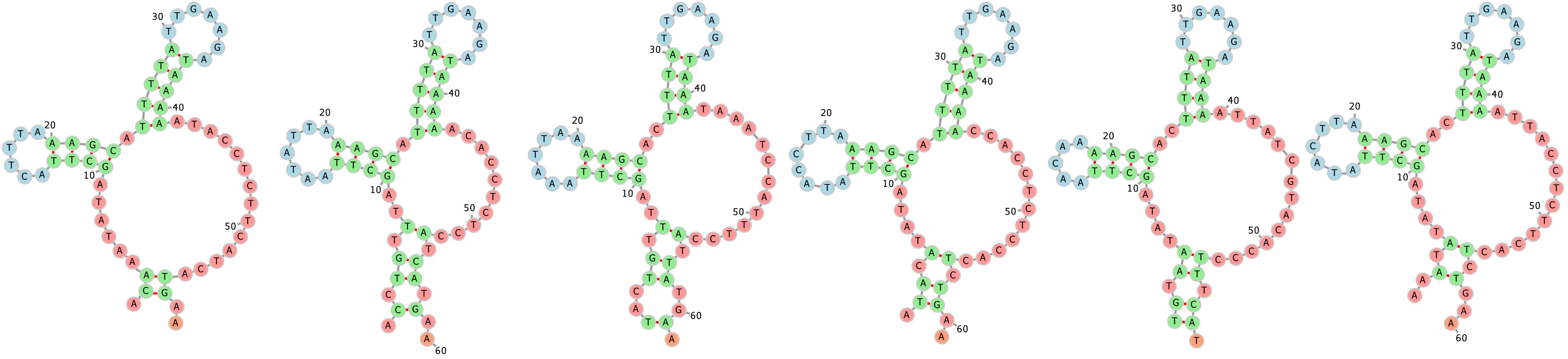

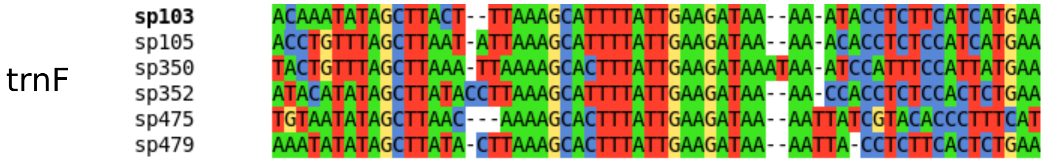
trnF
sp103 ACAAATATAGCTTACT--TTAAAGCATTTTATTGAAGATAA--AA-ATACCTCTTCATCATGAA
sp105 ACCTGTTTAGCTTAAT-ATTAAAGCATTTTATTGAAGATAA--AA-ACACCTCTCCATCATGAA
sp350 TACTGTTTAGCTTAAA-TTAAAAGCACTTTATTGAAGATAAATAA-ATCCATTTCCATTATGAA
sp352 ATACATATAGCTTATACCTTAAAGCATTTTATTGAAGATAA--AA-CCACCTCTCCACTCTGAA
sp475 TGTAATATAGCTTAAC---AAAAGCACTTTATTGAAGATAA--AATTATCGTACACCCTTTCAT
sp479 AAATATATAGCTTATA-CTTAAAGCACTTTATTGAAGATAA--AATTA-CCTCTTCACTCTGAA

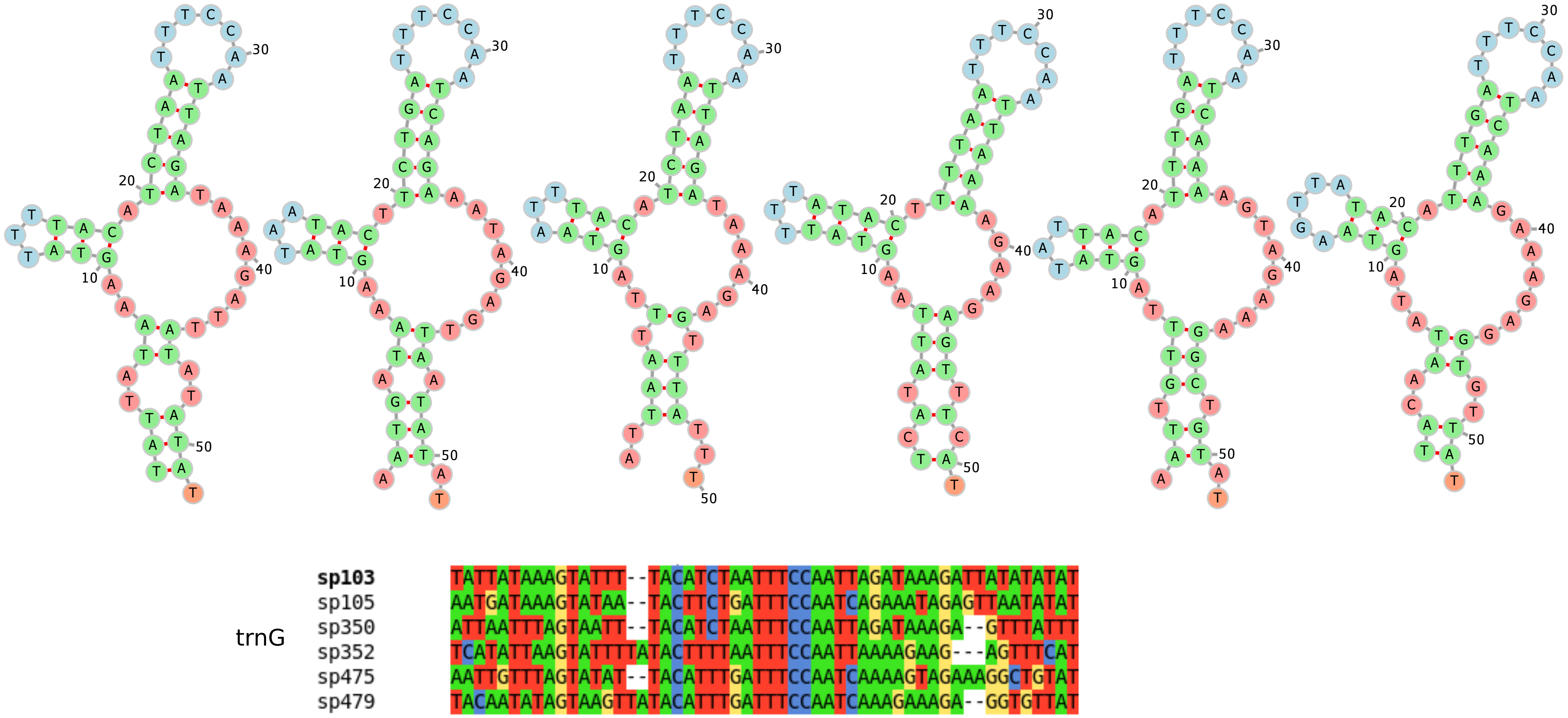
trnG
sp103 TATTATAAAGTATTT--TACATCTAATTTCCAATTAGATAAAGATTATATATAT
sp105 AATGATAAAGTATAA--TACTTCTGATTTCCAATCAGAAATAGAGTTAATATAT
sp350 ATTAATTTAGTAATT--TACATCTAATTTCCAATTAGATAAAGA--GTTTATTT
sp352 TCATATTAAGTATTTTATACTTTTAATTTCCAATTAAAAGAAG---AGTTTCAT
sp475 AATTGTTTAGTATAT--TACATTTGATTTCCAATCAAAAGTAGAAAGGCTGTAT
sp479 TACAATATAGTAAGTTATACATTTGATTTCCAATCAAAGAAAGA--GGTGTTAT

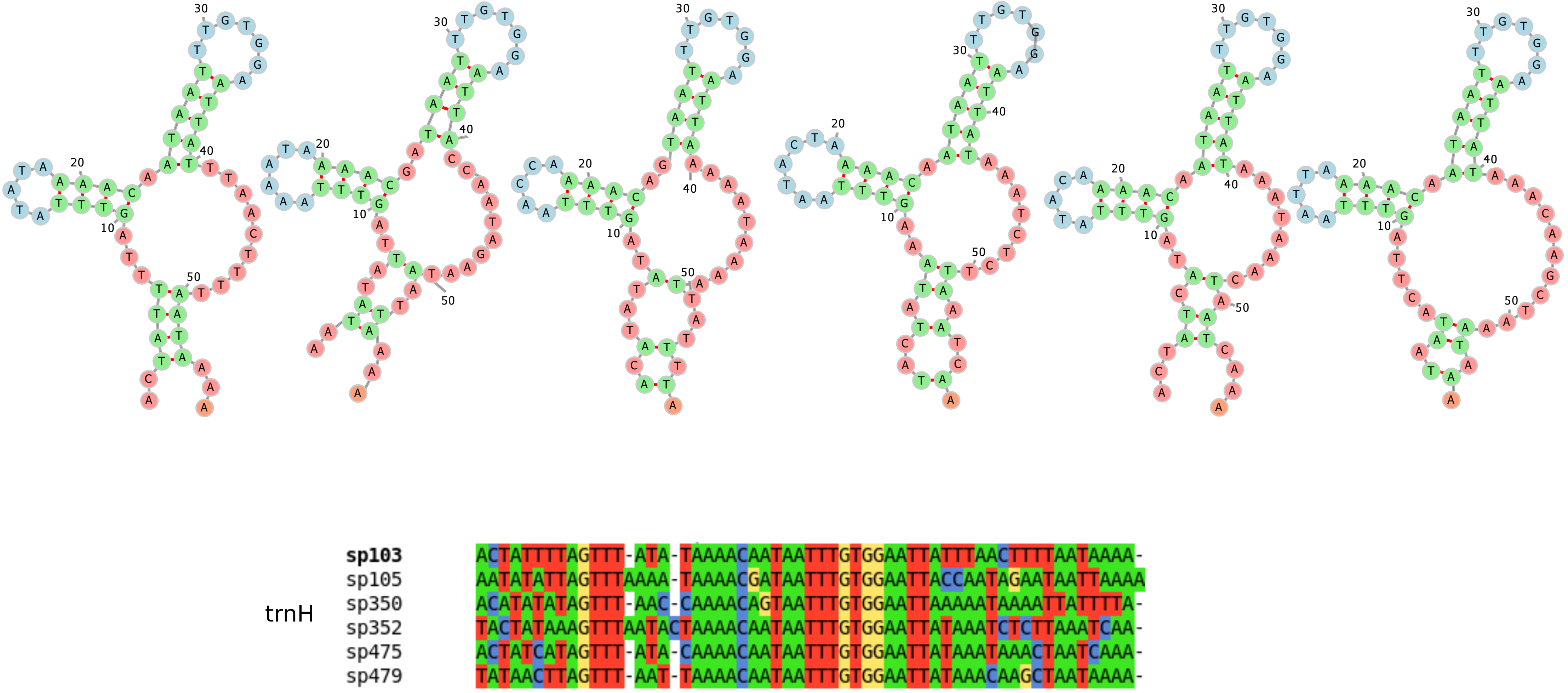
trnH
sp103 ACTATTTTAGTTT-ATA-TAAAACAATAATTTGTGGAATTATTTAACTTTTAATAAAA-
sp105 AATATATTAGTTTAAAA-TAAAACGATAATTTGTGGAATTACCAATAGAATAATTAAAA
sp350 ACATATATAGTTT-AAC-CAAAACAGTAATTTGTGGAATTAAAAATAAAATTATTTTA-
sp352 TACTATAAAGTTTAATACTAAAACAATAATTTGTGGAATTATAAATCTCTTAAATCAA-
sp475 ACTATCATAGTTT-ATA-CAAAACAATAATTTGTGGAATTATAAATAAACTAATCAAA-
sp479 TATAACTTAGTTT-AAT-TAAAACAATAATTTGTGGAATTATAAACAAGCTAATAAAA-

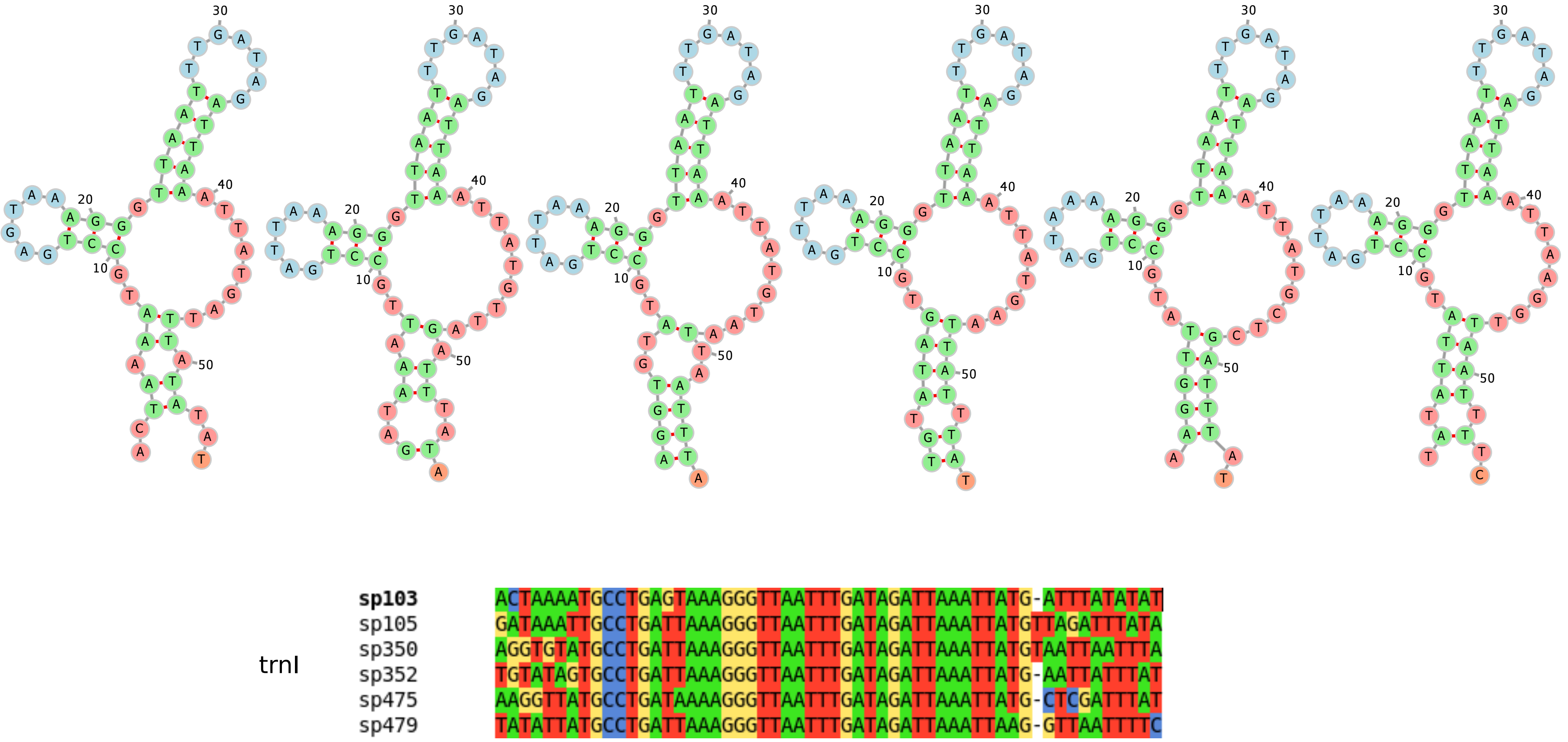
trnI
sp103 ACTAAAATGCCTGAGTAAAGGGTTAATTTGATAGATTAAATTATG-ATTTATATAT
sp105 GATAAATTGCCTGATTAAAGGGTTAATTTGATAGATTAAATTATGTTAGATTTATA
sp350 AGGTGTATGCCTGATTAAAGGGTTAATTTGATAGATTAAATTATGTAATTAATTTA
sp352 TGTATAGTGCCTGATTAAAGGGTTAATTTGATAGATTAAATTATG-AATTATTTAT
sp475 AAGGTTATGCCTGATAAAAGGGTTAATTTGATAGATTAAATTATG-CTCGATTTAT
sp479 TATATTATGCCTGATTAAAGGGTTAATTTGATAGATTAAATTAAG-GTTAATTTTC

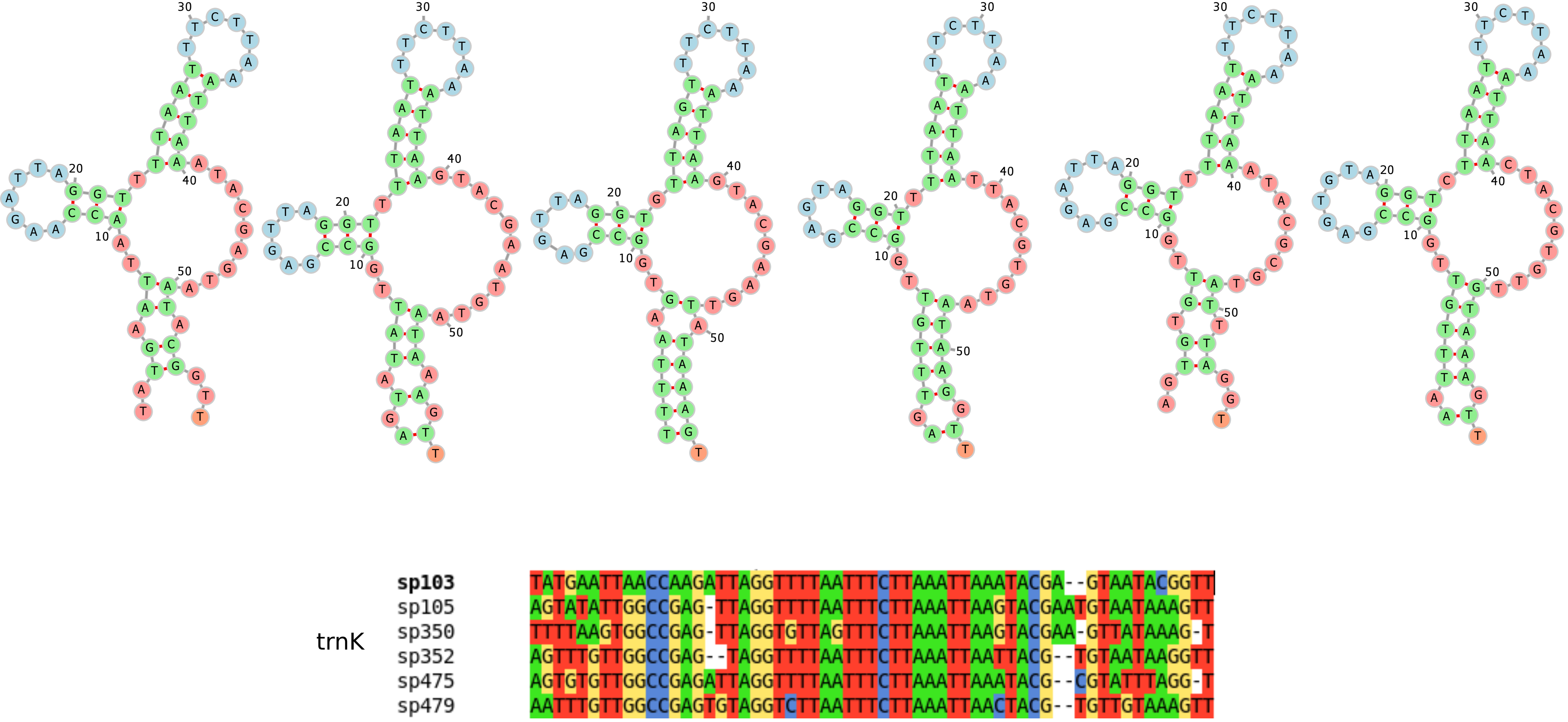
trnK
sp103 TATGAATTAACCAAGATTAGGTTTTAATTTCTTAAATTAAATACGA--GTAATACGGTT
sp105 AGTATATTGGCCGAG-TTAGGTTTTAATTTCTTAAATTAAGTACGAATGTAATAAAGTT
sp350 TTTTAAGTGGCCGAG-TTAGGTGTTAGTTTCTTAAATTAAGTACGAA-GTTATAAAG-T
sp352 AGTTTGTTGGCCGAG--TAGGTTTTAATTTCTTAAATTAATTACG--TGTAATAAGGTT
sp475 AGTGTGTTGGCCGAGATTAGGTTTTAATTTCTTAAATTAAATACG--CGTATTTAGG-T
sp479 AATTTGTTGGCCGAGTGTAGGTCTTAATTTCTTAAATTAACTACG--TGTTGTAAAGTT

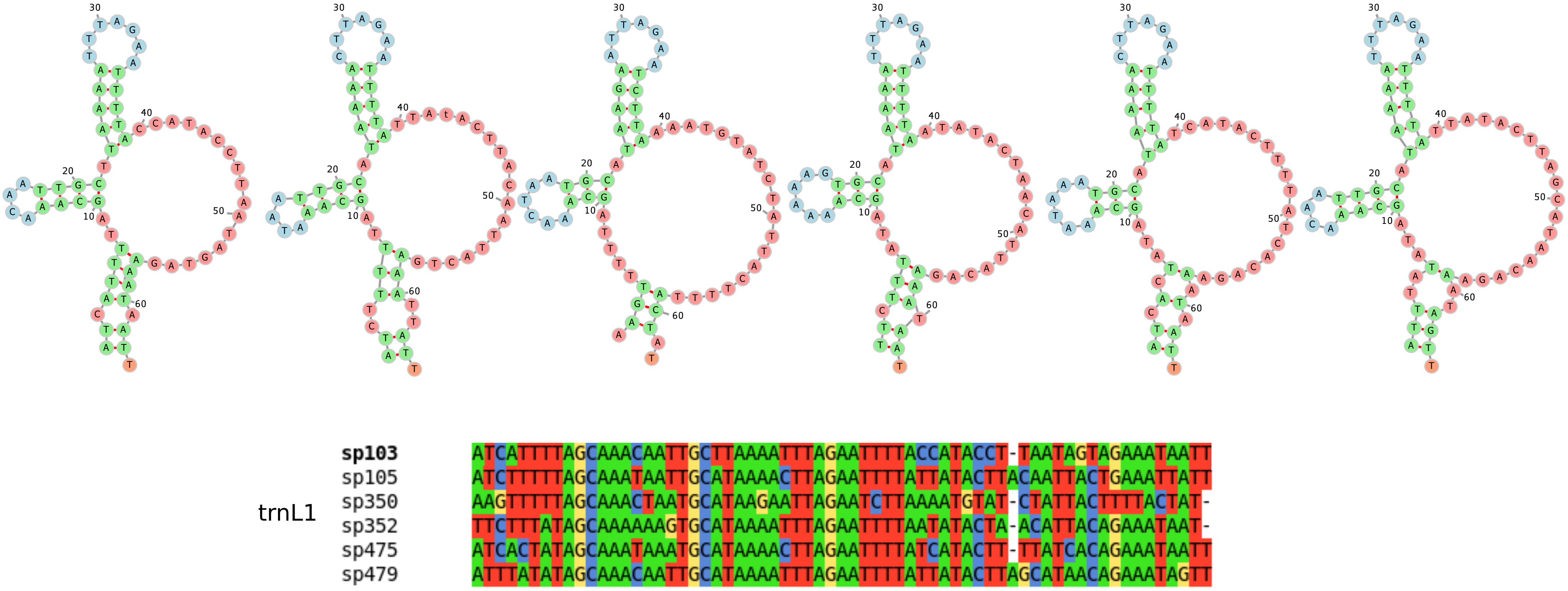
trnL1
sp103 ATCATTTTAGCAAACAATTGCTTAAAATTTAGAATTTTACCATACCT-TAATAGTAGAAATAATT
sp105 ATCTTTTTAGCAAATAATTGCATAAAACTTAGAATTTTATTATACTTACAATTACTGAAATTATT
sp350 AAGTTTTTAGCAAACTAATGCATAAGAATTAGAATCTTAAAATGTAT-CTATTACTTTTACTAT-
sp352 TTCTTTATAGCAAAAAAGTGCATAAAATTTAGAATTTTAATATACTA-ACATTACAGAAATAAT-
sp475 ATCACTATAGCAAATAAATGCATAAAACTTAGAATTTTATCATACTT-TTATCACAGAAATAATT
sp479 ATTTATATAGCAAACAATTGCATAAAATTTAGAATTTTATTATACTTAGCATAACAGAAATAGTT

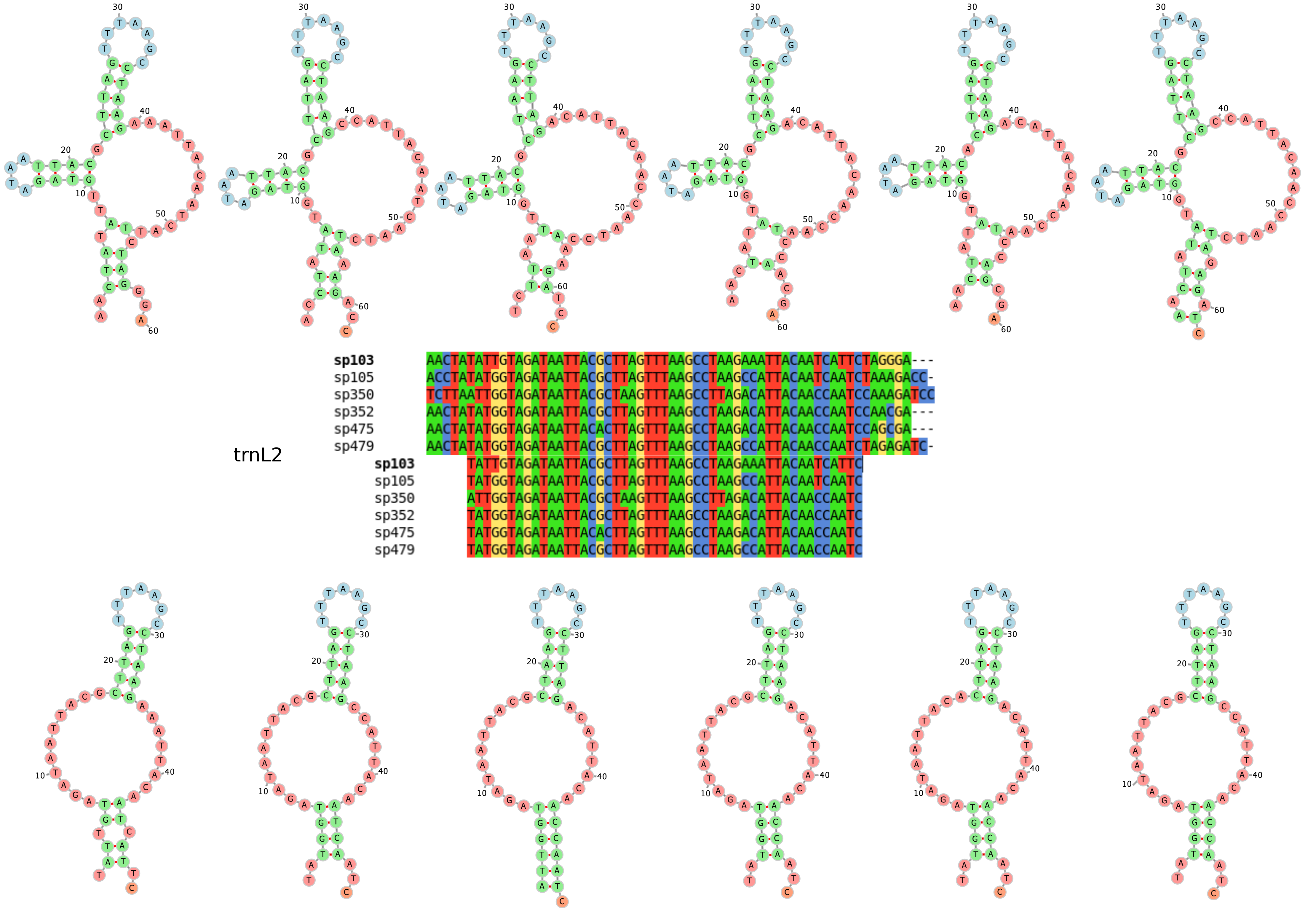

trnL2
sp103 AACTATATTGTAGATAATTACGCTTAGTTTAAGCCTAAGAAATTACAATCATTCTAGGGA---
sp105 ACCTATATGGTAGATAATTACGCTTAGTTTAAGCCTAAGCCATTACAATCAATCTAAAGACC-
sp350 TCTTAATTGGTAGATAATTACGCTAAGTTTAAGCCTTAGACATTACAACCAATCCAAAGATCC
sp352 AACTATATGGTAGATAATTACGCTTAGTTTAAGCCTAAGACATTACAACCAATCCAACGA---
sp475 AACTATATGGTAGATAATTACACTTAGTTTAAGCCTAAGACATTACAACCAATCCAGCGA---
sp479 AACTATATGGTAGATAATTACGCTTAGTTTAAGCCTAAGCCATTACAACCAATCTAGAGATC-
sp103 TATTGTAGATAATTACGCTTAGTTTAAGCCTAAGAAATTACAATCATTC
sp105 TATGGTAGATAATTACGCTTAGTTTAAGCCTAAGCCATTACAATCAATC
sp350 ATTGGTAGATAATTACGCTAAGTTTAAGCCTTAGACATTACAACCAATC
sp352 TATGGTAGATAATTACGCTTAGTTTAAGCCTAAGACATTACAACCAATC
sp475 TATGGTAGATAATTACACTTAGTTTAAGCCTAAGACATTACAACCAATC
sp479 TATGGTAGATAATTACGCTTAGTTTAAGCCTAAGCCATTACAACCAATC

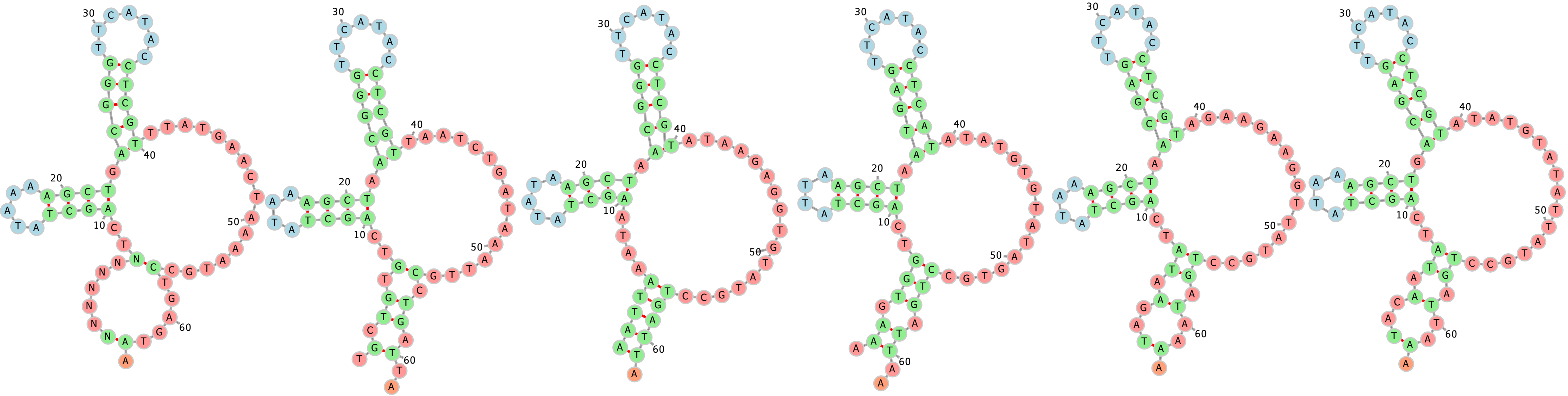

trnM

```
sp103  NNNNNNNTCAGCTATAAAAGCTGACGGGTTCATACCTCGTTTATGAACTAAAATGCCTGAGTAA-
sp105  TGCTGTGTCAGCTATA-AAGCTAACGGGTTCATACCTCGTTAATCTGATAAATTGCCTGATTA--
sp350  AATTAAATAAGCTATATAAGCTAACGGGTTCATACCTCGTATAAGAGGTGTA-TGCCTGATTA--
sp352  AAAGTGGTCAGCTATT-AAGCTAATGAGTTCATACCTCATATATGTGTATAG-TGCCTGATTAA-
sp475  TAGAATATCAGCTATA-AAGCTAACGAGTTCATACCTCGTAGAAGAAGGTTA-TGCCTGATAAAA
sp479  TACAATATCAGCTATA-AAGCTGACGAGTTCATACCTCGTATATGTATATTA-TGCCTGATTAAA
```

```
sp103  TCAGCTATAAAAGCTGACGGGTTCATACCTCGTTTATGAACTAAAATGCCTGAG
sp105  TCAGCTATA-AAGCTAACGGGTTCATACCTCGTTAATCTGATAAATTGCCTGAT
sp350  TAAGCTATATAAGCTAACGGGTTCATACCTCGTATAAGAG-GTGTATGCCTGAT
sp352  TCAGCTATT-AAGCTAATGAGTTCATACCTCATATATGTG-TATAGTGCCTGAT
sp475  TCAGCTATA-AAGCTAACGAGTTCATACCTCGTAGAAGAA-GGTTATGCCTGAT
sp479  TCAGCTATA-AAGCTGACGAGTTCATACCTCGTATATGTA-TATTATGCCTGAT
```

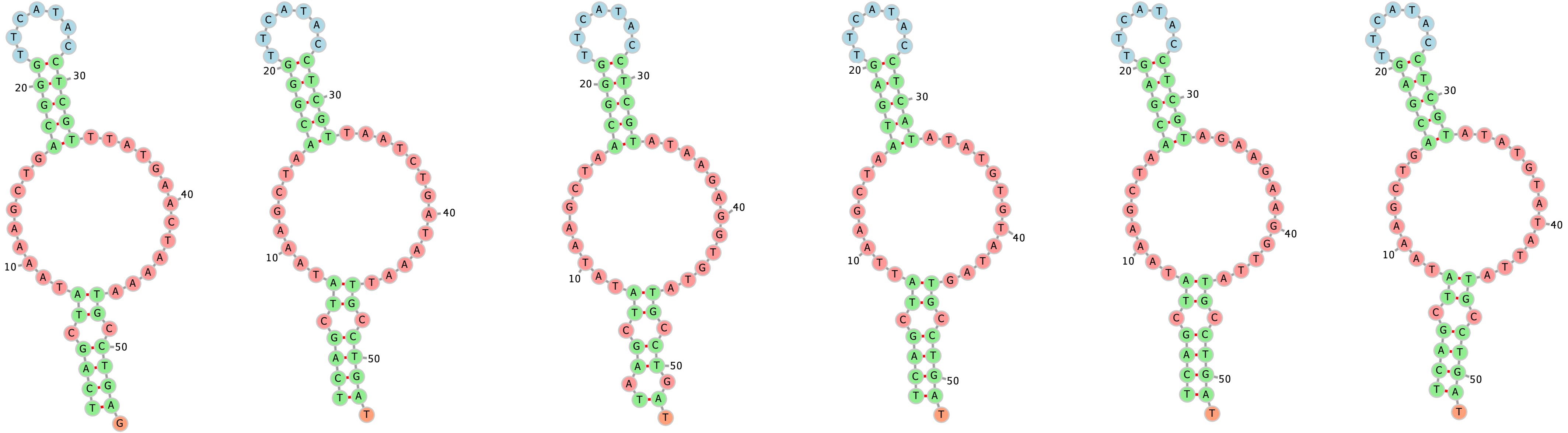

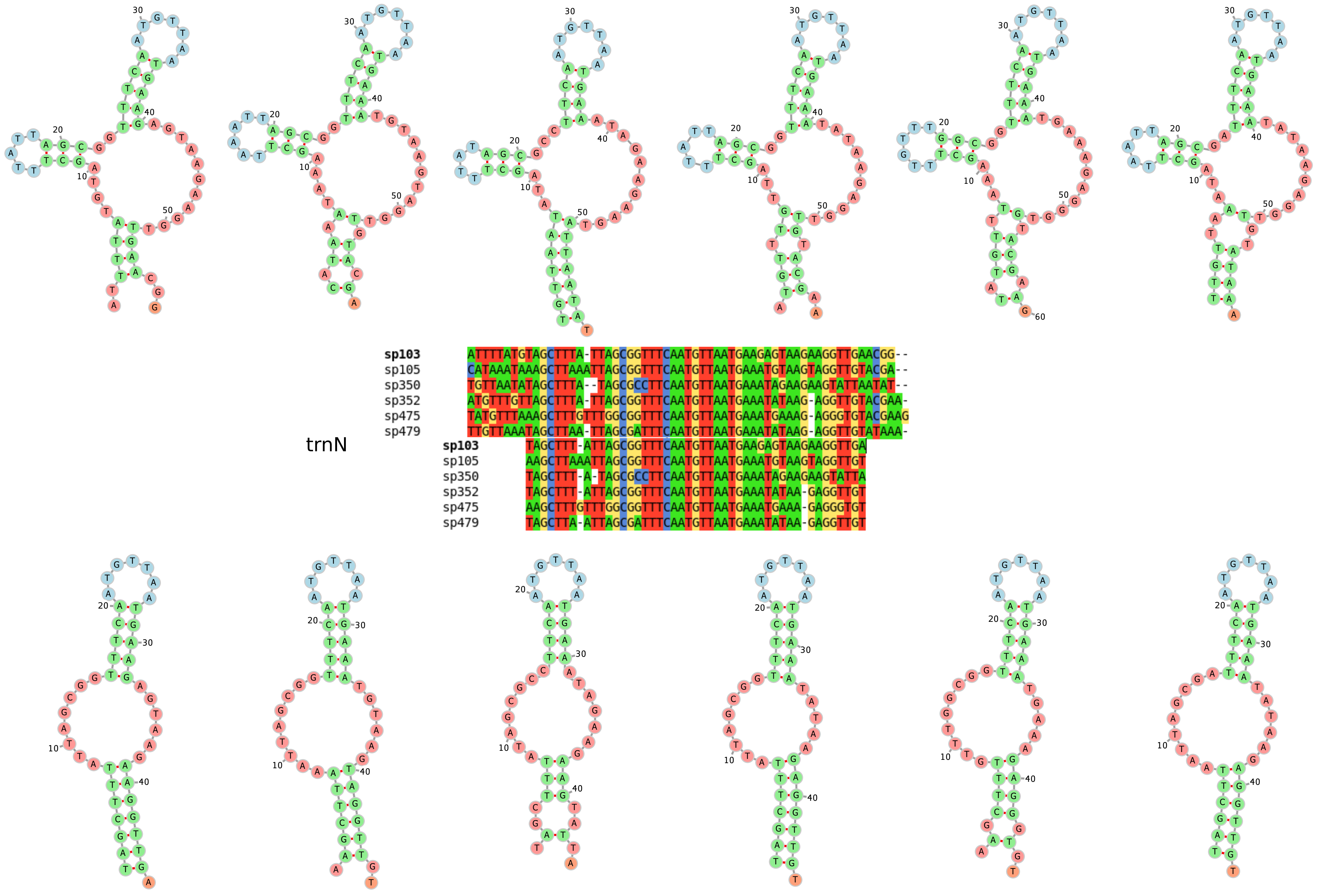
trnN
sp103 ATTTTATGTAGCTTTA-TTAGCGGTTTCAATGTTAATGAAGAGTAAGAAGGTTGAACGG--
sp105 CATAAATAAAGCTTAAATTAGCGGTTTCAATGTTAATGAAATGTAAGTAGGTTGTACGA--
sp350 TGTTAATATAGCTTTA--TAGCGCCTTCAATGTTAATGAAATAGAAGAAGTATTAATAT--
sp352 ATGTTTGTTAGCTTTA-TTAGCGGTTTCAATGTTAATGAAATATAAG-AGGTTGTACGAA-
sp475 TATGTTTAAAGCTTTGTTTGGCGGTTTCAATGTTAATGAAATGAAAG-AGGGTGTACGAAG
sp479 TTGTTAAATAGCTTAA-TTAGCGATTTCAATGTTAATGAAATATAAG-AGGTTGTATAAA-
sp103 TAGCTTT-ATTAGCGGTTTCAATGTTAATGAAGAGTAAGAAGGTTGA
sp105 AAGCTTAAATTAGCGGTTTCAATGTTAATGAAATGTAAGTAGGTTGT
sp350 TAGCTTT-A-TAGCGCCTTCAATGTTAATGAAATAGAAGAAGTATTA
sp352 TAGCTTT-ATTAGCGGTTTCAATGTTAATGAAATATAA-GAGGTTGT
sp475 AAGCTTTGTTTGGCGGTTTCAATGTTAATGAAATGAAA-GAGGGTGT
sp479 TAGCTTA-ATTAGCGATTTCAATGTTAATGAAATATAA-GAGGTTGT

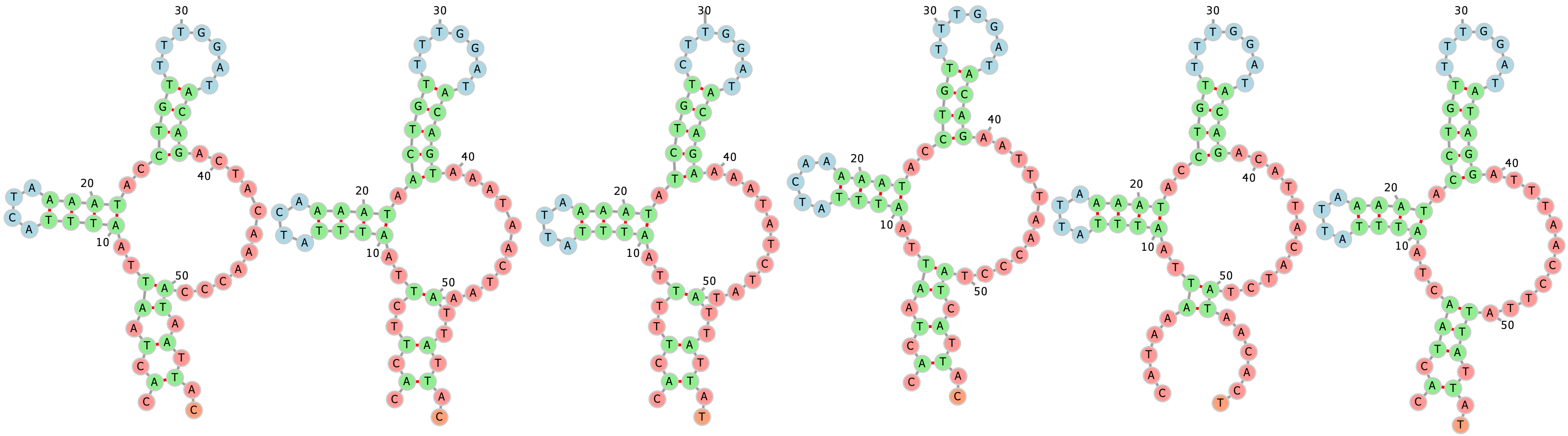

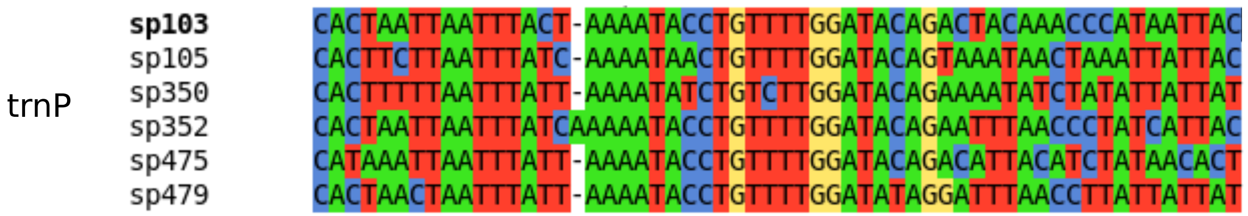

trnP
sp103 CACTAATTAATTTACT-AAAATACCTGTTTTGGATACAGACTACAAACCCATAATTAC
sp105 CACTTCTTAATTTATC-AAAATAACTGTTTTGGATACAGTAAATAACTAAATTATTAC
sp350 CACTTTTTAATTTATT-AAAATATCTGTCTTGGATACAGAAAATATCTATATTATTAT
sp352 CACTAATTAATTTATCAAAAATACCTGTTTTGGATACAGAATTTAACCCTATCATTAC
sp475 CATAAATTAATTTATT-AAAATACCTGTTTTGGATACAGACATTACATCTATAACACT
sp479 CACTAACTAATTTATT-AAAATACCTGTTTTGGATATAGGATTTAACCTTATTATTAT

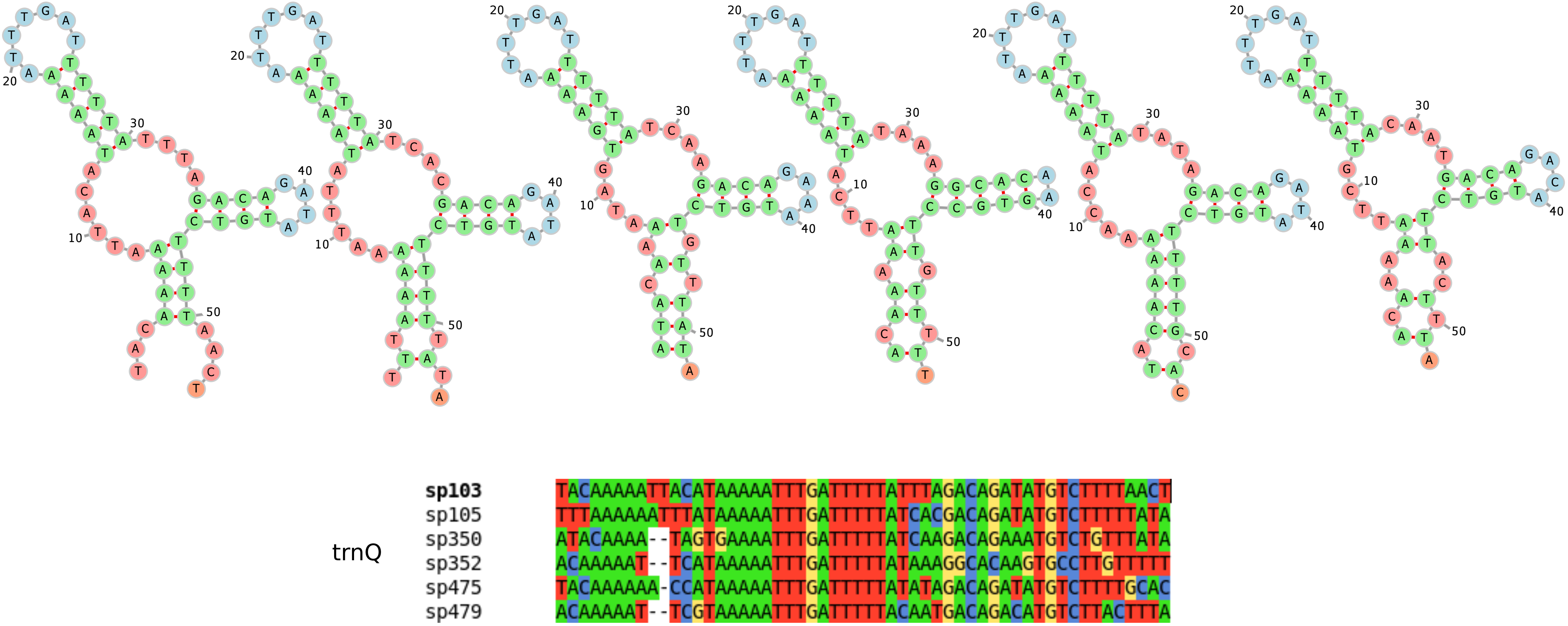
trnQ
sp103 TACAAAAATTACATAAAAATTTGATTTTTATTTAGACAGATATGTCTTTTAACT
sp105 TTTAAAAAATTTATAAAAATTTGATTTTTATCACGACAGATATGTCTTTTTATA
sp350 ATACAAAA--TAGTGAAAATTTGATTTTTATCAAGACAGAAATGTCTGTTTATA
sp352 ACAAAAAT--TCATAAAAATTTGATTTTTATAAAGGCACAAGTGCCTTGTTTTT
sp475 TACAAAAAA-CCATAAAAATTTGATTTTTATATAGACAGATATGTCTTTTGCAC
sp479 ACAAAAAT--TCGTAAAAATTTGATTTTTACAATGACAGACATGTCTTACTTTA

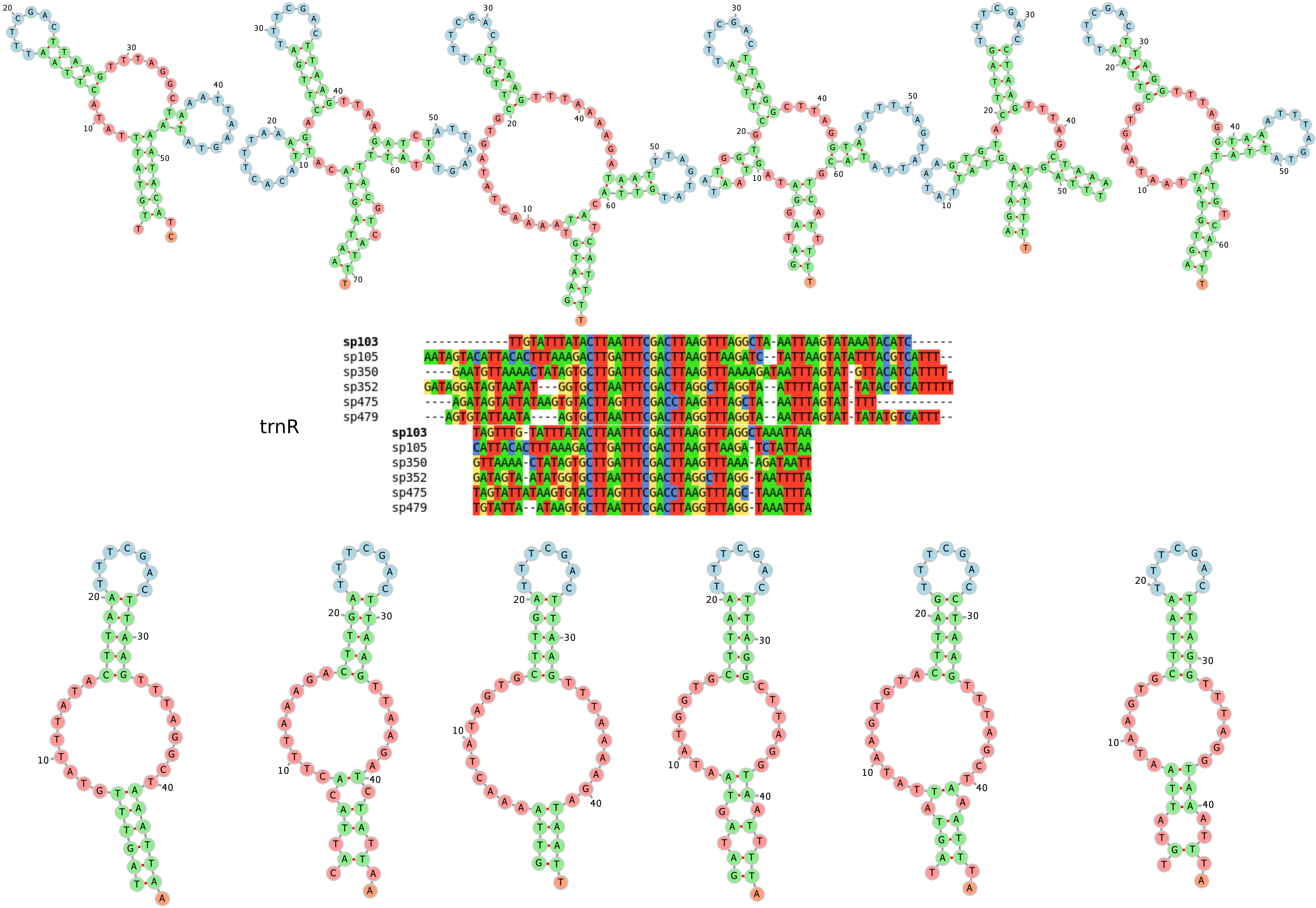
trnR
sp103 ------------TTGTATTTATACTTAATTTCGACTTAAGTTTAGGCTA-AATTAAGTATAAATACATC------
sp105 AATAGTACATTACACTTTAAAGACTTGATTTCGACTTAAGTTAAGATC--TATTAAGTATATTTACGTCATTT--
sp350 ----GAATGTTAAAACTATAGTGCTTGATTTCGACTTAAGTTTAAAAGATAATTTAGTAT-GTTACATCATTTT-
sp352 GATAGGATAGTAATAT---GGTGCTTAATTTCGACTTAGGCTTAGGTA--ATTTTAGTAT-TATACGTCATTTTT
sp475 ----AGATAGTATTATAAGTGTACTTAGTTTCGACCTAAGTTTAGCTA--AATTTAGTAT-TTT-----------
sp479 ---AGTGTATTAATA----AGTGCTTAATTTCGACTTAGGTTTAGGTA--AATTTAGTAT-TATATGTCATTT--
sp103 TAGTTTG-TATTTATACTTAATTTCGACTTAAGTTTAGGCTAAATTAA
sp105 CATTACACTTTAAAGACTTGATTTCGACTTAAGTTAAGA-TCTATTAA
sp350 GTTAAAA-CTATAGTGCTTGATTTCGACTTAAGTTTAAA-AGATAATT
sp352 GATAGTA-ATATGGTGCTTAATTTCGACTTAGGCTTAGG-TAATTTTA
sp475 TAGTATTATAAGTGTACTTAGTTTCGACCTAAGTTTAGC-TAAATTTA
sp479 TGTATTA--ATAAGTGCTTAATTTCGACTTAGGTTTAGG-TAAATTTA

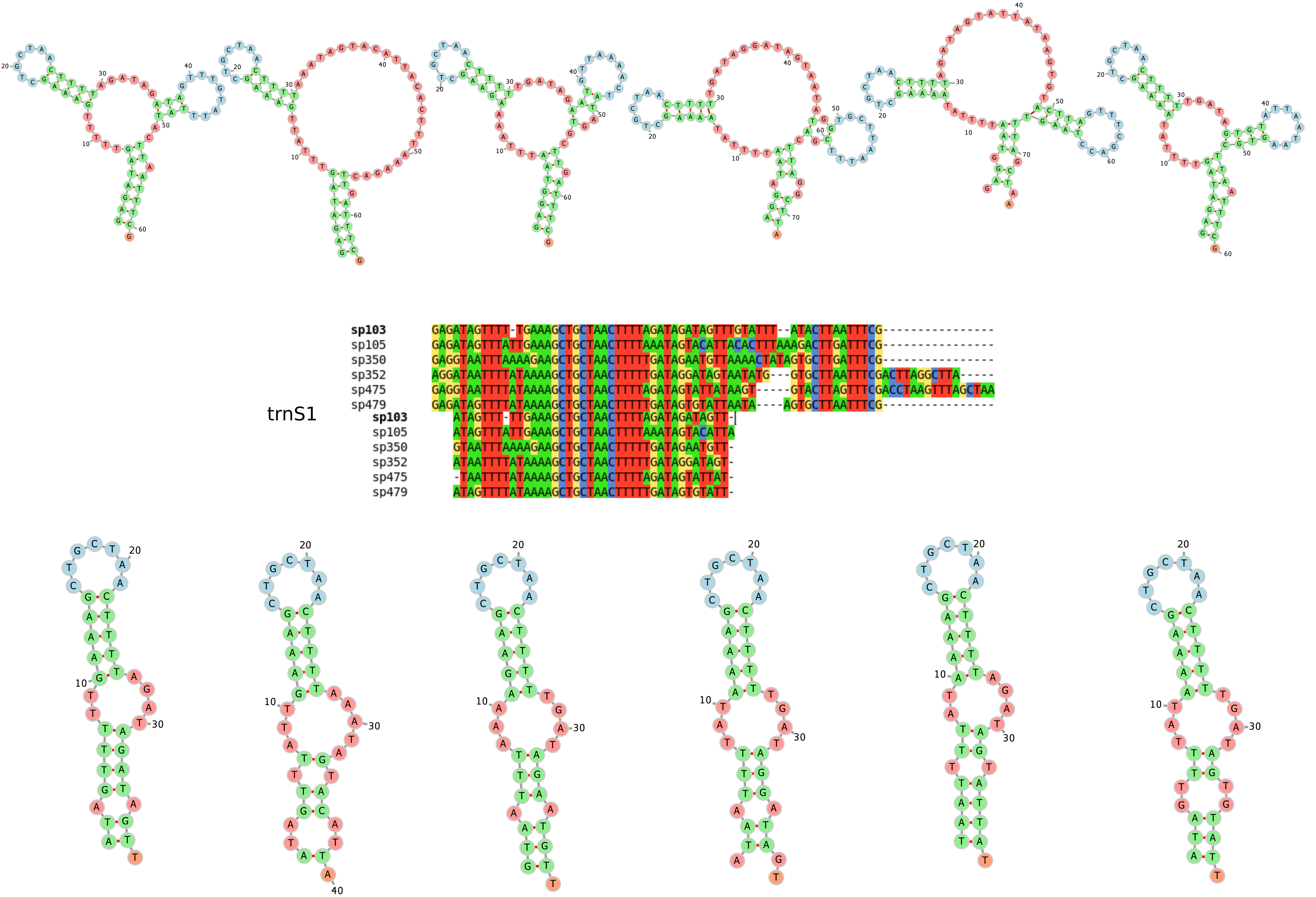
trnS1
sp103 GAGATAGTTTT-TGAAAGCTGCTAACTTTTAGATAGATAGTTTGTATTT--ATACTTAATTTCG---------------
sp105 GAGATAGTTTATTGAAAGCTGCTAACTTTTAAATAGTACATTACACTTTAAAGACTTGATTTCG---------------
sp350 GAGGTAATTTAAAAGAAGCTGCTAACTTTTTGATAGAATGTTAAAACTATAGTGCTTGATTTCG---------------
sp352 AGGATAATTTTATAAAAGCTGCTAACTTTTTGATAGGATAGTAATATG---GTGCTTAATTTCGACTTAGGCTTA-----
sp475 GAGGTAATTTTATAAAAGCTGCTAACTTTTAGATAGTATTATAAGT-----GTACTTAGTTTCGACCTAAGTTTAGCTAA
sp479 GAGATAGTTTTATAAAAGCTGCTAACTTTTTGATAGTGTATTAATA----AGTGCTTAATTTCG---------------
sp103 ATAGTTT-TTGAAAGCTGCTAACTTTTAGATAGATAGTT-[
sp105 ATAGTTTATTGAAAGCTGCTAACTTTTAAATAGTACATTA
sp350 GTAATTTAAAAGAAGCTGCTAACTTTTTGATAGAATGTT-
sp352 ATAATTTTATAAAAGCTGCTAACTTTTTGATAGGATAGT-
sp475 -TAATTTTATAAAAGCTGCTAACTTTTAGATAGTATTAT-
sp479 ATAGTTTTATAAAAGCTGCTAACTTTTTGATAGTGTATT-

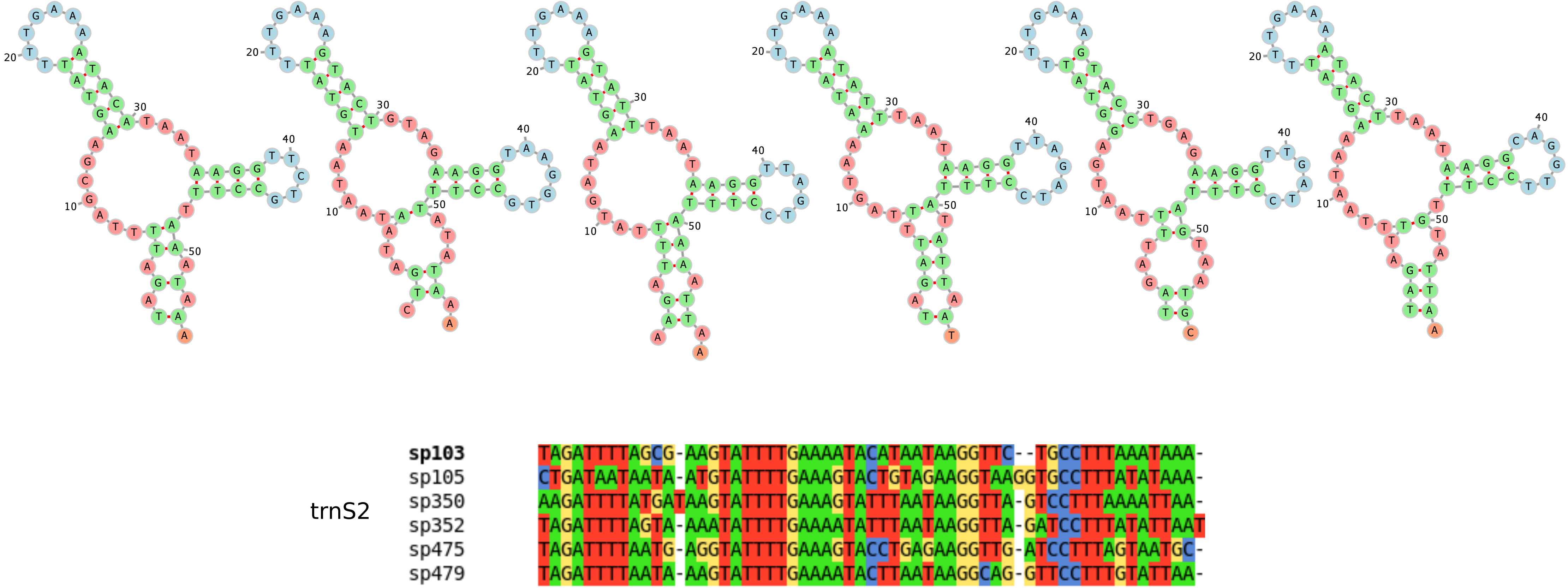
trnS2
sp103 TAGATTTTAGCG-AAGTATTTTGAAAATACATAATAAGGTTC--TGCCTTTAAATAAA-
sp105 CTGATAATAATA-ATGTATTTTGAAAGTACTGTAGAAGGTAAGGTGCCTTTATATAAA-
sp350 AAGATTTTATGATAAGTATTTTGAAAGTATTTAATAAGGTTA-GTCCTTTAAAATTAA-
sp352 TAGATTTTAGTA-AAATATTTTGAAAATATTTAATAAGGTTA-GATCCTTTATATTAAT
sp475 TAGATTTTAATG-AGGTATTTTGAAAGTACCTGAGAAGGTTG-ATCCTTTAGTAATGC-
sp479 TAGATTTTAATA-AAGTATTTTGAAAATACTTAATAAGGCAG-GTTCCTTTGTATTAA-

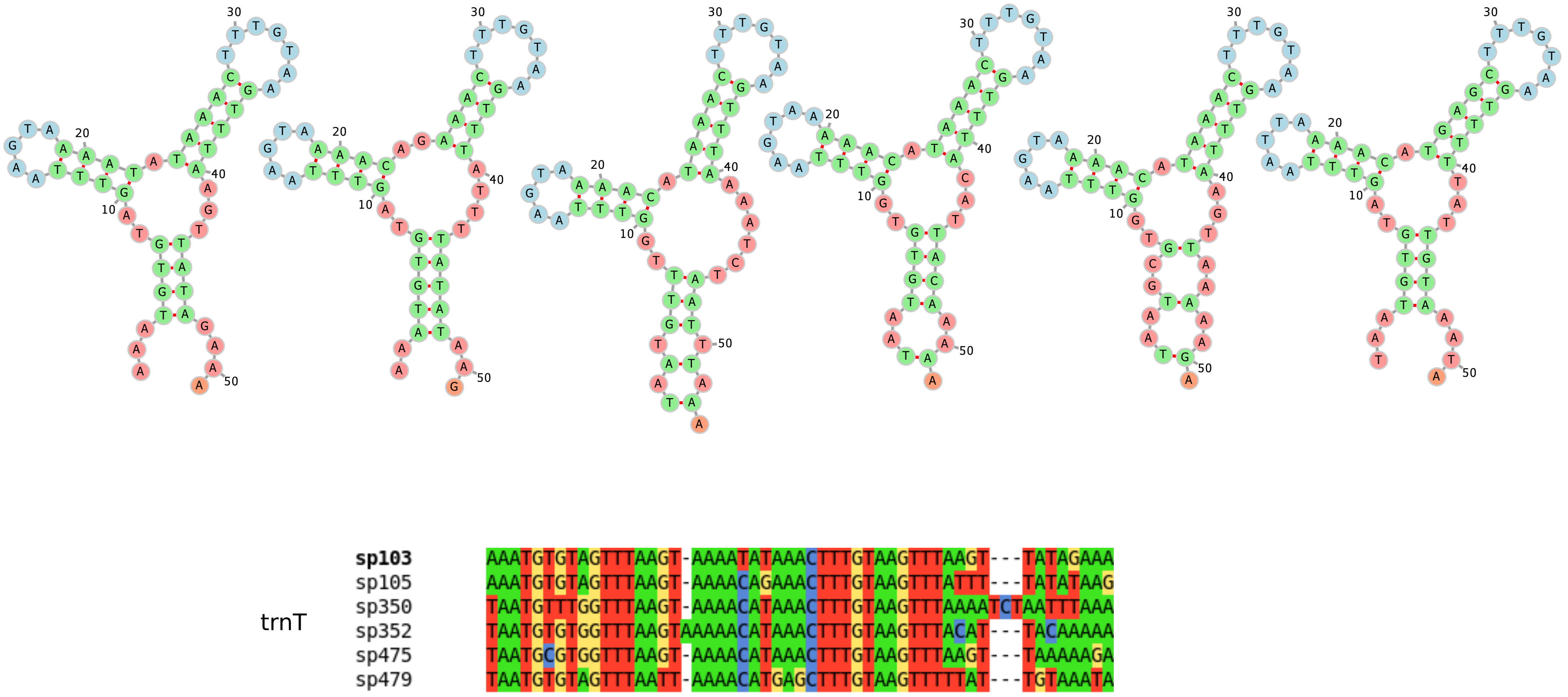
trnT
sp103 AAATGTGTAGTTTAAGT-AAAATATAAACTTTGTAAGTTTAAGT---TATAGAAA
sp105 AAATGTGTAGTTTAAGT-AAAACAGAAACTTTGTAAGTTTATTT---TATATAAG
sp350 TAATGTTTGGTTTAAGT-AAAACATAAACTTTGTAAGTTTAAAATCTAATTTAAA
sp352 TAATGTGTGGTTTAAGTAAAAACATAAACTTTGTAAGTTTACAT---TACAAAAA
sp475 TAATGCGTGGTTTAAGT-AAAACATAAACTTTGTAAGTTTAAGT---TAAAAAGA
sp479 TAATGTGTAGTTTAATT-AAAACATGAGCTTTGTAAGTTTTTAT---TGTAAATA

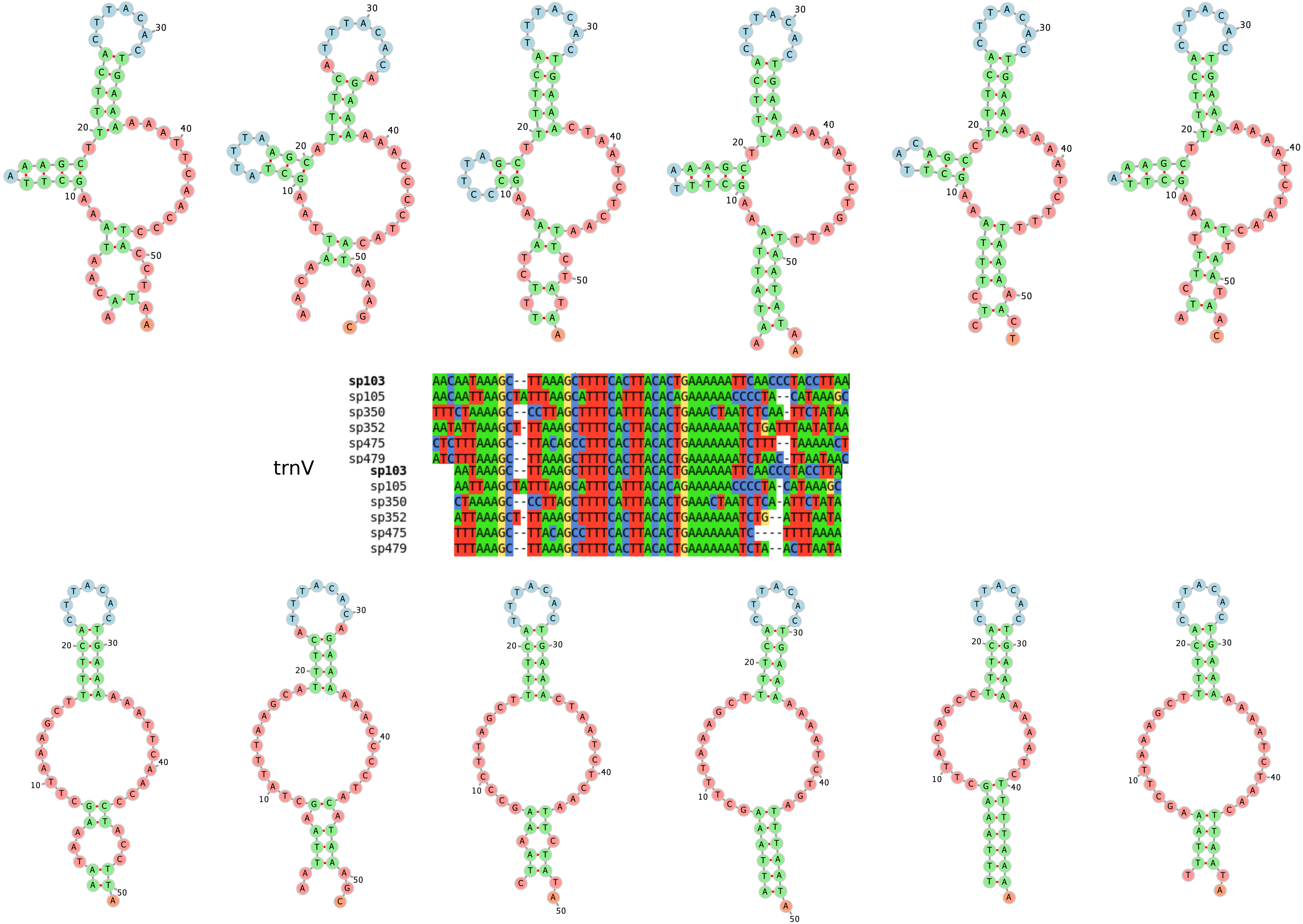
trnV
sp103 AACAATAAAGC--TTAAAGCTTTTCACTTACACTGAAAAAATTCAACCCTACCTTAA
sp105 AACAATTAAGCTATTTAAGCATTTCATTTACACAGAAAAAACCCCTA--CATAAAGC
sp350 TTTCTAAAAGC--CCTTAGCTTTTCATTTACACTGAAACTAATCTCAA-TTCTATAA
sp352 AATATTAAAGCT-TTAAAGCTTTTCACTTACACTGAAAAAAATCTGATTTAATATAA
sp475 CTCTTTAAAGC--TTACAGCCTTTCACTTACACTGAAAAAAATCTTT--TAAAAACT
sp479 ATCTTTAAAGC--TTAAAGCTTTTCACTTACACTGAAAAAAATCTAAC-TTAATAAC
sp103 AATAAAGC--TTAAAGCTTTTCACTTACACTGAAAAAATTCAACCCTACCTTA
sp105 AATTAAGCTATTTAAGCATTTCATTTACACAGAAAAAACCCCTA-CATAAAGC
sp350 CTAAAAGC--CCTTAGCTTTTCATTTACACTGAAACTAATCTCA-ATTCTATA
sp352 ATTAAAGCT-TTAAAGCTTTTCACTTACACTGAAAAAAATCTG--ATTTAATA
sp475 TTTAAAGC--TTACAGCCTTTCACTTACACTGAAAAAAATC----TTTTAAAA
sp479 TTTAAAGC--TTAAAGCTTTTCACTTACACTGAAAAAAATCTA--ACTTAATA

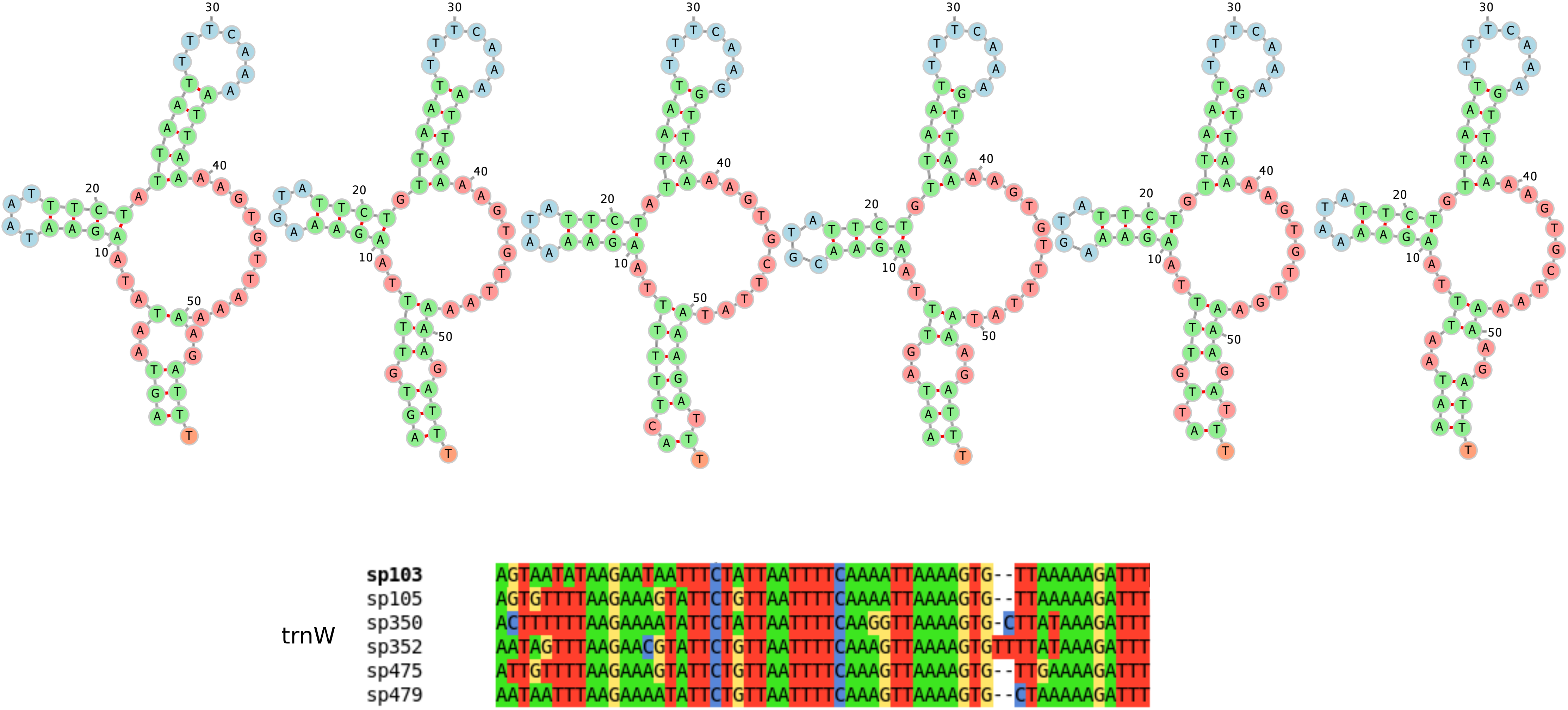
trnW
sp103 AGTAATATAAGAATAATTTCTATTAATTTTCAAAATTAAAAGTG--TTAAAAAGATTT
sp105 AGTGTTTTAAGAAAGTATTCTGTTAATTTTCAAAATTAAAAGTG--TTAAAAAGATTT
sp350 ACTTTTTTAAGAAAATATTCTATTAATTTTCAAGGTTAAAAGTG-CTTATAAAGATTT
sp352 AATAGTTTAAGAACGTATTCTGTTAATTTTCAAAGTTAAAAGTGTTTTATAAAGATTT
sp475 ATTGTTTTAAGAAAGTATTCTGTTAATTTTCAAAGTTAAAAGTG--TTGAAAAGATTT
sp479 AATAATTTAAGAAAATATTCTGTTAATTTTCAAAGTTAAAAGTG--CTAAAAAGATTT

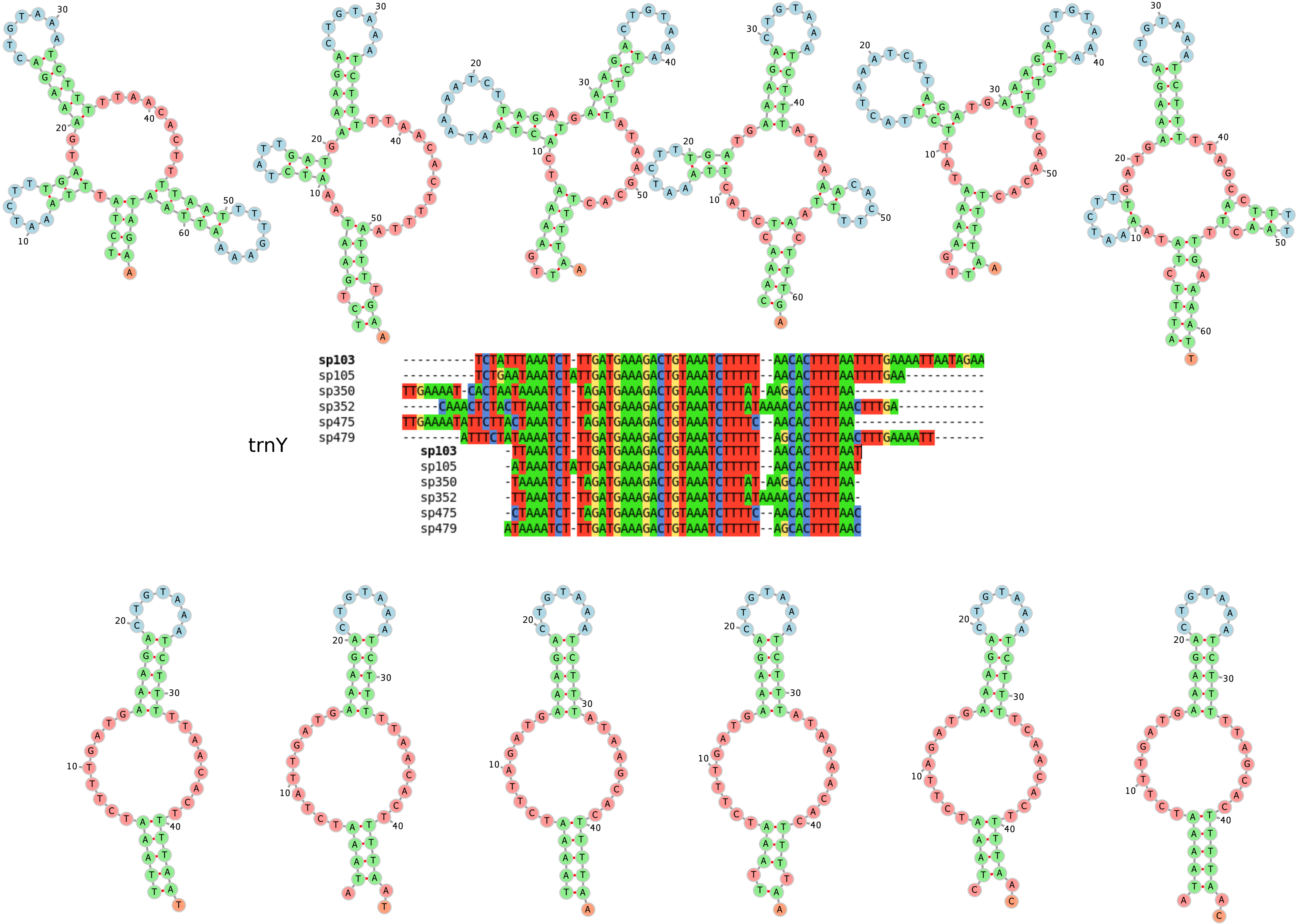
trnY
sp103 ----------TCTATTTAAATCT-TTGATGAAAGACTGTAAATCTTTTT--AACACTTTTAATTTTGAAAATTAATAGAA
sp105 ----------TCTGAATAAATCTATTGATGAAAGACTGTAAATCTTTTT--AACACTTTTAATTTTGAA----------
sp350 TTGAAAAT-CACTAATAAAATCT-TAGATGAAAGACTGTAAATCTTTAT-AAGCACTTTTAA------------------
sp352 -----CAAACTCTACTTAAATCT-TTGATGAAAGACTGTAAATCTTTATAAAACACTTTTAACTTTGA----------
sp475 TTGAAAATATTCTTACTAAATCT-TAGATGAAAGACTGTAAATCTTTTC--AACACTTTTAA------------------
sp479 --------ATTTCTATAAAATCT-TTGATGAAAGACTGTAAATCTTTTT--AGCACTTTTAACTTTGAAAATT-------
sp103 -TTAAATCT-TTGATGAAAGACTGTAAATCTTTTT--AACACTTTTAAT
sp105 -ATAAATCTATTGATGAAAGACTGTAAATCTTTTT--AACACTTTTAAT
sp350 -TAAAATCT-TAGATGAAAGACTGTAAATCTTTAT-AAGCACTTTTAA-
sp352 -TTAAATCT-TTGATGAAAGACTGTAAATCTTTATAAAACACTTTTAA-
sp475 -CTAAATCT-TAGATGAAAGACTGTAAATCTTTTC--AACACTTTTAAC
sp479 ATAAAATCT-TTGATGAAAGACTGTAAATCTTTTT--AGCACTTTTAAC
