# Supplementary material for: Arm-less mitochondrial tRNAs conserved for over 30 millions of years in spiders
Source: BMC Genomics. 2019 Aug 23;20:665. doi: 10.1186/s12864-019-6026-1 (PMC6706885; doi:10.1186/s12864-019-6026-1)
Supplement: Supplementary file 6 — Table S2. Primer list used to amplify long fragments 5′ cox1–3′ rrnL (9,5 kb). Parachtes teruelis (sp103), P. riberai (sp105), P. romandiolae (sp352), P. limbarae (sp475), P. ignavus (sp479), and Harpactocrates apennicola (sp350). Note that reverse primer was identical for all species except for P. riberai (sp105). (DOC 13 kb) [file 12864_2019_6026_MOESM6_ESM.doc]

**Additional File 6: Table S2.** Primer list used to amplify long fragments 5' cox1 – 3' rrnL (9,5 Kb). *Parachtes teruelis* (sp103), *P. riberai* (sp105), *P. romandiolae* (sp352), *P. limbarae* (sp475), *P. ignavus* (sp479), and *Harpactocrates* *apennicola* (sp350). Note that reverse primer was identical for all species except for *P. riberai* (sp105)..

Species sequence 5'-3'

Parachtes 103cox1Flong TTGGTTTATTAGGGTTTGTTGTTTG

Parachtes 350cox1Flong TTGGGGTGTTAGGGTTTGTG

Parachtes 352cox1Flong GGGGTATTAGGGTTTGTGGTGTG

Parachtes 475cox1Flong TTGGGTTGTTAGGGTTTGTTGTATG

Parachtes 479cox1Flong TTGGTGTGTTAGGGTTTGTGGTG

Parachtes Universal_16SRlong TGTAAGCCAGGTCGGTTTCTATCT

Parachtes 105cox1_F2 TATTAGTTATTCGGCTGGTAAACGGGAG

Parachtes 105_16S_R2 TTATTGGACTTCTACAACCATTCAGAGACGC

Primer list used to amplify short fragments 5' rrnL – 3' cox1 (4,5 Kb). Note that forward primer was identical for all species.

Species sequence 5'-3'

Parachtes UniversalSP16SFlong AGATAGAAACCGACCTGGCTTACACCG

Parachtes 103coxRlong GCCCAAACAACAAACCCTAATAAACC

Parachtes 350coxRlong CCCACACCACAAACCCTAACACCC

Parachtes 352coxRlong GCCCACACCACAAACCCTAATACCCC

Parachtes 475coxlong GCCCATACAACAAACCCTAACAACCC

Parachtes 479coxRlong GCTCACACCACAAACCCTAACACACC

Parachtes 105coxRlong CCATACGACAAACCCTAATACGCCGAT
